# Lp(a) Has Specific Effects on Coronary Artery Disease Independent of LDL-C: A Mendelian Randomization Study

**DOI:** 10.1016/j.jacadv.2026.102697

**Published:** 2026-03-25

**Authors:** Moa P. Lee, Sarah H. Koenigsberg, Mohammad Y. Anwar, Laura M. Raffield, Zhaotong Lin, Anna F. Ballou, John N. Booth, Thibaut Davy-Mendez, Helina Kassahun, Shia T. Kent, J. Antonio G. López, Keri L. Monda, Kari E. North, Rina Yarosh, Christie M. Ballantyne, Misa Graff, Christy L. Avery

**Affiliations:** a Department of Epidemiology, Gillings School of Global Public Health, University of North Carolina, Chapel Hill, North Carolina, USA; b Department of Genetics, University of North Carolina, Chapel Hill, North Carolina, USA; c Department of Statistics, Florida State University, Tallahassee, Florida, USA; d Center for Observational Research, Amgen Inc., Thousand Oaks, California, USA; e Division of Infectious Diseases, School of Medicine, University of North Carolina, Chapel Hill, North Carolina, USA; f Global Development, Amgen Inc., Thousand Oaks, California, USA; g Department of Medicine, Baylor College of Medicine, Houston, Texas, USA; h Carolina Population Center, University of North Carolina, Chapel Hill, North Carolina, USA

**Keywords:** atherosclerotic cardiovascular disease, lipoprotein(a), Mendelian randomization, phenome-wide association study, type 2 diabetes

## Abstract

**Background:**

Mendelian randomization studies suggest a causal effect of lipoprotein(a) (Lp(a)) on atherosclerotic cardiovascular disease. Noncardiovascular effects (eg, diabetes risk) are inadequately investigated.

**Objectives:**

In this noninterventional phenome-wide association study designed to better understand the potential causal role of Lp(a), direct causal phenotypic effects of exposure to Lp(a) were estimated. Also, the association between *LPA* null allele rs41272114 with type 2 diabetes was assessed, and ancestry-specific Lp(a) thresholds were determined.

**Methods:**

In the UK Biobank (n = 425,677 adults, 55% female), we studied 1,456 phenotypes spanning 18 classes using 4 ancestry-specific polygenic risk scores and false discovery rate multiple testing correction. Network deconvolution Mendelian randomization was leveraged to separate direct from indirect (ie, associations via mediating variables) causal phenotypic effects and account for confounding, reverse causation, and bidirectionality.

**Results:**

Lp(a) was significantly associated with 80 phenotypes across 7 classes. Higher Lp(a) exposure had significant direct causal effects, independent of low-density lipoprotein cholesterol, on coronary artery disease (OR: 1.36; 95% CI: 1.21-1.54) and glycated hemoglobin (HbA1c; β = 0.099; 95% CI: 0.051-0.15) only. Very low Lp(a) exposure was not associated with type 2 diabetes (OR: 0.92; 95% CI: 0.64-1.31) or HbA1c (β = −0.016; 95% CI: −0.062 to 0.030). Among European and African ancestries, 86 (77th percentile) and 93 (59th percentile) nmol/L optimally discriminated myocardial infarction risk, respectively.

**Conclusions:**

Increasing Lp(a) exposure had direct, independent causal effects on coronary artery disease and HbA1c only; very low Lp(a) exposure is suggested to not be causally associated with type 2 diabetes. The optimal European and African ancestry threshold to stratify cardiovascular risk is comparable, and below 125/105 nmol/L in current U.S./European medical professional society guidelines.

Lipoprotein(a) (Lp(a)) is a highly heritable (h^2^ = 70 to 90%), atherogenic, prothrombotic, and proinflammatory lipoprotein composed of 1 low-density lipoprotein (LDL)-like particle covalently bound to 1 apolipoprotein(a) particle.^1^, ^2^, ^3^ First detected in 1963,^4^ numerous studies have reported associations between elevated Lp(a) and increased atherosclerotic cardiovascular disease (ASCVD) risk.^5^^,^^6^ Clinical, regulatory, and public health interest in Lp(a) has been further bolstered by ongoing clinical trials of therapies that can specifically lower Lp(a) by up to 95%.^7^^,^^8^ However, Lp(a)’s potential causal roles in noncardiovascular diseases remain incompletely characterized.

Because Lp(a) is highly heritable, has a high population burden (for example, ∼20% of the global population have Lp(a)≥100 to 125 nmol/L),^9^ and the ability to study experimentally is limited, the current evidence base is highly reliant on genetic studies in human populations.^10^ This body of literature includes Mendelian randomization (MR) causal inference studies of individual phenotypes and phenome-wide association studies (PheWAS) of hundreds to thousands of phenotypes.^11^, ^12^, ^13^, ^14^ Despite studies suggesting that Lp(a) may have broad phenotypic effects, MR and PheWAS have prioritized ASCVD phenotypes.^15^, ^16^, ^17^, ^18^, ^19^, ^20^, ^21^ When broader groups of phenotypes were considered, few efforts have attempted to distinguish phenotypes directly affected by Lp(a) from phenotypes representing downstream or bidirectional effects. Finally, despite long documented ancestry-based differences in Lp(a),^22^^,^^23^ Lp(a) MR and PheWAS studies remain Eurocentric. These evidence gaps constrain our ability to understand potential mechanisms of action and conduct pharmacovigilance studies of therapeutic Lp(a) lowering.^7^^,^^8^^,^^24^

In the present noninterventional study, we sought to identify and characterize the impact of Lp(a) on broad phenotypic classes in 4 populations from the UK Biobank study. Our results help characterize the role of Lp(a) in coronary artery disease (CAD) incidence and precursor phenotypes and show how these roles are independent of LDL cholesterol (LDL-C) (Central Illustration). We anticipate that future efforts that expand phenotypic characterization and the representation of ancestrally diverse populations will further increase the reach and relevance of the Lp(a) evidence base.

**Figure 1:**
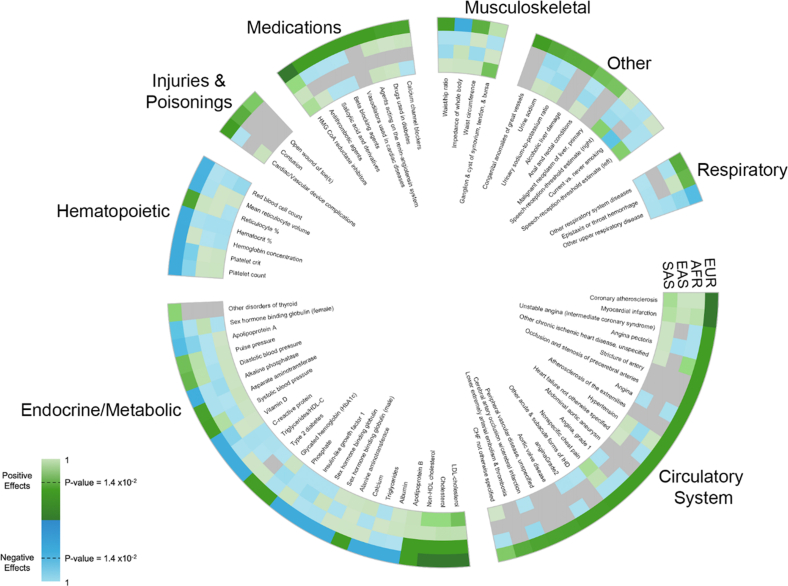
**Phenome-Wide Association Study of Continuous Lipoprotein(a) Identified 80 Significant Associations** Each ring in the figure represents a population with a distinct ancestral background. Positive effects are in green, negative effects in blue, and darker shading indicates more significant results. Population-specific associations with insufficient data are shown in gray. All statistical models were adjusted for ancestral PC, age, sex, and study center. The regression coefficients and *P* values for the tested associations are presented in Supplemental Table 4. AFR = African; CHF = congestive heart failure; EAS = East Asian; EUR = European; HDL-C = high-density lipoprotein cholesterol; IHD = ischemic heart disease; LDL = low-density lipoprotein; SAS = South Asian.

**Figure 2:**
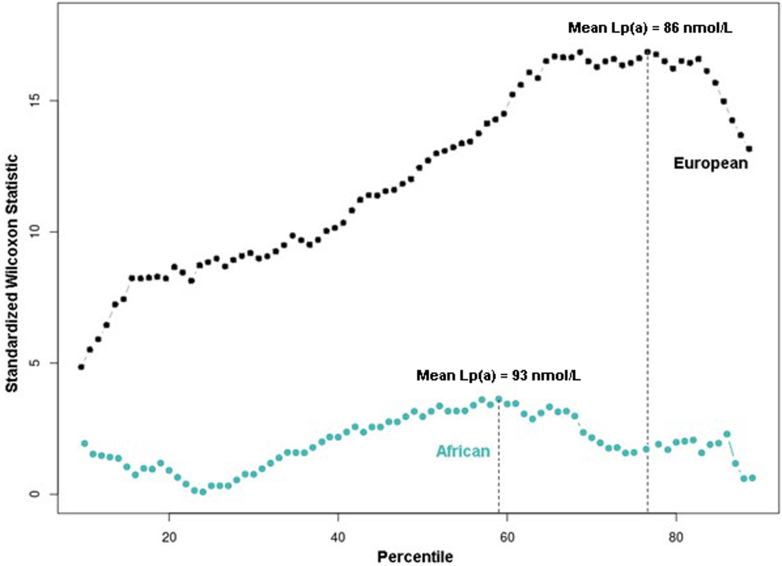
**Optimal Lp(a) Polygenic Risk Score Thresholds That Maximize Differences in Coronary Atherosclerosis Risk** Results are presented for UK Biobank participants of European (black circles) and African (teal circles) ancestry. Lp(a) = lipoprotein(a).

**Figure 3:**
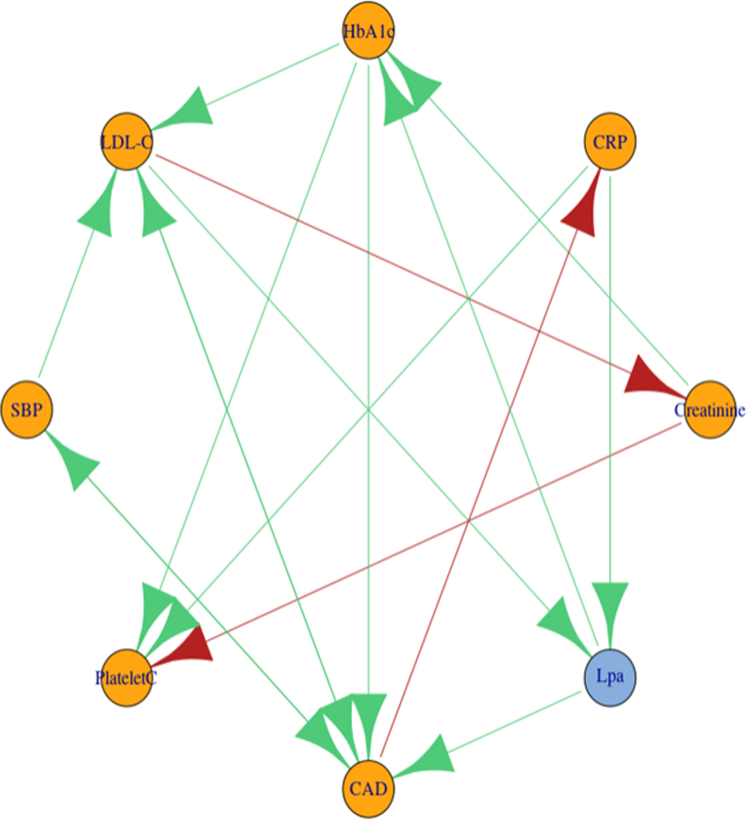
**Direct Causal Effects for 7 Phenotypes Identified in Lipoprotein(a) Phenome-Wide Association Study** Green edges show positive causal direct effects, and red lines show negative causal direct effects. CAD = coronary atherosclerosis; CRP = C-reactive protein; HbA1c = glycated hemoglobin; LDL-C = low-density lipoprotein cholesterol; PlateletC = platelet count; SBP = systolic blood pressure; other abbreviation as in Figures 1 and 2.

**Central Illustration:**
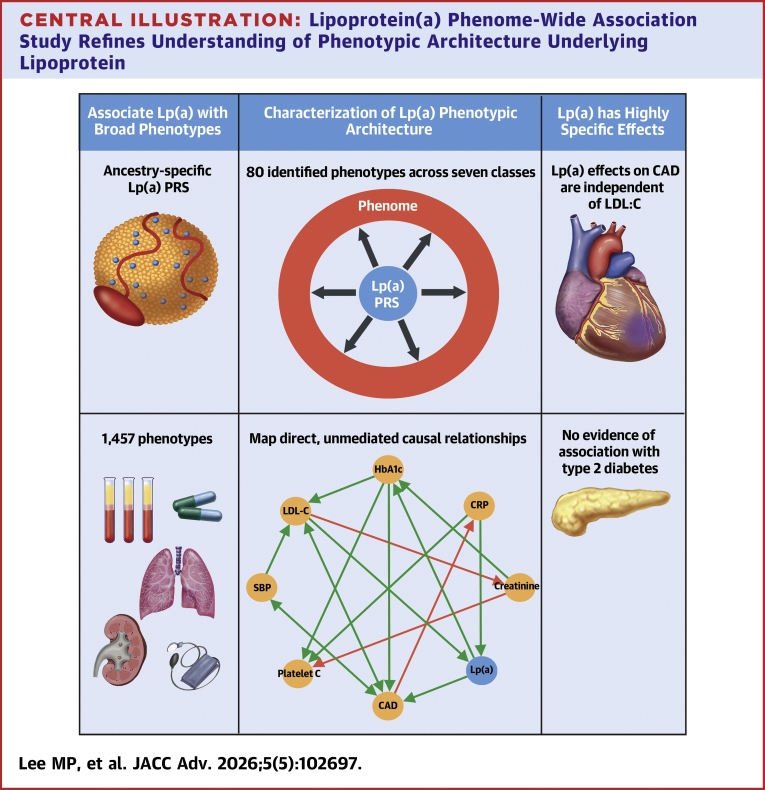
**Lipoprotein(a) Phenome-Wide Association Study Refines Understanding of Phenotypic Architecture Underlying Lipoprotein** CAD = coronary atherosclerosis; CRP = C-reactive protein; HbA1c = glycated hemoglobin; LDL-C = low-density lipoprotein cholesterol; Lp(a) = lipoprotein(a); PlateletC = platelet count; PRS = polygenic risk score; SBP = systolic blood pressure.

## Methods

### Study populations

The UK Biobank is a large, prospective study (2006-present) with extensive genetic and phenotypic data collected on 502,649 participants aged 40 to 69 years at 22 assessment centers throughout England, Wales, and Scotland.^25^ At the baseline exam, participants completed questionnaires assessing their social background, lifestyle, cognitive, physical functioning, mood, and personality; blood, urine, and saliva samples were also collected. All participants were followed up through linkage to health and medical records and national death registries. Eligible participants had genotypic data, measured Lp(a), and could be categorized into one of 4 populations defined by self-reported ancestry and genetic background (European, African, South Asian, or East Asian). In addition, we restricted analyses to unrelated participants using a kinship coefficient threshold of 0.0625 indicating third-degree relative pairs. This study was approved by the Institutional Review Board of the University of North Carolina, and all participants provided informed consent.

### Genetic instruments for Lp(a)

#### Genotyping and imputation

Genotyping was previously performed using 2 similar genotyping arrays, the UK Biobank Lung Exome Variant Evaluation and UK Biobank Axiom arrays by Affymetrix, with imputation using version 3 of the UK Biobank imputed data using IMPUTE2. Imputation was carried out primarily using the Haplotype Reference Consortium reference panel, and variants not featured on this panel were imputed with the UK 10K and 1000G reference panels.^26^ In addition to previously reported quality control protocols,^27^^,^^28^ we excluded variants meeting any of the following standard ancestry-specific criteria: minor allele frequency (MAF) <0.001; imputation quality score <0.4; or effective minor allele count defined as N_eff_<30: N_eff =_ *2f(1-f)Nq*, where f is the estimated MAF, N is the sample size, and q is the imputation quality.

#### Measured and genetically inferred Lp(a) levels

We used measured Lp(a) to calculate central tendency measures. For Lp(a) values below the 3.8 nmol/L limit of detection, we imputed a random value between 0 and 3.8 nmol/L assuming a uniform distribution. For Lp(a) values above the 189 nmol/L limit of detection, serial dilutions were performed and the samples were reanalyzed.^29^ These data were available in UK Biobank return 2321 and were used herein. To examine Lp(a) phenotypic effects, we used continuous and categorical ancestry-specific polygenic risk scores (PRSs)^30^ as well as specific variants (eg, rs41272114) to examine Lp(a) phenotypic effects. A categorical PRS was used to examine effects of genetically elevated Lp(a). To define genetically elevated Lp(a), we calculated population-specific mean measured Lp(a) values by PRS percentile and then dichotomized the PRS at the first percentile for which the mean measured Lp(a) exceeded 125 nmol/L. Genetically elevated Lp(a) was examined only in European and African populations because there were too few East and South Asian participants with measured Lp(a) values above the 125 nmol/L threshold to dichotomize the PRS.

### Phenotypic data

#### Investigator-defined phenotypes

For the investigator-defined phenotypes (Supplemental Table 1), each of the 253 UK Biobank–defined phenotype categories was manually reviewed by 2 authors (C.L.A. and R.Y.) and flagged if they potentially included relevant phenotypic data (through December 31, 2022). Nutrition/dietary phenotypes identified as “blob/bulk” and phenotypes measured in fewer than ∼10,000 participants were excluded from further review. After excluding phenotypes unlikely to be affected by Lp(a) (eg, variables describing the type of bread eaten yesterday, occupation codes, water hardness etc.), remaining phenotypes were then manually reviewed, renamed, and classified into a composite outcome as appropriate (eg, angina classified as Rose’s Angina Questionnaire; claudication classified using the Edinburgh claudication questionnaire). Phenotypes were then compared to distributions provided online and in the published literature, when possible, as a final quality control step.

#### Inpatient International Classification of Diseases-10th Revision codes

The inpatient diagnoses captured by the International Classification of Diseases-10th Revision (ICD-10) codes were not evaluated individually, but instead aggregated and mapped to clinically relevant traits using a phecode algorithm (Supplemental Table 2).^31^^,^^32^ We restricted to ICD-10 codes in the primary position, which represents the medical conditions that are chiefly responsible for the inpatient admission of the participant rather than the medical conditions potentially carried over from previous visits or medical history often reported in the diagnosis codes in the secondary position.^33^^,^^34^ ICD-10 data from 1997 to 2021 were available during our analyses. Missing data were negligible and when present, generally reflected study design choices, for example, measurement in a select population.

### Statistical analysis

PheWAS was conducted using a generalized linear modeling framework in each population considering a continuous PRS, and a PRS dichotomized at 125 nmol/L to define elevated Lp(a). For binary phenotypes (eg, type 2 diabetes [T2DM]), we used logistic regression, and for continuous phenotypes (eg, systolic blood pressure [SBP]), we used linear regression: $g\left(E\left[Y_{i}\right]\right)=\alpha_{0}+{\;\alpha }_{1}X_{i}+\alpha_{2}Z_{i}$. $Y_{i}$ and $X_{i}$ denoted the phenotype and the PRS for the $i^{th}$ individual, respectively. $Z_{i}$ denoted the potential confounders (15 ancestral principal components, age, sex, and study center), and *g* was the link function. All models were corrected for multiple comparisons within each population using a false discovery rate threshold of 0.05, a method that is robust to positively correlated test statistics.^35^ As a sensitivity analysis, we identified the optimal Lp(a) PRS threshold that maximized the difference in myocardial infarction (MI) risk when the continuous PRS was dichotomized (> vs <) at each percentile (10% to 90% to ensure a minimum proportion of 10% of observations in each group) using maximally selected rank statistics based on the Wilcoxon rank statistic. Corrected minimal *P* values for testing multiple thresholds were obtained based on the asymptotic null distribution of maximally selected rank statistic derived by Lausen and Schumacher.^36^^,^^37^

To help interpret PheWAS findings and distinguish direct from total causal effects, we applied network deconvolution MR.^38^ This approach was needed because if there are strong total causal effects for Lp(a) with phenotype A and strong total causal effects for phenotype A with phenotype B, total causal effects for Lp(a) with phenotype B also will be identified, even if there is no direct effect of Lp(a). Network deconvolution MR was also used to identify bidirectional relationships, which have been previously suggested for Lp(a).^17^^,^^39^ Network deconvolution MR required strong independent instrumental variables (IVs) for Lp(a) and each PheWAS-identified phenotype. To identify IVs for PheWAS-identified phenotypes, we conducted genome-wide association studies in UK Biobank European ancestral participants. For Lp(a) IVs, we used the 43 independent variants that composed the Lp(a) PRS.^12^ We then restricted to genome-wide significant (restricting to variants with *P* < 5 × 10^−8)^ common (MAF>1%) variants that were independent. Independence was determined using the 1,000 Genomes Phase 3 EUR population and the R ieugwasr package.^40^ Five thousand data perturbations were conducted to generate final estimates and perform statistical inference of the directed causal graph. To protect against weak IV bias, all phenotypes had conventional F-statistics>10. To protect against confounding, ancestral principal components, age, sex, and study center were included as covariates in genome-wide association studies. Bonferroni correction was used to determine the significance of direct causal effects for Lp(a). Network deconvolution MR was only performed in the European population, given anticipated low statistical power in the other 3 populations.

## Results

A maximum of 425,677 unrelated UK Biobank participants were included in the final analysis, representing European (n = 405,766), African (n = 8,490), South Asian (n = 9,039), and East Asian (n = 2,382) populations. Participants were on average middle-aged (mean = 56.6, SD = 8.1) and 54% of participants were females. Distributions of measured Lp(a) showed marked heterogeneity by ancestry, with the lowest medians in East Asians and the highest in Africans (Table 1). After mapping the 90,835 distinct ICD-10 codes to 1 of 1,755 phecodes and excluding categorical phenotypes with fewer than 20 cases by population, a maximum of 1,456 phenotypes (260 investigator-collected and 1,197 phecode-defined phenotypes) spanning 18 classes were included (Supplemental Table 3).

**Table 1:** Characteristics of 425,677 UK Biobank Participants by Population

	European (n = 405,766)	African (n = 8,490)	South Asian (n = 9,039)	East Asian (n = 2,382)
Mean age (SD), y	56.8 (8.0)	51.8 (8.1)	53 (8.5)	52 (7.8)
Female, %	54	58	46	66
Lipoprotein (a) levels				
Mean (SD)	55.3 (76.2)	97.5 (81.8)	55.1 (65.7)	37.7 (53.0)
Median (25th, 75th percentile)	18.6 (7.4, 73.2)	73.2 (41.2, 132.9)	32.2 (12.2, 70.8)	16.2 (8.0, 44.0)
>125 nmol/L, %	16.5	27.2	12.0	7.1
Lipoprotein (a) variance explained by ancestry-specific PRS (R^2^%)^27^	50	27	20	15

PRS = polygenic risk score.

A total of 80 phenotypes (5%) were significantly associated with the continuous Lp(a) PRS in African, European, or South Asian populations (Figure 1, Supplemental Table 4), of which 76 associations were identified only in the European population. Consistent with Lp(a)’s reported atherogenicity,^41^ 22 (27%) of significant associations mapped to the circulatory system class and included positive associations with CAD, carotid atherosclerosis, abdominal aortic aneurysm, and peripheral vascular disease. None of these circulatory system class phenotypes were significant in African, East Asian, or South Asian populations despite estimated effects being consistent across populations (eg, estimated ORs for MI incidence of 1.12-1.35 per SD PRS increase). Risk-increasing associations with heart failure, aortic valve disease, and congenital anomalies of great vessels were also identified. Further evaluation of the congenital anomalies of great vessels association demonstrated that 96.5% of the ICD-10 codes mapped to nonrheumatic aortic valve stenosis (ICD-10 code I35.0), an adult-onset disease. The association with Lp(a) was not significant (*P* value = 0.6) after cases of nonrheumatic aortic valve stenosis were removed.

Many of the remaining significant associations (n = 25, 31%) mapped to the endocrine/metabolic class. Increased Lp(a) PRS were associated with lower C-reactive protein (CRP), calcium, and other disorders of the thyroid. In contrast, increased Lp(a) PRS was associated with increased T2DM risk as well as increases in hemoglobin A1c (HbA1c), SBP, and alanine aminotransferase. Risk-increasing Lp(a) PRS associations with T2DM and HbA1c were observed. To more comprehensively evaluate these observations, we also examined associations between very low Lp(a) with T2DM and HbA1c by comparing European participants homozygous for the rs41272114 *LPA* null allele (n = 658; median (25th, 75th percentile) Lp(a): 2.1 nmol/L (1.00 nmol/L, 2.9 nmol/L) with European participants who carried 0 copies of the rs41272114 *LPA* null allele (n = 375,232; median (25th, 75th percentile) Lp(a): 20.4 nmol/L [8.1-78.9 nmol/L]). No statistical difference was observed in the odds of diabetes for participants carrying two rs41272114 *LPA* null alleles (32 cases of diabetes) compared to participants carrying 0 copies of the rs41272114 *LPA* null allele (19,972 cases of diabetes; OR: 0.92; 95% CI: 0.64-1.31). Similarly, null effects were estimated for HbA1c (β = −0.016; 95% CI: −0.062 to 0.030) for the same contrast.

We then conducted a PheWAS of genetically elevated Lp(a) (dichotomized at 125 nmol/L) and identified 55 phenotypes spanning 9 classes with significant associations. Results for the elevated Lp(a) PheWAS were largely consistent with results for the continuous PRS (Supplemental Table 5). As a sensitivity analysis, we examined whether the optimal Lp(a) PRS threshold for maximizing the difference in MI risk differed by population. The optimal Lp(a) PRS percentile threshold that maximized the differences in the MI risk was the 77th %ile (OR: 1.46; 95% CI: 1.40-1.53) in the European population, which corresponded to a mean Lp(a) of 86 nmol/L (Figure 2). In the African population, the 59th %ile (OR: .98; 95% CI: 1.32-2.97), corresponding to a mean Lp(a) of 93 nmol/L, was the optimal PRS percentile threshold that maximized the difference in MI risk.

Finally, we used network deconvolution MR to distinguish Lp(a) direct and total causal effects. Because network deconvolution methods are not scalable to all 80 significant phenotypes, we prioritized 7 phenotypes spanning phenotype classes for which strong IVs (F statistic>10) were available: serum creatinine, CRP, HbA1c (as a diabetes biomarker), LDL-C, Lp(a), SBP, platelet count, and CAD. CAD (OR: 1.36; 95% CI: 1.21-1.54) and HbA1c (β = 0.099; 95% CI: 0.051-0.15) were the only phenotypes for which Lp(a) had a significant direct causal effect after removing all possible mediating effects through the other included phenotypes, including LDL-C (Figure 3). Accommodation of bidirectional relationships also identified LDL-C and CRP as having direct positive causal effects on Lp(a).

## Discussion

In this Lp(a) PheWAS of ancestrally diverse populations, we identified 80 significant associations across 7 phenotype classes. However, only 2 phenotypes, coronary artery disease and HbA1c, were directly affected by Lp(a). These effects were positive and independent of LDL-C. Our results also suggested that lower absolute Lp(a) thresholds of approximately 100 nmol/L may be more appropriate and equitable across ancestral populations for ASCVD primary prevention. As interest in Lp(a) continues to grow and with potential treatments on the horizon, our work suggests that Lp(a) phenotypic effects may be highly specific to atherosclerotic coronary disease. However, additional efforts to include more diverse ancestral populations and to better capture uncommon and rare phenotypes are needed to more comprehensively catalog the phenotypic landscape potentially affected by Lp(a).

Over the past decade, there has been increasing evidence that Lp(a) is a causal and independent ASCVD risk factor, albeit in the absence of experimental data.^42^ There is less consistent evidence on the role of Lp(a) in other phenotypes, including T2DM, despite concerns that treating Lp(a) to very low levels may increase disease risk.^11^^,^^43^^,^^44^ Our results suggesting that lifelong very low Lp(a) levels are not causally related to increased T2DM risk are inconsistent with results from Gudbjartsson et al.,^43^ who suggest that populations carrying 2 loss of function null alleles had an odds of diabetes that was 45% higher than participants carrying no loss of function null alleles. Our study and Gudbjartsson et al., had similar numbers of T2DM cases among null allele homozygote populations (32 and 37 cases), suggesting similar statistical power. Post hoc statistical power calculations in the Icelandic population suggest modest statistical power (35.4% post hoc statistical power) to detect a 45% relative increase in T2DM. Such modest statistical power underscores the challenges in examining genetic effects for rarer genotypes, even when the total population is large and again emphasizes the need for well-powered replication studies.^45^ Leveraging ancestral diversity may offer an efficient means for replication and more precise characterization of the relationship between very low Lp(a) with T2DM, as rs41272114 is considerably more frequent in some admixed American populations, including Peruvian populations (MAF = 18%).^46^

Our findings for T2DM with the continuous PRS remain challenging to interpret, especially when considering randomized control trial results. For example, in the ODYSSEY OUTCOMES trial of alirocumab in patients with acute coronary syndrome, although treatment had no overall effect on T2DM risk (HR: 0.95; 95% CI: 0.85-1.05), every 10 mg/dL decrease in Lp(a) from baseline increased T2DM risk by 7% in the alirocumab group.^47^ Inconsistencies between genetic causal inference studies and randomized controlled trials examining associations between lipoproteins and T2DM risk have been reported previously.^48^ Using LDL-C lowering as an example, genetic causal inference studies correctly predicted therapeutic associated increases in T2DM risk with statins,^49^^,^^50^ but predictions for PCSK9 inhibitors or ezetimibe were inconsistent with trial evidence.^51^, ^52^, ^53^ These discrepant findings may reflect several factors, including genetic IVs that do not fully align with the mechanism of pharmacologic inhibition, horizontal pleiotropy, and population heterogeneity.^48^^,^^54^

We could not more comprehensively evaluate Lp(a)’s potential causal effects on other high-priority phenotypes, including heart failure and stroke. Although the prevalence of heart failure is high,^55^ the genetic architecture of heart failure remains poorly characterized,^56^ leaving few IVs for direct effect estimation. We also note that the total effect of Lp(a) on heart failure estimated in the PheWAS was very modest; any direct effect would likely be reduced further by causal paths through CAD, a primary heart failure risk factor.^57^ For stroke, no significant associations were detected for phenotypes spanning cerebral atherosclerosis, cerebral ischemia, or cerebrovascular disease, among others. These findings may reflect an inability to distinguish between stroke subtypes^58^ or other population differences.^59^

Our systematic approach to comprehensively identify and characterize Lp(a) phenotypic effects in diverse populations represents a step forward in strengthening an evidence base predominantly among European populations. Increased ancestral diversity is especially needed when examining the performance of Lp(a) thresholds. For example, Patel et al. examined dose-response associations between a proposed 150 nmol/L Lp(a) risk-enhancing threshold with incident ASCVD. They reported HRs of 1.51 (95% CI: 1.45-1.57), 1.37 (95% CI: 1.05-1.81), and 1.13 (95% CI: 0.84-1.50) in European, South Asian, and African ancestry populations, respectively (*P* heterogeneity = 0.15).^22^ Although we acknowledge the reduced PRS accuracy in African compared to European ancestral populations, our results suggest that a lower absolute Lp(a) threshold may be more appropriate and equitable for ASCVD primary prevention. A lower absolute threshold is also consistent with the dose-response analyses in Patel et al., that showed a log-linear association between measured Lp(a) and ASCVD risk, with no evidence of a threshold. Furthermore, our result aligns with the Canadian Cardiovascular Society guidelines^60^ that target a lower 100 nmol/L for treatment or as a risk-enhancing cutoff when considering initiation of therapies to lower LDL-C.

### Study Limitations

Despite multiple strengths including an innovative causal inference paradigm, broad phenotype capture, and powerful PRS, our study has several limitations. First, PRS remained at least twice as accurate in the European population; the considerably larger sample size in the European population further widened statistical power differences. Nevertheless, all PRS performed well when used to define populations with elevated Lp(a)^30^ and together provide an important step forward in understanding the phenotypic consequences of Lp(a). Second, we were unable to assess the effects of other variants that produce very low Lp(a) because these variants were too infrequent. These variants are important to study, especially if they capture different mechanisms.^61^^,^^62^ Third, we identified several unexpected findings, including inverse total causal effects for Lp(a) with CRP and inverse direct effects of LDL-C with creatinine. Because we did not identify a direct effect of Lp(a) on CRP, this effect may operate through other indirect pathways and may have only been identified due to our study’s high statistical power. For the LDL-C and creatinine findings, independent MR studies also have reported inverse relationships between LDL-C and kidney function,^63^ although further evaluation would be outside the scope of this study. Fourth, network deconvolution MR could not include all identified phenotypes simultaneously. Multiphenotype MR is an area of active research, and we anticipate that new tools will emerge, enabling evaluation of expanded causal networks. Finally, we did not address whether therapeutic reduction of Lp(a) levels reduces ASCVD risk or what degree of Lp(a) reduction is required to achieve significant risk reductions. Also unaddressed is whether the therapeutic reduction of Lp(a) to very low levels is associated with an increased risk of T2DM.

Our results help clarify the causal role of Lp(a), including the importance of approaching Lp(a) and LDL-C as unique ASCVD risk factors. Also, these results suggest that very low levels of genetically inferred Lp(a) are not causally associated with diabetes risk. Furthermore, despite known ancestry-based differences in Lp(a) levels, the optimal thresholds for risk discrimination may be similar in the African and European ancestral populations; this threshold is lower than most clinical guidelines and professional societies advocate. We anticipate that these studies will continue to help prioritize or rule out pathophysiological mechanisms by which Lp(a) influences health and disease, assist with the development and evaluation of risk thresholds for diverse populations, and help design, evaluate, and implement clinical interventions on Lp(a).

## Funding support and author disclosures

This work was supported by UK Biobank application 25953. 10.13039/100002429Amgen Inc (Thousand Oaks, California) partially funded this study. The following grants also supported this study: R01HL152828 (Drs Avery, Ballou, and North), R01HL151152 (Drs Avery, Graff, and North), R01HG010297 (Drs Avery, Graff, and North), R01HG011345 (Drs Avery, Graff, and North), T32HL007055 (Lee), R01HL172887 (Drs Avery and Graff), and F32HL149256 (Dr Lee). Dr Avery has received research funding from 10.13039/100000050NIH/NHLBI and 10.13039/100002429Amgen Inc. Drs Booth, Kassahun, Kent, López, and Monda are employees and stockholders of 10.13039/100002429Amgen Inc. Dr Ballantyne has received research support from and is a consultant for Denka Seiken. All other authors have reported that they have no relationships relevant to the contents of this paper to disclose.
