# Supplementary material for: Lp(a) Has Specific Effects on Coronary Artery Disease Independent of LDL-C: A Mendelian Randomization Study
Source: JACC Adv. 2026 Mar 25;5(5):102697. doi: 10.1016/j.jacadv.2026.102697 (PMC13050038; doi:10.1016/j.jacadv.2026.102697)
Supplement: Supplemental Tables 3-5 [file mmc3.pdf]

Supplemental Table 3. Overview of 18 categories examined in PheWAS of Lp(a) in 357,096 UK Biobank participants.

Category	European	African	East Asian	South Asian
Circulatory System	145	39	9	42
Congenital Anomalies	28	0	0	0
Dermatologic	65	13	1	17
Digestive	128	49	18	48
Endocrine/Metabolic	116	49	38	47
Genitourinary	134	55	16	55
Hematopoietic	59	34	31	33
Infectious Diseases	37	11	1	9
Injuries & Poisonings	73	17	1	20
Medications	23	16	3	17
Mental And Behavioral Disorders	76	21	7	24
Musculoskeletal	110	39	21	39
Neoplasms	140	26	5	23
Neurological	77	17	9	20
Reproductive	37	19	8	19
Respiratory	74	19	1	22
Sense Organs	110	33	13	35
Symptoms	24	14	3	12
Total	**1456**	471	185	482

Supplemental Table 4. Lp(a) PRS-phenotye assocations study results using the PRS as a continuous predictor. FDR was used to correct for multiple testing. Cells are empty when there were fewer than 20 cases.

Phenotype	Phecode	Class	European				African				South Asian				East Asian			
			Estimate	SE	P	FDR	Estimate	SE	P	FDR	Estimate	SE	P	FDR	Estimate	SE	P	FDR
Abdominal aortic aneurysm	442.11	Circulatory System	0.170	0.030	1.41E-07	6.45E-06												
Abnormal function study of cardiovascular system	429.2	Circulatory System	0.000	0.090	0.37	0.94												
Abnormal heart sounds	396	Circulatory System	0.000	0.210	0.95	1.00												
Acute pericarditis	420.21	Circulatory System	0.040	0.070	0.50	0.95												
Acute vascular insufficiency of intestine	441.1	Circulatory System	0.000	0.070	0.47	0.95												
Age angina diagnosed		Circulatory System	-0.273	0.098	0.01	0.08												
Age deep vein thrombosis diagnosed		Circulatory System	0.083	0.157	0.60	0.97												
Age pulmonary embolism diagnosed		Circulatory System	0.104	0.283	0.71	0.97												
Aneurysm and dissection of heart	411.41	Circulatory System	0.030	0.060	0.57	0.97												
Aneurysm of artery of lower extremity	442.3	Circulatory System	0.180	0.070	0.01	0.14												
Aneurysm of iliac artery	442.2	Circulatory System	0.020	0.120	0.82	0.98												
Aneurysm of other specified artery	442.8	Circulatory System	0.000	0.080	0.58	0.97												
Angina		Circulatory System	0.046	0.007	1.44E-10	8.75E-09	-0.049	0.038	0.19	0.90	-0.027	0.037	0.46	0.94				
Angina pectoris	411.3	Circulatory System	0.110	0.010	8.12E-21	9.88E-19	-0.087	0.112	0.44	0.92	0.052	0.063	0.42	0.94	-0.359	0.308	0.24	0.84
Angina, grade 1		Circulatory System	0.048	0.010	4.66E-07	2E-05	-0.036	0.056	0.52	0.92	-0.028	0.056	0.62	0.94				
Angina, grade 2		Circulatory System	0.045	0.011	2.79E-05	9.47E-04	-0.060	0.048	0.21	0.91	-0.025	0.046	0.58	0.94				
Anomalous atrioventricular excitation	426.4	Circulatory System	-0.200	0.110	0.05	0.48												
Aortic aneurysm	442.1	Circulatory System	0.040	0.050	0.43	0.95												
Aortic valve disease	394.3	Circulatory System	0.130	0.030	0.00	0.00												
Arrhythmia (cardiac) NOS	427.5	Circulatory System	0.000	0.050	0.15	0.69												
Arterial dissection	442.4	Circulatory System	0.200	0.170	0.25	0.81												
Arterial embolism and thrombosis	444	Circulatory System	0.050	0.050	0.29	0.86												
Arterial embolism and thrombosis of lower extremity a	444.1	Circulatory System	0.140	0.040	0.00	0.01												
Arterial stiffness index		Circulatory System	-0.002	0.003	0.57	0.97	-0.009	0.017	0.59	0.92	0.001	0.016	0.95	0.98	-0.051	0.035	0.14	0.71
Arteritis NOS	446.9	Circulatory System	0.080	0.070	0.25	0.81												
Atherosclerosis	440	Circulatory System	0.200	0.080	0.01	0.15												
Atherosclerosis of aorta	440.9	Circulatory System	0.230	0.090	0.02	0.20												
Atherosclerosis of renal artery	440.1	Circulatory System	0.050	0.130	0.69	0.97												
Atherosclerosis of the extremities	440.2	Circulatory System	0.190	0.030	5.68E-10	3.32E-08												
Atrial fibrillation and flutter	427.2	Circulatory System	0.020	0.010	0.01	0.13	-0.305	0.123	0.01	0.34	-0.178	0.105	0.09	0.83				
Atrioventricular [AV] block	426.2	Circulatory System	0.000	0.120	0.62	0.97												
Atrioventricular block, complete	426.24	Circulatory System	0.000	0.040	0.13	0.67												
Bundle branch block	426.3	Circulatory System	0.000	0.080	0.58	0.97												
Cardiac arrest	427.42	Circulatory System	0.070	0.050	0.13	0.67												
Cardiac conduction disorders	426	Circulatory System	0.240	0.110	0.04	0.40												
Cardiac index		Circulatory System	-0.004	0.006	0.52	0.95	0.087	0.079	0.27	0.92	-0.043	0.057	0.44	0.94				
Cardiac output		Circulatory System	-0.003	0.006	0.57	0.97	0.092	0.074	0.21	0.91	-0.062	0.054	0.25	0.91				
Cardiomegaly	416	Circulatory System	0.000	0.080	0.86	0.99												
Cerebral aneurysm	433.5	Circulatory System	-0.100	0.060	0.07	0.57												
Cerebral artery occlusion, with cerebral infarction	433.21	Circulatory System	0.060	0.010	0.00	0.01	-0.200	0.120	0.10	0.66	-0.019	0.121	0.88	0.98				
Cerebral atherosclerosis	433.12	Circulatory System	0.050	0.180	0.77	0.97												
Cerebral ischemia	433.3	Circulatory System	0.000	0.090	0.96	1.00												
Cerebrovascular disease	433	Circulatory System	0.030	0.040	0.37	0.94												
Chronic pericarditis	420.22	Circulatory System	0.230	0.140	0.11	0.65												
Chronic pulmonary heart disease	415.2	Circulatory System	0.230	0.120	0.07	0.55												
Chronic vascular insufficiency of intestine	441.2	Circulatory System	0.100	0.150	0.47	0.95												
Chronic venous insufficiency [CVI]	456	Circulatory System	0.130	0.080	0.12	0.65												
Circulatory disease NEC	459.9	Circulatory System	0.000	0.070	0.51	0.95												
Congestive heart failure (CHF) NOS	428.1	Circulatory System	0.070	0.020	0.00	0.04	-0.065	0.159	0.68	0.93	0.001	0.122	0.99	1.00				
Coronary atherosclerosis	411.4	Circulatory System	0.170	0.000	8.47E-121	4.12E-118	0.051	0.076	0.51	0.92	0.097	0.038	0.01	0.33	0.095	0.155	0.54	0.92
Definite claudication		Circulatory System	0.073	0.027	0.01	0.12												
Disease of capillaries	448	Circulatory System	0.000	0.130	0.61	0.97												
Disease of tricuspid valve	394.7	Circulatory System	0.000	0.060	0.23	0.79												
Endocarditis	420.3	Circulatory System	0.030	0.050	0.50	0.95												
Essential hypertension	401.1	Circulatory System	0.020	0.020	0.31	0.88	-0.143	0.117	0.22	0.91	0.056	0.129	0.67	0.96				
First degree AV block	426.21	Circulatory System	0.080	0.060	0.16	0.72												
Frontal angle P-wave		Circulatory System	0.009	0.011	0.40	0.94												
Frontal angle QRS		Circulatory System	0.125	0.293	0.67	0.97												
Frontal angle T-Wave		Circulatory System	0.350	0.221	0.11	0.65												
Giant cell arteritis	446.5	Circulatory System	0.000	0.050	0.77	0.97												
Heart failure NOS	428.2	Circulatory System	0.120	0.020	3.74E-08	1.82E-06	-0.075	0.160	0.64	0.93	0.033	0.127	0.80	0.98				
Hemorrhage NOS	459.1	Circulatory System	-0.100	0.170	0.43	0.95												
Hemorrhoids	455	Circulatory System	0.000	0.000	0.42	0.95	0.001	0.056	0.98	1.00	0.012	0.052	0.82	0.98	-0.084	0.098	0.39	0.88
Hypersensitivity angiitis	446.3	Circulatory System	0.000	0.180	0.95	1.00												
Hypertension		Circulatory System	0.022	0.004	2.79E-08	1.40E-06	0.031	0.026	0.23	0.91	0.031	0.025	0.20	0.89	-0.083	0.050	0.09	0.70
Hypertensive chronic kidney disease	401.22	Circulatory System	0.110	0.050	0.04	0.38												
Hypertensive heart disease	401.21	Circulatory System	0.330	0.110	0.00	0.08												
Hypertrophic obstructive cardiomyopathy	425.11	Circulatory System	-0.200	0.140	0.06	0.51												
Hypotension	458	Circulatory System	0.000	0.190	0.99	1.00												
Hypotension NOS	458.9	Circulatory System	0.100	0.040	0.03	0.34												
Iatrogenic hypotension	458.2	Circulatory System	0.060	0.070	0.40	0.94												
Intracerebral hemorrhage	430.2	Circulatory System	0.000	0.030	0.98	1.00	-0.236	0.224	0.29	0.92	-1.921	0.213	1.00	1.00				
Intracranial hemorrhage	430	Circulatory System	0.080	0.070	0.26	0.82												
Left bundle branch block	426.32	Circulatory System	0.050	0.060	0.43	0.95												
Left ventricle end diastolic volume		Circulatory System	-0.001	0.005	0.77	0.97	0.021	0.072	0.77	0.97	-0.071	0.050	0.16	0.89				
Left ventricle end systolic volume		Circulatory System	0.000	0.005	0.95	1.00	-0.041	0.073	0.57	0.92	-0.042	0.052	0.41	0.94				
Left ventricle stroke volume		Circulatory System	-0.004	0.005	0.45	0.95	0.166	0.071	0.02	0.43	-0.080	0.052	0.12	0.89				
Left ventricular ejection fraction		Circulatory System	-0.004	0.006	0.53	0.95	0.160	0.077	0.04	0.56	-0.035	0.057	0.55	0.94				
Maximum carotid IMT		Circulatory System	-0.001	0.005	0.87	0.99	0.001	0.065	0.99	1.00	0.005	0.047	0.92	0.98				
Mean carotid IMT		Circulatory System	-0.004	0.005	0.45	0.95	-0.006	0.065	0.92	0.98	-0.003	0.046	0.95	0.98				
Mean heart rate		Circulatory System	0.002	0.006	0.73	0.97	-0.050	0.079	0.53	0.92	0.052	0.057	0.37	0.94				
Mitral valve disease	394.2	Circulatory System	0.000	0.030	0.70	0.97					-0.014	0.199	0.94	0.98				
Myocardial infarction	411.2	Circulatory System	0.170	0.000	8.90E-72	2.16E-69	0.183	0.100	0.07	0.62	0.112	0.053	0.03	0.56	0.299	0.256	0.24	0.84
Myocarditis	420.1	Circulatory System	0.060	0.100	0.52	0.95												
Noninfectious disorders of lymphatic channels	450	Circulatory System	0.000	0.060	0.45	0.95												
Nonrheumatic aortic valve disorders	395.2	Circulatory System	0.140	0.090	0.14	0.68												
Nonrheumatic mitral valve disorders	395.1	Circulatory System	0.000	0.030	0.59	0.97					0.065	0.213	0.76	0.98				
Nonspecific chest pain	418	Circulatory System	0.030	0.000	6.75E-07	2.66E-05	-0.045	0.037	0.23	0.91	0.073	0.031	0.02	0.40	0.124	0.093	0.18	0.73
Occlusion and stenosis of precerebral arteries	433.1	Circulatory System	0.210	0.030	1.44E-10	8.75E-09												
Occlusion of cerebral arteries	433.2	Circulatory System	0.020	0.010	0.09	0.60	-0.073	0.096	0.45	0.92	0.151	0.102	0.14	0.89				
Occlusion of cerebral arteries, with cerebral infarction	433.11	Circulatory System	0.110	0.060	0.08	0.57												
Orthostatic hypotension	458.1	Circulatory System	0.070	0.020	0.01	0.12	0.182	0.191	0.34	0.92	-0.028	0.184	0.88	0.98				
Other acute and subacute forms of ischemic heart dise	411.9	Circulatory System	0.120	0.020	2.29E-05	8.14E-04					-0.022	0.141	0.87	0.98				
Other cardiomyopathy	425.8	Circulatory System	0.130	0.170	0.43	0.95												
Other chronic ischemic heart disease, unspecified	411.8	Circulatory System	0.160	0.020	1.50E-16	1.29E-14	0.536	0.164	1.07E-03	0.08	0.013	0.106	0.90	0.98				
Other disorders of arteries and arterioles	447	Circulatory System	0.070	0.040	0.15	0.69												
Other disorders of circulatory system	459	Circulatory System	0.080	0.100	0.46	0.95												
Other forms of chronic heart disease	414	Circulatory System	0.060	0.050	0.25	0.81												
Other heart block	426.25	Circulatory System	0.000	0.110	0.79	0.98												
Other hypertrophic cardiomyopathy	425.12	Circulatory System	-0.100	0.120	0.18	0.75												
Other specified cardiac dysrhythmias	427.3	Circulatory System	0.040	0.020	0.10	0.62					-0.092	0.181	0.61	0.94				
Other specified peripheral vascular diseases	443.8	Circulatory System	0.220	0.180	0.21	0.78												
Other venous embolism and thrombosis	452	Circulatory System	0.000	0.060	0.65	0.97												
P-duration (from P-onset to P-offset)		Circulatory System	-0.012	0.133	0.93	1.00												
PQ duration (from P-onset to QRS-onset)		Circulatory System	0.012	0.011	0.24	0.80												
Palpitations	427.9	Circulatory System	0.020	0.020	0.25	0.81	-0.119	0.125	0.34	0.92	0.032	0.134	0.81	0.98				
Paroxysmal supraventricular tachycardia	427.11	Circulatory System	0.000	0.020	0.71	0.97	-0.121	0.188	0.52	0.92	-0.083	0.163	0.61	0.94				
Paroxysmal tachycardia, unspecified	427.1	Circulatory System	0.000	0.120	0.55	0.96												
Paroxysmal ventricular tachycardia	427.12	Circulatory System	0.070	0.030	0.06	0.49					0.252	0.211	0.23	0.89				
Pericarditis	420.2	Circulatory System	0.070	0.030	0.04	0.40	0.179	0.196	0.36	0.92	0.183	0.201	0.36	0.94				
Peripheral angiopathy in diseases classified elsewhere	443.7	Circulatory System	0.050	0.060	0.41	0.95												
Peripheral vascular disease, unspecified	443.9	Circulatory System	0.120	0.030	0.00	0.00												
Phlebitis and thrombophlebitis	451	Circulatory System	0.000	0.060	0.58	0.97												
Phlebitis and thrombophlebitis of lower extremities	451.2	Circulatory System	0.000	0.010	0.15	0.69	0.064	0.121	0.59	0.92	-0.182	0.181	0.31	0.93				
Precordial pain	418.1	Circulatory System	0.040	0.010	0.00	0.07	-0.072	0.090	0.42	0.92	-0.108	0.073	0.14	0.89	-0.022	0.238	0.93	0.99
Premature beats	427.6	Circulatory System	0.000	0.060	0.81	0.98												
Primary pulmonary hypertension	415.21	Circulatory System	0.070	0.100	0.48	0.95												
Primary/intrinsic cardiomyopathies	425.1	Circulatory System	0.020	0.040	0.52	0.95	0.108	0.221	0.63	0.92								
Pulmonary embolism and infarction, acute	415.11	Circulatory System	0.000	0.110	0.93	1.00												
Pulmonary heart disease	415	Circulatory System	0.000	0.010	0.57	0.97	-0.114	0.111	0.30	0.92	0.402	0.175	0.02	0.43				

QRS duration		Circulatory System	0.043	0.073	0.55	0.96												
QT interval (Bazett heart rate corrected)		Circulatory System	0.086	0.213	0.69	0.97												
RR interval		Circulatory System	-0.003	1.353	1.00	1.00												
Raynaud's syndrome	443.1	Circulatory System	0.140	0.080	0.10	0.62												
Rheumatic disease of the heart valves	394	Circulatory System	0.060	0.040	0.17	0.72												
Right bundle branch block	426.31	Circulatory System	0.130	0.100	0.19	0.75												
Second degree AV block	426.23	Circulatory System	0.030	0.040	0.46	0.95												
Self-reported deep vein thrombosis		Circulatory System	0.008	0.012	0.53	0.95												
Self-reported pulmonary embolism		Circulatory System	-0.002	0.024	0.93	1.00												
Self-reported venous thromboembolism		Circulatory System	0.006	0.011	0.60	0.97												
Sinoatrial node dysfunction (Bradycardia)	427.8	Circulatory System	0.000	0.040	0.23	0.79												
Stricture of artery	447.1	Circulatory System	0.220	0.030	0.00	0.00												
Subarachnoid hemorrhage	430.1	Circulatory System	0.000	0.030	0.93	1.00	-0.085	0.211	0.69	0.93								
Subdural hemorrhage	430.3	Circulatory System	0.010	0.050	0.72	0.97												
Supraventricular premature beats	427.61	Circulatory System	0.000	0.160	0.62	0.97												
Symptoms involving cardiovascular system	429.3	Circulatory System	0.000	0.090	0.81	0.98												
Tachycardia NOS	427.7	Circulatory System	0.010	0.050	0.73	0.97												
Transient cerebral ischemia	433.31	Circulatory System	0.050	0.020	0.00	0.07	0.038	0.171	0.82	0.98	0.173	0.131	0.19	0.89				
Unstable angina (intermediate coronary syndrome)	411.1	Circulatory System	0.160	0.010	1.14E-28	1.85E-26	-0.105	0.122	0.39	0.92	0.053	0.068	0.43	0.94				
Varicose veins	454	Circulatory System	0.020	0.050	0.63	0.97												
Varicose veins of lower extremity	454.1	Circulatory System	0.000	0.010	0.85	0.99	0.147	0.104	0.16	0.86	0.064	0.079	0.42	0.94	0.053	0.184	0.77	0.99
Varicose veins of lower extremity, symptomtic	454.11	Circulatory System	0.040	0.030	0.22	0.78												
Vascular insufficiency of intestine	441	Circulatory System	-0.100	0.060	0.07	0.55												
Ventricular fibrillation and flutter	427.41	Circulatory System	-0.100	0.100	0.14	0.69												
Wegener's granulomatosis	446.4	Circulatory System	0.060	0.080	0.41	0.95												
Anomalies of endocrine glands, congenital	759.1	Congenital Anomalies	0.140	0.090	0.15	0.70												
Anomalies of respiratory system, congenital	748	Congenital Anomalies	0.140	0.140	0.35	0.92												
Cardiac congenital anomalies	747.1	Congenital Anomalies	0.020	0.110	0.86	0.99												
Cardiac shunt/ heart septal defect	747.11	Congenital Anomalies	0.050	0.050	0.30	0.87												
Cleft palate	749.1	Congenital Anomalies	-0.100	0.240	0.49	0.95												
Congenital anomalies of esophagus	750.14	Congenital Anomalies	0.010	0.170	0.95	1.00												
Congenital anomalies of face and neck	749	Congenital Anomalies	0.000	0.070	0.69	0.97												
Congenital anomalies of female genital organs	751.11	Congenital Anomalies	-0.100	0.100	0.29	0.86												
Congenital anomalies of genital organs	751.1	Congenital Anomalies	-0.100	0.240	0.43	0.95												
Congenital anomalies of great vessels	747.13	Congenital Anomalies	0.200	0.020	6.68E-17	6.50E-15												
Congenital anomalies of intestine	750.21	Congenital Anomalies	0.070	0.110	0.52	0.95												
Congenital anomalies of male genital organs	751.12	Congenital Anomalies	0.000	0.110	0.53	0.95												
Congenital anomalies of mouth/tongue	750.13	Congenital Anomalies	0.000	0.070	0.53	0.95												
Congenital anomalies of peripheral vascular system	747.2	Congenital Anomalies	0.020	0.080	0.79	0.98												
Congenital anomalies of urinary system	751.2	Congenital Anomalies	0.000	0.180	0.61	0.97												
Congenital anomaly of gallbladder, bile ducts, liver, pa	750.22	Congenital Anomalies	0.080	0.150	0.58	0.97												
Congenital cataract and lens anomalies	753.1	Congenital Anomalies	0.150	0.120	0.23	0.79												
Congenital deformities of feet	755.1	Congenital Anomalies	0.070	0.080	0.35	0.92												
Congenital hip dysplasia and deformity	755.61	Congenital Anomalies	0.000	0.140	0.91	1.00												
Congenital musculoskeletal deformities of spine	754	Congenital Anomalies	-0.100	0.240	0.59	0.97												
Congenital osteodystrophies	756.5	Congenital Anomalies	0.020	0.060	0.70	0.97												
Cystic kidney disease	751.21	Congenital Anomalies	-0.100	0.110	0.28	0.86												
Esophageal atresia/tracheoesophageal fistula	750.11	Congenital Anomalies	-0.100	0.230	0.59	0.97												
Other and unspecified congenital anomalies	759	Congenital Anomalies	0.170	0.140	0.20	0.77												
Other congenital musculoskeletal anomalies	756	Congenital Anomalies	0.000	0.220	0.89	1.00												
Other specified congenital anomalies of nervous syster	752.2	Congenital Anomalies	0.000	0.150	0.99	1.00												
Spina bifida	752.11	Congenital Anomalies	0.030	0.140	0.82	0.98												
Valvular heart disease/ heart chambers	747.12	Congenital Anomalies	0.000	0.110	0.58	0.97												
Abnormal granulation tissue	701.5	Dermatologic	0.040	0.050	0.37	0.94												
Acne	706.1	Dermatologic	0.070	0.140	0.62	0.97												
Acquired acanthosis nigricans	701.6	Dermatologic	-0.200	0.150	0.10	0.63												
Actinic keratosis	702.1	Dermatologic	0.000	0.020	0.41	0.95												
Alopecia	704.1	Dermatologic	0.000	0.130	0.59	0.97												
Atopic/contact dermatitis due to other or unspecified	939	Dermatologic	0.000	0.030	0.84	0.99					-0.314	0.248	0.21	0.89				
Carbuncle and furuncle	686.1	Dermatologic	0.040	0.020	0.02	0.28	-0.172	0.100	0.08	0.66	-0.178	0.102	0.08	0.82				
Cellulitis and abscess of arm/hand	681.3	Dermatologic	0.010	0.010	0.44	0.95	0.066	0.117	0.58	0.92	-0.062	0.096	0.52	0.94				
Cellulitis and abscess of face/neck	681.2	Dermatologic	0.000	0.040	0.75	0.97												
Cellulitis and abscess of fingers/toes	681.1	Dermatologic	0.000	0.040	0.46	0.95												
Cellulitis and abscess of foot, toe	681.6	Dermatologic	0.010	0.010	0.44	0.95	0.066	0.117	0.58	0.92	-0.062	0.096	0.52	0.94				
Cellulitis and abscess of leg, except foot	681.5	Dermatologic	0.010	0.010	0.44	0.95	0.066	0.117	0.58	0.92	-0.062	0.096	0.52	0.94				
Cellulitis and abscess of trunk	681.7	Dermatologic	0.000	0.060	0.33	0.91												
Changes in skin texture	687.3	Dermatologic	0.110	0.150	0.47	0.95												
Chronic dermatitis due to solar radiation	938.2	Dermatologic	0.000	0.070	0.88	1.00												
Chronic ulcer of skin	707	Dermatologic	0.000	0.040	0.52	0.95												
Circumscribed scleroderma	701.3	Dermatologic	0.060	0.170	0.69	0.97												
Congenital anomalies of skin	691	Dermatologic	-0.100	0.160	0.23	0.79												
Corns and callosities	700	Dermatologic	-0.200	0.110	0.05	0.49												
Cutaneous lupus erythematosus	695.41	Dermatologic	-0.100	0.180	0.32	0.89												
Decubitus ulcer	707.1	Dermatologic	-0.200	0.110	0.08	0.59												
Dermatitis due to solar radiation	938	Dermatologic	-0.200	0.130	0.12	0.66												
Dermatomyositis	709.5	Dermatologic	0.190	0.140	0.18	0.75												
Diseases of hair and hair follicles	704	Dermatologic	0.000	0.010	0.46	0.95	-0.175	0.110	0.11	0.72	-0.223	0.101	0.03	0.51				
Diseases of nail, NOS	703	Dermatologic	-0.100	0.060	0.02	0.22												
Disorder of skin and subcutaneous tissue NOS	689	Dermatologic	0.000	0.010	0.74	0.97	0.026	0.119	0.83	0.98	-0.084	0.125	0.50	0.94				
Disturbance of skin sensation	687.4	Dermatologic	0.000	0.020	0.75	0.97	-0.043	0.177	0.81	0.97	0.027	0.168	0.87	0.98				
Erythema nodosum	695.81	Dermatologic	0.030	0.160	0.81	0.98												
Hidradenitis	705.3	Dermatologic	0.010	0.080	0.87	0.99												
Hyperhidrosis	705.8	Dermatologic	0.000	0.080	0.76	0.97												
Ingrowing nail	703.1	Dermatologic	0.020	0.030	0.44	0.95												
Keloid scar	701.4	Dermatologic	0.010	0.070	0.82	0.98	0.051	0.191	0.79	0.97								
Keratoderma, acquired	701.1	Dermatologic	0.000	0.150	0.78	0.98												
Other dyschromia	694.2	Dermatologic	0.000	0.030	0.96	1.00												
Other hypertrophic and atrophic conditions of skin	701	Dermatologic	0.000	0.020	0.77	0.97	-0.181	0.214	0.40	0.92	0.233	0.188	0.22	0.89				
Other local infections of skin and subcutaneous tissue	686	Dermatologic	0.000	0.030	0.32	0.89												
Other specified diffuse diseases of connective tissue	709.6	Dermatologic	0.140	0.190	0.47	0.95												
Other specified diseases of hair and hair follicles	704.8	Dermatologic	0.000	0.130	0.81	0.98												
Other specified erythematous conditions	695.8	Dermatologic	0.000	0.090	0.89	1.00												
Pemphigus and pemphigoid	695.22	Dermatologic	-0.100	0.140	0.38	0.94												
Pilonidal cyst	686.3	Dermatologic	0.010	0.040	0.75	0.97												
Polymyositis	709.4	Dermatologic	-0.100	0.200	0.47	0.95												
Prurigo and Lichen	695.7	Dermatologic	0.030	0.030	0.36	0.93					-0.137	0.210	0.51	0.94				
Pruritus and related conditions	698	Dermatologic	0.000	0.050	0.86	0.99												
Psoriasis	696.4	Dermatologic	0.000	0.060	0.99	1.00												
Psoriasis vulgaris	696.41	Dermatologic	0.000	0.050	0.86	0.99					-0.044	0.226	0.85	0.98				
Psoriatic arthropathy	696.42	Dermatologic	0.020	0.050	0.63	0.97												
Pyoderma	686.5	Dermatologic	0.120	0.180	0.52	0.95												
Pyogenic granuloma	686.4	Dermatologic	0.030	0.050	0.54	0.96												
Rash and other nonspecific skin eruption	687.1	Dermatologic	0.040	0.030	0.20	0.76					-0.052	0.193	0.79	0.98				
Rosacea	695.3	Dermatologic	0.000	0.090	1.00	1.00												
Sarcoidosis	697	Dermatologic	0.050	0.060	0.39	0.94	-0.076	0.198	0.70	0.94	-0.211	0.226	0.35	0.94				
Scar conditions and fibrosis of skin	701.2	Dermatologic	0.000	0.020	0.41	0.95					0.293	0.168	0.08	0.82				
Sebaceous cyst	706.2	Dermatologic	0.000	0.010	0.72	0.97	-0.098	0.090	0.28	0.92	-0.070	0.082	0.39	0.94	0.103	0.186	0.58	0.92
Seborrheic keratosis	702.2	Dermatologic	0.000	0.010	0.62	0.97					-0.182	0.237	0.44	0.94				
Sicca syndrome	709.2	Dermatologic	-0.100	0.120	0.12	0.65												
Superficial cellulitis and abscess	681	Dermatologic	0.010	0.050	0.79	0.98												
Symptoms affecting skin	687	Dermatologic	0.030	0.110	0.75	0.97												
Systemic lupus erythematosus	695.42	Dermatologic	0.050	0.090	0.55	0.96												
Systemic sclerosis	709.3	Dermatologic	0.130	0.100	0.18	0.75												
Toxic erythema	695.1	Dermatologic	0.000	0.110	0.99	1.00												
Unspecified diffuse connective tissue disease	709.7	Dermatologic	0.000	0.020	0.64	0.97	-0.055	0.130	0.67	0.93	0.040	0.140	0.77	0.98				
Unspecified erythematous condition	695.9	Dermatologic	0.090	0.070	0.20	0.76												
Urticaria	947	Dermatologic	0.020	0.040	0.69	0.97	0.316	0.177	0.07	0.62								
Vascular disorders of skin	694.3	Dermatologic	-0.100	0.130	0.28	0.86												
Abdominal hernia	550	Digestive	0.020	0.010	0.13	0.67	-0.064	0.116	0.58	0.92	0.106	0.138	0.44	0.94				
Abnormal results of function study of liver	573.7	Digestive	0.060	0.030	0.05	0.45												
Abnormal serum enzyme levels	573.9	Digestive	-0.100	0.120	0.27	0.83												
Acute appendicitis	540.11	Digestive	0.000	0.010	0.38	0.94	0.363	0.141	0.01	0.34	0.058	0.163	0.72	0.97				
Acute gastritis	535.1	Digestive	0.030	0.030	0.32	0.90	0.083	0.230	0.72	0.95								

Acute pancreatitis	577.1	Digestive	0.000	0.020	0.23	0.79	-0.011	0.208	0.96	0.99	-0.180	0.142	0.21	0.89				
Acute periodontitis	523.31	Digestive	0.000	0.100	0.39	0.94												
Anal and rectal conditions	565	Digestive	0.030	0.010	0.00	0.03	-0.015	0.078	0.85	0.98	0.006	0.063	0.92	0.98	-0.421	0.224	0.06	0.70
Anal and rectal polyp	565.1	Digestive	0.000	0.010	0.53	0.95	0.099	0.104	0.34	0.92	0.071	0.093	0.45	0.94	0.361	0.191	0.06	0.70
Anomalies of jaw size/symmetry	526.3	Digestive	-0.200	0.190	0.25	0.81												
Anomalies of tooth position/malocclusion	524.3	Digestive	0.010	0.060	0.82	0.98												
Appendiceal conditions	540	Digestive	0.000	0.080	0.95	1.00												
Appendicitis	540.1	Digestive	0.030	0.040	0.42	0.95												
Ascites (non malignant)	572	Digestive	0.030	0.040	0.41	0.95												
Atrophic gastritis	535.2	Digestive	0.000	0.060	0.90	1.00												
Atrophy of edentulous alveolar ridge	525.2	Digestive	0.160	0.120	0.19	0.75												
Bariatric surgery	539	Digestive	-0.300	0.270	0.27	0.84												
Blood in stool	578.2	Digestive	0.010	0.020	0.53	0.95	-0.014	0.161	0.93	0.98	0.480	0.141	0.00	0.05				
Calculus of bile duct	574.2	Digestive	0.000	0.020	0.00	0.07					-0.170	0.170	0.32	0.93				
Celiac disease	557.1	Digestive	0.010	0.030	0.75	0.97												
Cellulitis and abscess of oral soft tissues	528.3	Digestive	0.000	0.070	0.95	1.00												
Cholangitis	575.1	Digestive	0.000	0.040	0.76	0.97												
Cholecystitis without cholelithiasis	574.3	Digestive	0.000	0.010	0.84	0.99	-0.091	0.162	0.57	0.92	-0.038	0.127	0.77	0.98				
Cholelithiasis	574.1	Digestive	0.000	0.010	0.25	0.81	-0.094	0.109	0.39	0.92	-0.101	0.098	0.30	0.93	0.136	0.194	0.48	0.90
Cholelithiasis with acute cholecystitis	574.11	Digestive	0.000	0.020	0.01	0.09	-0.410	0.210	0.05	0.62	-0.172	0.185	0.35	0.94				
Cholelithiasis with other cholecystitis	574.12	Digestive	0.000	0.010	0.08	0.57	0.060	0.135	0.66	0.93	-0.033	0.103	0.75	0.98	-0.158	0.261	0.55	0.92
Cholesterolosis of gallbladder	575.6	Digestive	0.000	0.140	0.74	0.97												
Chronic pancreatitis	577.2	Digestive	0.000	0.050	0.50	0.95												
Chronic periodontitis	523.32	Digestive	0.000	0.030	0.81	0.98	0.079	0.167	0.64	0.93	0.142	0.176	0.42	0.94				
Cirrhosis of liver without mention of alcohol	571.51	Digestive	0.110	0.050	0.04	0.44												
Cyst and pseudocyst of pancreas	577.3	Digestive	0.000	0.050	0.96	1.00												
Cyst of the salivary gland	528.41	Digestive	-0.100	0.110	0.34	0.92												
Cysts of oral soft tissues	528.4	Digestive	0.140	0.190	0.47	0.95												
Cysts of the jaws	526.1	Digestive	0.000	0.060	0.95	1.00												
Dental caries	521.1	Digestive	0.000	0.010	0.79	0.98	-0.043	0.063	0.49	0.92	0.081	0.101	0.43	0.94				
Dentofacial anomalies, including malocclusion	524	Digestive	-0.100	0.200	0.46	0.95												
Diaphragmatic hernia	550.2	Digestive	0.000	0.000	0.30	0.88	-0.055	0.079	0.49	0.92	-0.037	0.067	0.58	0.94	-0.159	0.205	0.44	0.90
Diseases and other conditions of the tongue	529	Digestive	0.000	0.030	0.80	0.98												
Diseases of esophagus	530	Digestive	0.000	0.050	0.78	0.98												
Diseases of lips	528.5	Digestive	0.020	0.030	0.53	0.95												
Diseases of pancreas	577	Digestive	0.000	0.050	0.17	0.74												
Diseases of pulp and periapical tissues	522	Digestive	0.060	0.040	0.16	0.72												
Diseases of the oral soft tissues, excluding lesions spec	528	Digestive	0.040	0.020	0.02	0.27	-0.029	0.175	0.87	0.98	0.077	0.133	0.56	0.94				
Diseases of the salivary glands	527	Digestive	0.000	0.110	0.60	0.97												
Disorders of esophageal motility	530.5	Digestive	0.020	0.040	0.62	0.97												
Disorders of tooth development	520	Digestive	0.120	0.180	0.51	0.95												
Disturbance of salivary secretion	527.7	Digestive	-0.100	0.220	0.39	0.94												
Disturbances in tooth eruption	520.2	Digestive	0.000	0.020	0.77	0.97	0.193	0.156	0.22	0.91	0.143	0.150	0.34	0.94				
Diverticulosis	562.1	Digestive	0.000	0.000	0.94	1.00	-0.061	0.061	0.31	0.92	0.042	0.066	0.52	0.94	-0.107	0.216	0.62	0.93
Diverticulum of esophagus, acquired	530.6	Digestive	0.050	0.080	0.53	0.95												
Duodenal ulcer	531.3	Digestive	0.000	0.020	0.74	0.97	0.417	0.204	0.04	0.59	-0.102	0.147	0.49	0.94				
Duodenitis	535.6	Digestive	0.030	0.020	0.11	0.64	0.128	0.156	0.41	0.92	-0.010	0.124	0.94	0.98				
Dysphagia	532	Digestive	0.000	0.010	0.71	0.97	0.067	0.092	0.46	0.92	-0.040	0.081	0.62	0.94	0.347	0.151	0.02	0.57
Esophageal bleeding (varices/hemorrhage)	530.2	Digestive	0.020	0.030	0.33	0.91	0.050	0.192	0.80	0.97	-0.146	0.206	0.48	0.94				
Esophagitis, GERD and related diseases	530.1	Digestive	0.020	0.010	0.06	0.51	0.063	0.112	0.57	0.92	0.081	0.080	0.31	0.93	-0.053	0.196	0.79	0.99
Femoral hernia	550.3	Digestive	0.000	0.030	0.78	0.98												
Functional digestive disorders	564	Digestive	0.000	0.040	0.20	0.76												
GERD	530.11	Digestive	0.010	0.010	0.20	0.76	0.080	0.084	0.34	0.92	0.075	0.074	0.31	0.93	0.010	0.171	0.96	0.99
Gastric ulcer	531.2	Digestive	0.000	0.020	0.82	0.98	-0.108	0.130	0.41	0.92	-0.263	0.136	0.05	0.69	-0.449	0.261	0.09	0.70
Gastritis and duodenitis	535	Digestive	0.010	0.000	0.05	0.44	-0.016	0.060	0.79	0.97	0.070	0.050	0.16	0.89	-0.192	0.135	0.16	0.73
Gastroesophageal laceration-hemorrhage syndrome	530.7	Digestive	0.000	0.060	0.74	0.97												
Gastrointestinal complications	569.2	Digestive	-0.100	0.070	0.08	0.59												
Gastrojejunal ulcer	531.5	Digestive	-0.400	0.280	0.08	0.57												
Gingival and periodontal diseases	523	Digestive	0.000	0.050	0.75	0.97	-0.227	0.226	0.31	0.92								
Gingivitis	523.1	Digestive	0.000	0.130	0.50	0.95												
Glossitis	529.1	Digestive	0.000	0.060	0.97	1.00												
Heartburn	530.9	Digestive	0.050	0.020	0.08	0.57	-0.159	0.197	0.42	0.92	-0.211	0.167	0.21	0.89				
Hematemesis	578.1	Digestive	0.000	0.020	0.17	0.72	0.086	0.193	0.65	0.93	0.224	0.168	0.18	0.89				
Hemorrhage from gastrointestinal ulcer	531.1	Digestive	0.030	0.040	0.34	0.92												
Hemorrhage of gastrointestinal tract	578.9	Digestive	0.000	0.010	0.81	0.98	-0.049	0.084	0.56	0.92	-0.098	0.082	0.23	0.89	0.008	0.160	0.96	0.99
Hemorrhage of rectum and anus	578.8	Digestive	0.000	0.010	0.99	1.00	0.055	0.062	0.37	0.92	-0.029	0.064	0.65	0.95	-0.039	0.145	0.79	0.99
Hepatomegaly	573.3	Digestive	0.000	0.200	0.98	1.00												
Hereditary disturbances in tooth structure	520.1	Digestive	-0.100	0.210	0.48	0.95												
Hypertrophy of salivary gland	527.1	Digestive	-0.100	0.160	0.32	0.89												
Ileostomy status	559	Digestive	0.040	0.030	0.13	0.68												
Impaction of intestine	560.2	Digestive	0.000	0.070	0.89	1.00												
Inflammatory conditions of jaw	526.5	Digestive	-0.100	0.100	0.14	0.68												
Inguinal hernia	550.1	Digestive	0.000	0.000	0.03	0.31	-0.023	0.077	0.76	0.97	-0.163	0.063	0.01	0.31	-0.437	0.332	0.19	0.73
Intestinal malabsorption (non-celiac)	557	Digestive	0.000	0.090	0.95	1.00												
Irritable Bowel Syndrome	564.1	Digestive	0.000	0.020	0.60	0.97					0.121	0.192	0.53	0.94				
Jaundice (not of newborn)	573.5	Digestive	0.000	0.040	0.90	1.00												
Jaw disease NOS	526.9	Digestive	-0.100	0.080	0.04	0.38												
Leukoplakia of oral mucosa	528.6	Digestive	0.000	0.050	0.98	1.00												
Liver abscess and sequelae of chronic liver disease	571.8	Digestive	0.070	0.040	0.13	0.67												
Noninfectious gastroenteritis	558	Digestive	0.000	0.010	0.90	1.00	-0.036	0.083	0.66	0.93	-0.096	0.064	0.13	0.89	-0.315	0.208	0.13	0.71
Nonspecific abnormal findings in stool contents	579.8	Digestive	0.000	0.020	0.18	0.75	-0.166	0.181	0.36	0.92	0.071	0.137	0.60	0.94				
Nonspecific abnormal findings on radiological and oth	575.9	Digestive	0.000	0.080	0.77	0.97												
Obstruction of bile duct	575.2	Digestive	0.000	0.030	0.99	1.00												
Oral aphthae	528.12	Digestive	0.290	0.170	0.10	0.62												
Other chronic nonalcoholic liver disease	571.5	Digestive	0.020	0.050	0.70	0.97												
Other diseases of the teeth and supporting structures	525	Digestive	0.000	0.020	0.80	0.98	0.109	0.096	0.26	0.92	-0.194	0.135	0.15	0.89				
Other disorders of biliary tract	575.8	Digestive	0.010	0.040	0.67	0.97												
Other disorders of gallbladder	575.7	Digestive	0.000	0.050	0.96	1.00												
Other disorders of intestine	569	Digestive	0.000	0.020	0.18	0.75	-0.164	0.138	0.24	0.91	-0.209	0.154	0.17	0.89				
Other disorders of liver	573	Digestive	0.000	0.040	0.78	0.98	0.216	0.206	0.29	0.92								
Other disorders of peritoneum	568	Digestive	0.000	0.100	0.81	0.98												
Other disorders of stomach and duodenum	537	Digestive	0.020	0.020	0.35	0.93	0.102	0.153	0.51	0.92	0.197	0.147	0.18	0.89				
Other intestinal obstruction	560.4	Digestive	0.000	0.010	0.30	0.87	-0.086	0.116	0.46	0.92	0.039	0.147	0.79	0.98				
Other specified diseases of the salivary glands	527.8	Digestive	-0.100	0.100	0.24	0.80												
Other specified gastritis	535.8	Digestive	0.000	0.010	0.89	1.00	0.063	0.075	0.40	0.92	-0.032	0.069	0.64	0.95	-0.084	0.154	0.58	0.92
Other symptoms involving abdomen and pelvis	579	Digestive	0.000	0.030	0.98	1.00												
Paralytic ileus	560.1	Digestive	0.000	0.090	0.57	0.97												
Peptic ulcer, site unspecified	531.4	Digestive	0.090	0.090	0.34	0.91												
Periapical abscess	522.5	Digestive	0.000	0.030	0.52	0.95	0.183	0.163	0.26	0.92	0.071	0.201	0.72	0.97				
Peritoneal adhesions (postoperative) (postinfection)	568.1	Digestive	0.000	0.040	0.95	1.00												
Peritoneal or intestinal adhesions	560.3	Digestive	0.000	0.030	0.77	0.97	-0.241	0.183	0.19	0.90								
Peritonitis and retroperitoneal infections	567	Digestive	0.000	0.040	0.36	0.93												
Personal history of diseases of digestive system	564.9	Digestive	0.000	0.080	0.39	0.94												
Portal hypertension	571.81	Digestive	0.130	0.090	0.16	0.72												
Primary biliary cirrhosis	571.6	Digestive	0.000	0.090	0.96	1.00												
Pulpitis and necrosis of tooth pulp	522.1	Digestive	-0.100	0.150	0.49	0.95												
Reflux esophagitis	530.14	Digestive	0.000	0.010	0.86	0.99	0.234	0.092	0.01	0.34	-0.052	0.070	0.45	0.94	0.245	0.211	0.25	0.84
Regional enteritis	555.1	Digestive	0.000	0.020	0.39	0.94	-0.232	0.239	0.33	0.92	0.189	0.162	0.24	0.91				
Sialoadenitis	527.2	Digestive	0.000	0.050	0.43	0.95												
Sialolithiasis	528.7	Digestive	0.000	0.060	0.41	0.95												
Splenomegaly	579.2	Digestive	-0.200	0.170	0.08	0.59												
Stomatitis and mucositis (ulcerative)	528.11	Digestive	0.000	0.070	0.78	0.98												
Stricture and stenosis of esophagus	530.3	Digestive	0.010	0.020	0.51	0.95	-0.155	0.240	0.52	0.92	-0.284	0.201	0.16	0.89				
Symptoms involving digestive system	561	Digestive	0.000	0.000	0.46	0.95	-0.004	0.073	0.95	0.99	-0.089	0.063	0.15	0.89	0.128	0.122	0.29	0.84
Temporomandibular joint disorder, unspecified	526.41	Digestive	0.000	0.070	0.35	0.92												
Toxic gastroenteritis and colitis	569.1	Digestive	0.020	0.060	0.72	0.97												
Ulcer of esophagus	530.12	Digestive	0.000	0.010	0.07	0.57	0.112	0.230	0.63	0.92	0.077	0.134	0.57	0.94				
Ulceration of intestine	556.1	Digestive	0.040	0.050	0.35	0.92					-0.220	0.236	0.35	0.94				
Ulceration of the lower GI tract	556	Digestive	0.000	0.060	0.63	0.97												
Ulcerative colitis	555.2	Digestive	0.020	0.020	0.28	0.86	-0.024	0.181	0.89	0.98	-0.050	0.110	0.65	0.95				
Ulcerative colitis (chronic)	555.21	Digestive	0.050	0.040	0.20	0.76												
Umbilical hernia	550.4	Digestive	0.000	0.010	0.65	0.97	-0.295	0.108	0.01	0.30	-0.151	0.119	0.21	0.89				
Ventral hernia	550.5	Digestive	0.020	0.010	0.19	0.76	-0.082	0.115	0.48	0.92	0.106	0.127	0.40	0.94				

Acidosis	276.41	Endocrine/Metabolic	0.070	0.130	0.60	0.97												
Acromegaly and gigantism	253.11	Endocrine/Metabolic	-0.300	0.150	0.01	0.19												
Alanine aminotransferace		Endocrine/Metabolic	0.011	0.002	1.05E-11	7.69E-10	0.003	0.011	0.79	0.97	0.007	0.010	0.49	0.94	0.003	0.020	0.87	0.99
Albumin		Endocrine/Metabolic	-0.041	0.005	1.62E-18	1.69E-16	-0.001	0.033	0.97	1.00	0.012	0.031	0.71	0.97	0.032	0.059	0.59	0.92
Alkaline phosphatase		Endocrine/Metabolic	-0.007	0.002	1.37E-04	3.99E-03	-0.010	0.011	0.37	0.92	0.005	0.011	0.63	0.94	-0.032	0.021	0.13	0.71
Amyloidosis	270.33	Endocrine/Metabolic	0.050	0.100	0.60	0.97												
Anorexia	260.6	Endocrine/Metabolic	0.000	0.100	0.57	0.97												
Apolipoprotein A		Endocrine/Metabolic	-0.001	0.000	0.00	0.03	-0.007	0.003	0.02	0.41	4.87E-04	0.003	0.85	0.98	-0.001	0.006	0.92	0.99
Apolipoprotein B		Endocrine/Metabolic	0.004	0.000	1.09E-26	1.59E-24	0.030	0.003	7.08E-28	1.11E-25	0.005	0.003	0.08	0.82	0.006	0.005	0.25	0.84
Asparate aminotransferase		Endocrine/Metabolic	0.006	0.002	1.21E-04	3.60E-03	-0.006	0.011	0.60	0.92	0.015	0.011	0.15	0.89	-0.001	0.021	0.96	0.99
Bioavailable Testosterone, Females		Endocrine/Metabolic	0.006	0.003	0.04	0.42	0.008	0.017	0.66	0.93	0.024	0.019	0.21	0.89	-0.025	0.030	0.39	0.88
Bioavailable Testosterone, Males		Endocrine/Metabolic	-0.001	0.003	0.70	0.97	-0.006	0.017	0.72	0.96	0.002	0.015	0.87	0.98	0.040	0.036	0.27	0.84
C-reactive protein		Endocrine/Metabolic	-0.007	0.002	5.594E-05	1.737E-03	-0.014	0.011	0.23	0.91	-0.014	0.011	0.22	0.89	-0.021	0.021	0.32	0.86
Calcium		Endocrine/Metabolic	-0.001	0.000	3.43E-11	2.38E-09	1.39E-04	0.001	0.91	0.98	0.002	0.001	0.17	0.89	-0.001	0.002	0.57	0.92
Cholesterol		Endocrine/Metabolic	0.049	0.002	1.41E-130	1.03E-127	0.146	0.014	1.89E-24	2.23E-22	0.044	0.014	0.00	0.07	0.042	0.024	0.08	0.70
Chondrocalcinosis	274.21	Endocrine/Metabolic	0.000	0.060	0.92	1.00												
Chronic lymphocytic thyroiditis	245.21	Endocrine/Metabolic	-0.100	0.150	0.44	0.95												
Creatinine		Endocrine/Metabolic	0.002	0.001	0.11	0.64	0.006	0.009	0.53	0.92	-0.004	0.009	0.68	0.96	-0.014	0.016	0.38	0.88
Crystal arthropathies	274.2	Endocrine/Metabolic	0.040	0.100	0.67	0.97												
Cushing's syndrome	255.11	Endocrine/Metabolic	0.000	0.120	0.96	1.00												
Deficiency of humoral immunity	279.11	Endocrine/Metabolic	0.000	0.100	0.48	0.95												
Diabetes insipidus	253.3	Endocrine/Metabolic	0.000	0.200	0.97	1.00												
Diabetic retinopathy	250.7	Endocrine/Metabolic	0.100	0.050	0.04	0.41												
Diastolic blood pressure		Endocrine/Metabolic	0.064	0.020	0.00	0.03	0.233	0.141	0.10	0.66	0.138	0.125	0.27	0.93	-0.216	0.252	0.39	0.88
Direct bilirubin		Endocrine/Metabolic	-0.003	0.002	0.11	0.65	-0.023	0.012	0.07	0.62	0.005	0.012	0.69	0.96	-0.003	0.024	0.90	0.99
Disorders involving the immune mechanism	279	Endocrine/Metabolic	-0.300	0.260	0.22	0.78												
Disorders of adrenal glands	255	Endocrine/Metabolic	0.000	0.110	0.96	1.00												
Disorders of calcium/phosphorus metabolism	275.5	Endocrine/Metabolic	0.050	0.040	0.18	0.75												
Disorders of magnesium metabolism	275.3	Endocrine/Metabolic	0.000	0.070	0.98	1.00												
Disorders of phosphorus metabolism	275.53	Endocrine/Metabolic	0.070	0.140	0.62	0.97												
Disorders of the pituitary gland and its hypothalamic c	253	Endocrine/Metabolic	0.110	0.110	0.33	0.91												
Electrolyte imbalance	276.1	Endocrine/Metabolic	0.200	0.120	0.10	0.62												
Exophthalmos	242.3	Endocrine/Metabolic	0.120	0.180	0.49	0.95												
Fasting glucose		Endocrine/Metabolic	0.006	0.005	0.21	0.77	0.002	0.019	0.93	0.98	0.062	0.029	0.03	0.56				
Fluid overload	276.6	Endocrine/Metabolic	0.090	0.070	0.20	0.77												
Free Testosterone, Females		Endocrine/Metabolic	0.008	0.003	0.01	0.09	0.007	0.017	0.67	0.93	0.026	0.019	0.18	0.89	-0.028	0.030	0.35	0.88
Free Testosterone, Males		Endocrine/Metabolic	0.001	0.003	0.62	0.97	-0.009	0.018	0.61	0.92	0.000	0.015	0.98	1.00	0.029	0.037	0.43	0.90
Gamma glutamyltransferase		Endocrine/Metabolic	0.000	0.002	0.99	1.00	-8.11E-05	0.011	0.99	1.00	-0.005	0.011	0.61	0.94	0.026	0.020	0.19	0.73
Glucocorticoid deficiency	255.21	Endocrine/Metabolic	0.080	0.050	0.13	0.67												
Glycated hemoglobin (HbA1c)		Endocrine/Metabolic	0.032	0.006	6.10E-07	2.47E-05	-0.035	0.066	0.60	0.92	0.052	0.052	0.31	0.93	-0.129	0.087	0.14	0.71
Gout	274.1	Endocrine/Metabolic	0.000	0.040	0.40	0.94												
Gouty arthropathy	274.11	Endocrine/Metabolic	0.070	0.100	0.47	0.95												
Graves' disease	242.1	Endocrine/Metabolic	0.020	0.070	0.71	0.97												
HDL-cholesterol		Endocrine/Metabolic	-0.001	0.001	0.16	0.72	-0.021	0.011	0.06	0.62	-0.001	0.011	0.93	0.98	0.014	0.020	0.50	0.90
Hyperaldosteronism	255.12	Endocrine/Metabolic	-0.200	0.170	0.16	0.72												
Hypercholesterolemia	272.11	Endocrine/Metabolic	0.000	0.190	0.99	1.00												
Hyperosmolality and/or hypernatremia	276.11	Endocrine/Metabolic	0.000	0.180	0.93	1.00												
Hyperparathyroidism	252.1	Endocrine/Metabolic	0.000	0.040	0.87	0.99												
Hyperpotassemia	276.13	Endocrine/Metabolic	0.070	0.040	0.07	0.55					0.069	0.185	0.71	0.97				
Hypoglycemia	251.1	Endocrine/Metabolic	0.000	0.040	0.69	0.97	-0.178	0.200	0.37	0.92	-0.023	0.176	0.90	0.98				
Hypopotassemia	276.14	Endocrine/Metabolic	0.000	0.060	0.46	0.95												
Hyposmolality and/or hyponatremia	276.12	Endocrine/Metabolic	0.050	0.030	0.13	0.67					0.065	0.158	0.68	0.96				
Hypothyroidism NOS	244.4	Endocrine/Metabolic	0.020	0.070	0.73	0.97												
Hypovolemia	276.5	Endocrine/Metabolic	0.000	0.040	0.40	0.94												
Iatrogenic endocrine disorders	258	Endocrine/Metabolic	-0.100	0.150	0.29	0.86												
Immunity deficiency	279.1	Endocrine/Metabolic	0.000	0.130	0.96	1.00												
Impaired fasting glucose	250.41	Endocrine/Metabolic	0.210	0.120	0.08	0.57												
Insulin-like growth factor 1		Endocrine/Metabolic	-0.050	0.009	1.12E-07	5.29E-06	-0.003	0.011	0.80	0.97	-0.006	0.011	0.56	0.94	-0.042	0.020	0.04	0.70
LDL-cholesterol		Endocrine/Metabolic	0.049	0.002	4.57E-175	6.66E-172	0.132	0.011	4.90E-32	2.31E-29	0.042	0.011	0.00	0.03	0.028	0.021	0.18	0.73
Localized adiposity	278.3	Endocrine/Metabolic	0.130	0.130	0.31	0.88												
Macroglobulinemia	270.35	Endocrine/Metabolic	0.000	0.120	0.48	0.95												
Mineral deficiency NEC	262	Endocrine/Metabolic	0.100	0.060	0.13	0.67												
Non-HDL cholesterol		Endocrine/Metabolic	0.050	0.002	2.59E-120	9.43E-118	0.130	0.012	4.19E-29	9.87E-27	0.037	0.011	0.00	0.07	0.022	0.022	0.31	0.84
Nonspecific abnormal results of other endocrine functi	259.1	Endocrine/Metabolic	0.000	0.170	0.86	0.99												
Nontoxic multinodular goiter	241.2	Endocrine/Metabolic	0.000	0.040	0.89	1.00	0.239	0.164	0.14	0.83	0.142	0.211	0.50	0.94				
Nontoxic uninodular goiter	241.1	Endocrine/Metabolic	0.040	0.040	0.31	0.89												
Obesity	278.1	Endocrine/Metabolic	0.060	0.040	0.09	0.61	0.291	0.140	0.04	0.56								
Oestradiol		Endocrine/Metabolic	0.003	0.004	0.42	0.95	0.036	0.019	0.06	0.62	0.019	0.021	0.37	0.94	-0.017	0.043	0.69	0.99
Oestradiol, Females		Endocrine/Metabolic	0.004	0.005	0.46	0.95	0.055	0.025	0.03	0.49	0.030	0.030	0.31	0.93	-0.006	0.049	0.91	0.99
Oestradiol, Males		Endocrine/Metabolic	0.005	0.009	0.58	0.97	0.015	0.046	0.74	0.97	-0.019	0.048	0.69	0.96				
Other abnormal glucose	250.42	Endocrine/Metabolic	0.070	0.070	0.36	0.93												
Other disorders of lipoid metabolism	277.5	Endocrine/Metabolic	0.110	0.160	0.48	0.95												
Other disorders of metabolism	277	Endocrine/Metabolic	0.030	0.080	0.72	0.97												
Other disorders of neurohypophysis	253.7	Endocrine/Metabolic	0.100	0.090	0.27	0.84												
Other disorders of thyroid	246	Endocrine/Metabolic	0.240	0.080	0.00	0.05												
Other endocrine disorders	259	Endocrine/Metabolic	0.000	0.120	0.78	0.98												
Other immunological findings	279.7	Endocrine/Metabolic	0.130	0.070	0.07	0.57												
Ovarian dysfunction	256	Endocrine/Metabolic	0.000	0.170	0.77	0.97												
Paraproteinemia	270.32	Endocrine/Metabolic	-0.200	0.090	0.01	0.10												
Phosphate		Endocrine/Metabolic	-0.001	0.000	5.24E-07	2.19E-05	0.002	0.002	0.42	0.92	-0.001	0.002	0.57	0.94	-0.002	0.004	0.52	0.91
Pituitary hyperfunction	253.1	Endocrine/Metabolic	-0.100	0.220	0.52	0.95												
Pituitary hypofunction	253.2	Endocrine/Metabolic	-0.100	0.080	0.17	0.72												
Polycystic ovaries	256.4	Endocrine/Metabolic	-0.300	0.240	0.20	0.76												
Polydipsia	276.8	Endocrine/Metabolic	0.000	0.160	0.85	0.99												
Protein-calorie malnutrition	260	Endocrine/Metabolic	-0.400	0.250	0.07	0.57												
Proteinuria	269	Endocrine/Metabolic	0.000	0.090	0.98	1.00												
Pulse pressure		Endocrine/Metabolic	0.005	0.002	0.00	0.03	0.019	0.011	0.07	0.62	0.010	0.010	0.32	0.94	-0.004	0.019	0.85	0.99
Random glucose		Endocrine/Metabolic	0.000	0.002	0.83	0.98	-0.018	0.013	0.18	0.90	-0.010	0.013	0.45	0.94	0.007	0.024	0.77	0.99
Rheumatoid factor		Endocrine/Metabolic	0.004	0.006	0.51	0.95	-0.024	0.043	0.58	0.92	0.037	0.038	0.32	0.94				
Sex hormone binding globulin		Endocrine/Metabolic	-0.010	0.002	7.54E-09	4.07E-07	-0.010	0.011	0.39	0.92	-0.008	0.011	0.49	0.94	0.014	0.020	0.50	0.90
Sex hormone binding globulin (female)		Endocrine/Metabolic	-0.008	0.003	2.52E-03	0.05	-0.019	0.016	0.23	0.91	-0.008	0.017	0.64	0.95	0.037	0.027	0.17	0.73
Sex hormone binding globulin (male)		Endocrine/Metabolic	-0.016	0.003	1.69E-09	9.50E-08	0.003	0.018	0.89	0.98	0.001	0.015	0.96	0.98	-0.024	0.037	0.52	0.91
Simple and unspecified goiter	240	Endocrine/Metabolic	0.080	0.040	0.09	0.60	-0.364	0.231	0.11	0.72								
Systolic blood pressure		Endocrine/Metabolic	0.137	0.034	6.14E-05	1.86E-03	0.496	0.231	0.03	0.51	0.211	0.215	0.33	0.94	-0.346	0.420	0.41	0.88
Testicular hypofunction	257.1	Endocrine/Metabolic	0.180	0.100	0.06	0.53												
Thyroiditis	245	Endocrine/Metabolic	0.070	0.200	0.72	0.97												
Thyrotoxicosis with or without goiter	242	Endocrine/Metabolic	0.000	0.050	0.76	0.97												
Total bilirubin		Endocrine/Metabolic	-0.004	0.002	0.02	0.24	-0.011	0.011	0.33	0.92	0.003	0.011	0.77	0.98	-0.015	0.021	0.48	0.90
Total protein		Endocrine/Metabolic	0.001	0.007	0.91	1.00	0.067	0.055	0.22	0.91	0.080	0.051	0.12	0.89	-0.012	0.097	0.90	0.99
Toxic multinodular goiter	242.2	Endocrine/Metabolic	-0.100	0.140	0.37	0.94												
Triglycerides		Endocrine/Metabolic	-0.012	0.002	3.16E-13	2.56E-11	-0.010	0.011	0.38	0.92	-0.009	0.011	0.42	0.94	0.002	0.021	0.91	0.99
Triglycerides/HDL-C		Endocrine/Metabolic	-0.007	0.002	4.6E-05	1.49E-03	0.001	0.011	0.90	0.98	-0.010	0.011	0.36	0.94	-0.001	0.021	0.97	0.99
Type 1 diabetes	250.1	Endocrine/Metabolic	0.000	0.050	1.00	1.00												
Type 1 diabetes with ketoacidosis	250.11	Endocrine/Metabolic	-0.100	0.070	0.15	0.69												
Type 1 diabetes with ophthalmic manifestations	250.13	Endocrine/Metabolic	0.080	0.070	0.25	0.81												
Type 2 diabetes	250.2	Endocrine/Metabolic	0.100	0.030	0.00	0.06	0.027	0.100	0.79	0.97	0.125	0.136	0.36	0.94				
Type 2 diabetes		Endocrine/Metabolic	0.037	0.008	1.09E-05	3.97E-04	0.011	0.039	0.78	0.97	-0.003	0.031	0.91	0.98				
Type 2 diabetes with ketoacidosis	250.21	Endocrine/Metabolic	0.000	0.090	0.95	1.00	0.299	0.179	0.09	0.66								
Type 2 diabetes with neurological manifestations	250.24	Endocrine/Metabolic	-0.100	0.140	0.21	0.78												
Type 2 diabetes with ophthalmic manifestations	250.23	Endocrine/Metabolic	0.050	0.050	0.30	0.88	0.371	0.224	0.10	0.66								
Type 2 diabetes with renal manifestations	250.22	Endocrine/Metabolic	-0.100	0.210	0.57	0.97												
Urate		Endocrine/Metabolic	0.274	0.119	0.02	0.26	1.126	0.804	0.16	0.86	1.799	0.787	0.02	0.43	1.603	1.431	0.26	0.84
Urea		Endocrine/Metabolic	-0.005	0.002	0.00	0.07	0.006	0.011	0.60	0.92	0.018	0.011	0.09	0.82	-0.008	0.021	0.70	0.99
Vitamin B-complex deficiencies	261.2	Endocrine/Metabolic	0.000	0.100	0.74	0.97												
Vitamin D		Endocrine/Metabolic	-0.167	0.037	6.55E-06	2.45E-04	-0.027	0.011	0.02	0.42	0.001	0.011	0.93	0.98	0.002	0.022	0.93	0.99
Vitamin D deficiency	261.4	Endocrine/Metabolic	0.070	0.140	0.60	0.97												
Abnormal findings on examination of urine	598	Genitourinary	0.000	0.020	0.22	0.78	-0.029	0.123	0.81	0.97	-0.053	0.108	0.63	0.94				
Abnormal mammogram	611.1	Genitourinary	0.090	0.140	0.50	0.95												
Abnormal results of function study of kidney	589	Genitourinary	-0.100	0.130	0.45	0.95												
Abnormal spermatozoa	609.2	Genitourinary	0.000	0.100	0.93	1.00												
Abscess or ulceration of vulva	614.54	Genitourinary	0.000	0.070	0.33	0.91												
Absent or infrequent menstruation	626.11	Genitourinary	-0.100	0.140	0.33	0.91												
Acute cystitis	592.11	Genitourinary	0.000	0.080	0.94	1.00												

Acute inflammatory pelvic disease	614.31	Genitourinary	0.010	0.190	0.96	1.00												
Acute prostatitis	601.11	Genitourinary	-0.100	0.070	0.16	0.71												
Acute renal failure	585.1	Genitourinary	0.040	0.020	0.03	0.31	0.095	0.116	0.41	0.92	-0.049	0.119	0.68	0.96				
Albuminuria		Genitourinary	-0.006	0.008	0.46	0.95	0.071	0.043	0.10	0.66	-0.007	0.037	0.86	0.98				
Atrophy of female genital tract	624.2	Genitourinary	-0.200	0.160	0.10	0.62												
Balanoposthitis	601.4	Genitourinary	-0.100	0.070	0.08	0.59												
Benign neoplasm of breast	610.4	Genitourinary	0.010	0.020	0.52	0.95	0.070	0.130	0.59	0.92	-0.165	0.175	0.34	0.94				
Bladder neck obstruction	596.1	Genitourinary	0.010	0.020	0.65	0.97	-0.130	0.200	0.52	0.92	0.048	0.149	0.75	0.98				
Breast disorder NOS	613.9	Genitourinary	0.090	0.070	0.20	0.77												
Calculus of kidney	594.1	Genitourinary	0.000	0.010	0.86	0.99	-0.032	0.159	0.84	0.98	-0.111	0.111	0.32	0.93				
Calculus of lower urinary tract	594.2	Genitourinary	0.000	0.030	0.29	0.86												
Calculus of ureter	594.3	Genitourinary	0.010	0.020	0.53	0.95	0.044	0.172	0.80	0.97	0.002	0.119	0.99	1.00				
Cervicitis and endocervicitis	614.51	Genitourinary	0.020	0.050	0.68	0.97												
Chronic cystitis	592.12	Genitourinary	0.020	0.040	0.59	0.97												
Chronic glomerulonephritis, NOS	580.14	Genitourinary	-0.300	0.220	0.18	0.74												
Chronic inflammatory pelvic disease	614.32	Genitourinary	0.030	0.080	0.64	0.97												
Chronic interstitial cystitis	592.13	Genitourinary	0.000	0.060	0.46	0.95												
Chronic kidney disease		Genitourinary	-0.004	0.014	0.78	0.98												
Chronic prostatitis	601.12	Genitourinary	0.000	0.040	0.67	0.97												
Chronic renal failure [CKD]	585.3	Genitourinary	0.020	0.030	0.40	0.94	-0.105	0.140	0.45	0.92	-0.075	0.134	0.58	0.94				
Congenital anomalies of breast	612.3	Genitourinary	0.040	0.120	0.71	0.97												
Cyst of kidney, acquired	586.2	Genitourinary	0.080	0.070	0.27	0.84												
Cyst or abscess of Bartholin's gland	614.53	Genitourinary	0.000	0.040	0.13	0.67	-0.091	0.205	0.66	0.93								
Cystic mastopathy	610.1	Genitourinary	0.000	0.040	0.53	0.95												
Cystitis	592.1	Genitourinary	0.000	0.020	0.90	1.00	-0.121	0.206	0.56	0.92	-0.110	0.210	0.60	0.94				
Disorders of menstruation and other abnormal bleedin	626	Genitourinary	0.000	0.030	0.55	0.96	0.028	0.161	0.86	0.98	0.201	0.147	0.17	0.89				
Disorders of penis	604	Genitourinary	0.060	0.030	0.08	0.57												
Disorders of uterus, NEC	619.2	Genitourinary	0.000	0.030	0.89	1.00	-0.018	0.206	0.93	0.98								
Disorders resulting from impaired renal function	588	Genitourinary	0.050	0.180	0.76	0.97												
Dysmenorrhea	626.2	Genitourinary	-0.100	0.060	0.09	0.61												
Dyspareunia	625.1	Genitourinary	0.000	0.050	0.08	0.57												
Dysplasia of female genital organs	620	Genitourinary	0.030	0.120	0.79	0.98												
Dystrophy of female genital tract	624.1	Genitourinary	0.000	0.060	0.93	1.00												
Dysuria	599.3	Genitourinary	0.000	0.040	0.99	1.00												
Endometrial hyperplasia	621	Genitourinary	0.000	0.040	0.56	0.97												
Endometriosis	615	Genitourinary	0.020	0.020	0.32	0.89	0.006	0.165	0.97	1.00	-0.038	0.157	0.81	0.98				
Erectile dysfunction [ED]	605	Genitourinary	0.000	0.050	0.63	0.97	0.036	0.234	0.88	0.98								
Estimated glomerular filtration rate (creatinine and cystatin C)		Genitourinary	0.011	0.022	0.63	0.97	-0.010	0.010	0.31	0.92	-0.003	0.009	0.76	0.98	0.025	0.017	0.15	0.72
Excessive or frequent menstruation	626.12	Genitourinary	0.020	0.010	0.07	0.57	-0.001	0.068	0.99	1.00	0.090	0.082	0.27	0.93	0.278	0.156	0.07	0.70
Fibroadenosis of breast	610.2	Genitourinary	0.070	0.080	0.37	0.94												
Fibrosclerosis of breast	610.3	Genitourinary	0.070	0.070	0.33	0.91												
Frequency of urination and polyuria	599.5	Genitourinary	0.030	0.020	0.08	0.57	-0.057	0.149	0.70	0.94	-0.085	0.137	0.54	0.94				
Functional disorders of bladder	596.5	Genitourinary	0.010	0.030	0.64	0.97					0.361	0.203	0.07	0.82				
Genital prolapse	618	Genitourinary	0.000	0.090	0.51	0.95												
Hematuria	593	Genitourinary	0.000	0.000	0.20	0.77	0.019	0.060	0.75	0.97	0.084	0.062	0.17	0.89	-0.062	0.123	0.61	0.93
Hydrocele	603.1	Genitourinary	0.000	0.030	0.38	0.94	-0.179	0.178	0.31	0.92	0.087	0.211	0.68	0.96				
Hydronephrosis	595	Genitourinary	0.000	0.020	0.51	0.95	0.019	0.178	0.91	0.98	-0.137	0.167	0.41	0.94				
Hyperplasia of prostate	600	Genitourinary	0.000	0.010	0.80	0.98	0.070	0.089	0.43	0.92	-0.010	0.079	0.90	0.98				
Hypertrophy of breast (Gynecomastia)	612.2	Genitourinary	0.000	0.040	0.15	0.69												
Hypertrophy of female genital organs	623	Genitourinary	0.000	0.060	0.75	0.97												
Infertility, female	626.8	Genitourinary	0.000	0.030	0.93	1.00	-0.111	0.181	0.54	0.92	0.184	0.184	0.32	0.93				
Infertility, male	609.1	Genitourinary	0.000	0.160	1.00	1.00												
Inflammatory disease of breast	613.1	Genitourinary	0.000	0.040	0.58	0.97												
Inflammatory disease of cervix, vagina, and vulva	614.5	Genitourinary	0.000	0.100	0.48	0.95												
Inflammatory diseases of uterus, except cervix	614.4	Genitourinary	0.070	0.080	0.36	0.93												
Irregular menstrual bleeding	626.14	Genitourinary	0.000	0.020	0.42	0.95	-0.276	0.153	0.07	0.62	0.017	0.136	0.90	0.98				
Irregular menstrual cycle	626.13	Genitourinary	0.000	0.030	0.40	0.94	-0.334	0.234	0.15	0.86	-0.245	0.201	0.22	0.89				
Irregular menstrual cycle/bleeding	626.1	Genitourinary	0.000	0.030	0.67	0.97					-0.056	0.202	0.78	0.98				
Lump or mass in breast	611.3	Genitourinary	0.000	0.020	0.61	0.97	0.000	0.153	1.00	1.00	-0.124	0.222	0.58	0.94				
Mastodynia	613.5	Genitourinary	0.000	0.150	0.93	1.00												
Menopausal and postmenopausal disorders	627	Genitourinary	0.000	0.060	0.92	1.00												
Mucous polyp of cervix	622.2	Genitourinary	0.000	0.020	0.02	0.25	0.160	0.164	0.33	0.92	0.084	0.150	0.58	0.94				
Nephritis and nephropathy with pathological lesion	580.32	Genitourinary	0.000	0.100	0.35	0.92												
Nephrotic syndrome without mention of glomerulonep	580.2	Genitourinary	0.000	0.090	0.70	0.97												
Non-proliferative glomerulonephritis	580.12	Genitourinary	0.000	0.100	0.77	0.97												
Noninflammatory disorders of cervix	619.3	Genitourinary	0.000	0.030	0.06	0.54	-0.285	0.199	0.15	0.86								
Noninflammatory disorders of ovary, fallopian tube, ar	619.1	Genitourinary	0.000	0.060	0.93	1.00												
Noninflammatory disorders of vagina	619.4	Genitourinary	0.000	0.030	0.51	0.95												
Noninflammatory disorders of vulva and perineum	619.5	Genitourinary	0.000	0.030	0.60	0.97	0.388	0.205	0.06	0.62	-0.014	0.223	0.95	0.98				
Noninflammatory female genital disorders	619	Genitourinary	0.000	0.090	0.97	1.00												
Other abnormality of urination	599.9	Genitourinary	0.050	0.030	0.11	0.64					0.016	0.222	0.94	0.98				
Other disorders of bladder	596	Genitourinary	0.000	0.010	0.96	1.00	-0.125	0.102	0.22	0.91	0.231	0.097	0.02	0.40				
Other disorders of male genital organs	608	Genitourinary	0.040	0.020	0.07	0.55	-0.167	0.177	0.34	0.92	-0.019	0.173	0.91	0.98				
Other disorders of prostate	602	Genitourinary	0.040	0.030	0.19	0.76												
Other disorders of testis	603	Genitourinary	0.000	0.030	0.53	0.95	0.327	0.202	0.11	0.70	-0.014	0.186	0.94	0.98				
Other disorders of the kidney and ureters	586	Genitourinary	0.000	0.040	0.19	0.76												
Other disorders of urethra and urinary tract	597	Genitourinary	0.000	0.040	0.53	0.95												
Other inflammatory disorders of male genital organs	601.8	Genitourinary	0.000	0.060	0.47	0.95												
Other signs and symptoms in breast	613.7	Genitourinary	0.000	0.050	0.87	0.99												
Other specified benign mammary dysplasias	610.8	Genitourinary	0.000	0.040	0.67	0.97					-0.109	0.227	0.63	0.94				
Other specified disorders of breast	613.8	Genitourinary	0.000	0.060	0.36	0.93												
Other symptoms/disorders or the urinary system	599	Genitourinary	0.000	0.010	0.56	0.96	-0.044	0.108	0.68	0.93	-0.050	0.090	0.58	0.94	-0.161	0.273	0.56	0.92
Ovarian cyst	628	Genitourinary	0.010	0.020	0.43	0.95	0.027	0.134	0.84	0.98	-0.244	0.178	0.17	0.89	0.114	0.217	0.60	0.93
Pain and other symptoms associated with female genit	625	Genitourinary	0.000	0.030	0.42	0.95	0.050	0.197	0.80	0.97	0.074	0.218	0.73	0.98				
Pelvic inflammatory disease (PID)	614.3	Genitourinary	0.020	0.100	0.78	0.98												
Pelvic inflammatory disease, NOS	614.33	Genitourinary	0.000	0.070	0.96	1.00												
Pelvic peritoneal adhesions, female (postoperative) (pc	614.1	Genitourinary	0.010	0.040	0.70	0.97	-0.031	0.180	0.87	0.98								
Polyp of corpus uteri	622.1	Genitourinary	0.000	0.010	0.43	0.95	-0.082	0.082	0.32	0.92	0.048	0.087	0.58	0.94	-0.096	0.171	0.57	0.92
Polyp of female genital organs	622	Genitourinary	0.000	0.080	0.71	0.97												
Postmenopausal atrophic vaginitis	627.3	Genitourinary	0.000	0.060	0.90	1.00												
Postmenopausal bleeding	627.1	Genitourinary	0.010	0.010	0.15	0.69	-0.036	0.089	0.69	0.93	-0.018	0.092	0.84	0.98	-0.004	0.168	0.98	0.99
Premenopausal menorrhagia	627.4	Genitourinary	0.000	0.070	0.39	0.94												
Premenstrual tension syndromes	626.4	Genitourinary	-0.300	0.180	0.10	0.62												
Prolapse of vaginal vault after hysterectomy	618.5	Genitourinary	0.000	0.050	0.85	0.99												
Prolapse of vaginal walls	618.1	Genitourinary	0.000	0.010	0.69	0.97	-0.133	0.145	0.36	0.92	0.204	0.124	0.10	0.86				
Proliferative glomerulonephritis	580.11	Genitourinary	-0.400	0.260	0.09	0.60												
Prostatitis	601.1	Genitourinary	0.000	0.040	0.77	0.97												
Pyelonephritis	590	Genitourinary	0.000	0.020	0.84	0.98	0.099	0.147	0.50	0.92	-0.044	0.124	0.72	0.97				
Redundant prepuce and phimosis/BXO	604.1	Genitourinary	0.000	0.020	0.66	0.97	0.538	0.175	0.00	0.14	-0.108	0.130	0.41	0.94				
Renal colic	594.8	Genitourinary	0.000	0.020	0.37	0.94	-0.183	0.175	0.30	0.92	-0.116	0.148	0.43	0.94				
Renal failure NOS	585.2	Genitourinary	0.000	0.070	0.98	1.00												
Retention of urine	599.2	Genitourinary	0.020	0.010	0.23	0.79	-0.235	0.127	0.07	0.62	0.086	0.113	0.44	0.94				
Small kidney	586.11	Genitourinary	0.020	0.170	0.90	1.00												
Stricture/obstruction of ureter	586.4	Genitourinary	0.000	0.030	0.32	0.89												
Symptomatic menopause	627.2	Genitourinary	0.080	0.100	0.42	0.95												
Urethral discharge	599.7	Genitourinary	0.050	0.210	0.82	0.98												
Urethral stricture (not specified as infectious)	597.1	Genitourinary	0.000	0.010	0.92	1.00	0.124	0.126	0.33	0.92	-0.004	0.094	0.96	0.98				
Urethral syndrome	592.21	Genitourinary	-0.200	0.200	0.24	0.80												
Urethritis and urethral syndrome	592.2	Genitourinary	0.020	0.110	0.80	0.98												
Urinary albumin-to-creatine ratio		Genitourinary	-0.003	0.002	0.04	0.42	0.008	0.011	0.49	0.92	-0.006	0.011	0.57	0.94	-0.014	0.021	0.51	0.90
Urinary calculus	594	Genitourinary	0.050	0.040	0.21	0.77					-0.207	0.233	0.37	0.94				
Urinary complications NEC	597.2	Genitourinary	-0.100	0.110	0.23	0.79												
Urinary incontinence	599.4	Genitourinary	0.000	0.010	0.83	0.98	-0.064	0.127	0.61	0.92	-0.030	0.098	0.76	0.98				
Urinary obstruction	599.1	Genitourinary	0.050	0.090	0.56	0.96												
Urinary potassium-to-creatinine ratio		Genitourinary	-0.004	0.002	0.01	0.12	-0.010	0.011	0.38	0.92	0.006	0.010	0.56	0.94	0.034	0.020	0.09	0.70
Urinary sodium to creatinine ratio		Genitourinary	0.003	0.002	0.10	0.62	-0.016	0.012	0.17	0.86	0.019	0.011	0.08	0.82	-0.020	0.022	0.35	0.88
Urinary sodium-to-potassium ratio		Genitourinary	0.006	0.002	0.00	0.01	-0.008	0.012	0.47	0.92	0.015	0.011	0.17	0.89	-0.041	0.022	0.06	0.70
Urinary tract infection	591	Genitourinary	0.000	0.010	0.82	0.98	0.066	0.077	0.39	0.92	0.094	0.064	0.14	0.89	0.174	0.186	0.35	0.88
Urine albumin		Genitourinary	-0.001	0.001	0.70	0.97	0.010	0.010	0.34	0.92	-0.008	0.010	0.40	0.94	-0.005	0.018	0.77	0.99
Urine creatinine		Genitourinary	0.003	0.002	0.13	0.67	0.001	0.011	0.91	0.98	-0.006	0.011	0.58	0.94	0.001	0.021	0.94	0.99
Urine potassium		Genitourinary	1.14E-04	0.002	0.95	1.00	-0.007	0.012	0.56	0.92	-0.005	0.011	0.68	0.96	0.034	0.022	0.13	0.71
Urine sodium		Genitourinary	0.006	0.002	4.06E-04	0.01	-0.019	0.011	0.09	0.66	0.010	0.011	0.39	0.94	-0.017	0.021	0.42	0.90
Uterine/Uterovaginal prolapse	618.2	Genitourinary	0.00E+00	0.010	0.99	1.00	-0.119	0.186	0.52	0.92	-0.007	0.121	0.96	0.98				

Vaginal enterocele, congenital or acquired	618.6	Genitourinary	0.010	0.060	0.81	0.98												
Vaginitis and vulvovaginitis	614.52	Genitourinary	0.080	0.060	0.15	0.70												
Vascular disorders of kidney/hypertrophy	586.3	Genitourinary	0.060	0.180	0.75	0.97												
stress incontinence, female	624.9	Genitourinary	0.000	0.010	0.55	0.96	-0.092	0.186	0.62	0.92	-0.019	0.122	0.88	0.98				
Abnormality of red blood cells	289.9	Hematopoietic	0.000	0.160	0.65	0.97												
Allergic purpura	287.2	Hematopoietic	0.000	0.190	0.69	0.97												
Anemia in neoplastic disease	285.22	Hematopoietic	0.110	0.060	0.09	0.60												
Anemia of chronic disease	285.2	Hematopoietic	0.050	0.060	0.39	0.94												
Aplastic anemia	284	Hematopoietic	0.090	0.050	0.10	0.62												
Autoimmune hemolytic anemias	283.1	Hematopoietic	0.030	0.100	0.77	0.97												
Basophil %		Hematopoietic	0.001	0.002	0.70	0.97	0.006	0.012	0.61	0.92	0.015	0.011	0.18	0.89	0.032	0.022	0.15	0.72
Basophil count		Hematopoietic	0.002	0.002	0.24	0.80	-0.002	0.011	0.85	0.98	0.002	0.011	0.86	0.98	0.036	0.021	0.08	0.70
Congenital factor VIII disorder	286.13	Hematopoietic	-0.200	0.240	0.24	0.81												
Diseases of spleen	289.5	Hematopoietic	0.050	0.120	0.68	0.97												
Diseases of white blood cells	288	Hematopoietic	0.000	0.070	0.96	1.00												
Disorders of iron metabolism	275.1	Hematopoietic	0.000	0.030	0.86	0.99												
Eosinophil %		Hematopoietic	-0.001	0.002	0.71	0.97	0.006	0.012	0.65	0.93	0.020	0.011	0.08	0.82	-0.004	0.022	0.85	0.99
Eosinophil count		Hematopoietic	-0.001	0.002	0.47	0.95	-0.001	0.012	0.91	0.98	0.014	0.011	0.21	0.89	0.004	0.022	0.85	0.99
Eosinophilia	288.3	Hematopoietic	0.200	0.150	0.16	0.72												
Folate-deficiency anemia	281.13	Hematopoietic	0.140	0.090	0.12	0.66												
Hematocrit %		Hematopoietic	-0.020	0.005	5.29E-05	1.68E-03	-0.022	0.037	0.56	0.92	-0.062	0.036	0.08	0.82	-0.066	0.063	0.30	0.84
Hemoglobin concentration		Hematopoietic	-0.008	0.002	4.40E-06	1.69E-04	0.002	0.013	0.90	0.98	-0.016	0.013	0.20	0.89	-0.006	0.022	0.79	0.99
Hemorrhagic disorder due to intrinsic circulating antic	286.5	Hematopoietic	0.000	0.190	0.65	0.97												
High light scatter reticulocyte %		Hematopoietic	0.005	0.002	2.60E-03	0.05	0.022	0.012	0.07	0.62	-0.009	0.012	0.43	0.94	0.000	0.022	1.00	1.00
High light scatter reticulocyte count		Hematopoietic	0.004	0.002	0.01	0.17	0.023	0.012	0.06	0.62	-0.012	0.011	0.29	0.93	-0.003	0.022	0.89	0.99
Immature reticulocyte fraction		Hematopoietic	0.000	0.000	0.47	0.95	0.002	0.001	0.01	0.30	-3.50E-04	0.001	0.63	0.94	1.05E-04	0.001	0.94	0.99
Iron deficiency anemia secondary to blood loss (chroni	280.2	Hematopoietic	0.040	0.080	0.63	0.97												
Iron deficiency anemias, unspecified or not due to bloo	280.1	Hematopoietic	0.030	0.010	0.01	0.09	-0.134	0.081	0.10	0.66	-0.007	0.056	0.90	0.98	0.146	0.205	0.47	0.90
Lymphadenitis	289.4	Hematopoietic	0.000	0.020	0.87	0.99	-0.041	0.138	0.77	0.97	0.028	0.122	0.82	0.98				
Lymphoctye %		Hematopoietic	0.012	0.013	0.34	0.92	0.032	0.107	0.77	0.97	0.121	0.085	0.15	0.89	0.005	0.171	0.98	0.99
Lymphoctye count		Hematopoietic	0.001	0.002	0.75	0.97	-0.007	0.012	0.57	0.92	-0.003	0.011	0.77	0.98	-0.003	0.022	0.88	0.99
Mean corpuscular hemoglobin		Hematopoietic	-0.001	0.002	0.59	0.97	-0.003	0.012	0.79	0.97	0.014	0.011	0.21	0.89	0.037	0.022	0.09	0.70
Mean corpuscular hemoglobin concentration		Hematopoietic	-0.002	0.002	0.23	0.79	0.023	0.012	0.05	0.62	0.009	0.011	0.41	0.94	0.044	0.022	0.04	0.70
Mean reticulocyte volume		Hematopoietic	-0.043	0.013	0.00	0.03	-0.110	0.097	0.25	0.92	0.020	0.094	0.83	0.98	0.060	0.167	0.72	0.99
Mean sphered cell volume		Hematopoietic	-0.014	0.009	0.12	0.66	-0.054	0.073	0.46	0.92	0.008	0.064	0.90	0.98	0.214	0.124	0.08	0.70
Monocyte %		Hematopoietic	0.003	0.002	0.06	0.50	0.021	0.012	0.07	0.62	0.007	0.011	0.50	0.94	0.034	0.022	0.12	0.71
Monocyte count		Hematopoietic	0.001	0.002	0.42	0.95	0.010	0.012	0.39	0.92	-0.007	0.011	0.54	0.94	0.032	0.022	0.13	0.71
Neutropenia	288.11	Hematopoietic	0.000	0.020	0.36	0.93	-0.186	0.204	0.36	0.92	-0.287	0.235	0.22	0.89				
Neutrophil %		Hematopoietic	-0.021	0.014	0.14	0.68	-0.090	0.117	0.44	0.92	-0.167	0.095	0.08	0.82	-0.044	0.191	0.82	0.99
Neutrophil count		Hematopoietic	-0.002	0.002	0.28	0.86	-0.013	0.012	0.28	0.92	-0.028	0.011	0.01	0.33	-0.002	0.022	0.92	0.99
Nucleated red blood cell %		Hematopoietic	0.000	0.000	0.55	0.96	0.005	0.007	0.43	0.92	-0.001	0.003	0.74	0.98	-0.003	0.006	0.64	0.96
Nucleated red blood cell count		Hematopoietic	0.000	0.000	0.49	0.95	0.006	0.007	0.43	0.92	-0.001	0.003	0.80	0.98	-0.003	0.006	0.66	0.97
Other and unspecified coagulation defects	286.7	Hematopoietic	0.000	0.160	0.56	0.97												
Other anemias	285	Hematopoietic	0.020	0.010	0.07	0.56	0.005	0.070	0.94	0.98	0.074	0.059	0.21	0.89	-0.252	0.212	0.23	0.84
Other diseases of blood and blood-forming organs	289	Hematopoietic	0.000	0.050	0.43	0.95												
Other hereditary hemolytic anemias	282.9	Hematopoietic	0.300	0.160	0.05	0.49												
Other vitamin B12 deficiency anemia	281.12	Hematopoietic	-0.100	0.190	0.54	0.96												
Pernicious anemia	281.11	Hematopoietic	0.000	0.150	0.76	0.97												
Platelet count		Hematopoietic	-0.011	0.002	1.16E-10	7.67E-09	-0.026	0.011	0.02	0.46	0.013	0.011	0.25	0.91	0.011	0.022	0.61	0.93
Platelet crit		Hematopoietic	-0.009	0.002	1.81E-08	9.45E-07	-0.030	0.011	0.01	0.30	0.006	0.011	0.58	0.94	0.007	0.021	0.72	0.99
Platelet distribution width		Hematopoietic	-0.003	0.002	0.13	0.67	0.006	0.012	0.59	0.92	-0.019	0.011	0.10	0.86	-0.021	0.022	0.35	0.88
Polycythemia, secondary	289.8	Hematopoietic	0.000	0.060	0.51	0.95												
Primary thrombocytopenia	287.31	Hematopoietic	0.000	0.060	0.60	0.97												
Purpura and other hemorrhagic conditions	287	Hematopoietic	-0.300	0.250	0.21	0.77												
Red blood cell count		Hematopoietic	-0.002	0.001	0.00	0.02	0.000	0.010	0.97	1.00	-0.019	0.010	0.04	0.61	-0.021	0.019	0.26	0.84
Red blood cell distribution width		Hematopoietic	0.003	0.002	0.12	0.67	-0.011	0.012	0.38	0.92	-0.010	0.011	0.40	0.94	0.003	0.022	0.88	0.99
Reticulocyte %		Hematopoietic	0.007	0.002	0.00	0.01	0.010	0.012	0.42	0.92	-0.008	0.012	0.47	0.94	0.002	0.023	0.93	0.99
Reticulocyte count		Hematopoietic	0.005	0.002	0.00	0.06	0.011	0.012	0.38	0.92	-0.014	0.011	0.23	0.89	-0.003	0.022	0.90	0.99
Secondary thrombocytopenia	287.32	Hematopoietic	-0.100	0.210	0.43	0.95												
Sickle cell anemia	282.5	Hematopoietic					0.048	0.199	0.81	0.97								
Spontaneous ecchymoses	287.1	Hematopoietic	0.000	0.140	0.66	0.97												
Thrombocytopenia	287.3	Hematopoietic	0.00E+00	0.050	1.00	1.00												
Von willebrand's disease	286.11	Hematopoietic	-0.100	0.200	0.39	0.94												
White blood cell count		Hematopoietic	-0.001	0.002	0.48	0.95	-0.011	0.012	0.36	0.92	-0.023	0.011	0.04	0.60	0.002	0.022	0.93	0.99
Arthropod-borne diseases	133	Infectious Diseases	-0.100	0.130	0.40	0.94	0.004	0.096	0.97	1.00								
Aspergillosis	117.4	Infectious Diseases	-0.100	0.110	0.14	0.68												
Bacterial enteritis	8.5	Infectious Diseases	0.000	0.030	0.74	0.97	0.122	0.137	0.38	0.92	0.152	0.152	0.31	0.93				
Bacterial infection NOS	41	Infectious Diseases	0.100	0.060	0.09	0.61												
Candidiasis	112	Infectious Diseases	0.000	0.040	0.68	0.97												
Chronic hepatitis	70.4	Infectious Diseases	0.000	0.080	0.28	0.85												
Dermatophytosis of nail	110.11	Infectious Diseases	0.030	0.170	0.83	0.98												
Gram negative septicemia	38.1	Infectious Diseases	0.000	0.030	0.61	0.97	0.068	0.228	0.76	0.97	-0.229	0.207	0.27	0.93				
Gram positive septicemia	38.2	Infectious Diseases	0.000	0.050	0.69	0.97												
HIV infection, symptomatic	71.1	Infectious Diseases	-0.300	0.170	0.03	0.38												
Hepatitis NOS	70.9	Infectious Diseases	0.010	0.050	0.86	0.99												
Herpes simplex	54	Infectious Diseases	0.030	0.140	0.81	0.98												
Herpes zoster	53	Infectious Diseases	0.010	0.070	0.81	0.98												
Herpes zoster with nervous system complications	53.1	Infectious Diseases	0.000	0.120	0.56	0.97												
Infection/inflammation of internal prosthetic device; in	81	Infectious Diseases	0.000	0.030	0.63	0.97	0.116	0.195	0.55	0.92	0.196	0.223	0.38	0.94				
Infectious mononucleosis	79.2	Infectious Diseases	0.240	0.130	0.07	0.57												
Intestinal helminthiases	134.1	Infectious Diseases	0.040	0.140	0.77	0.97												
Intestinal infection	8	Infectious Diseases	0.010	0.010	0.39	0.94	-0.012	0.099	0.91	0.98	0.152	0.079	0.05	0.69	0.163	0.193	0.40	0.88
Intestinal infection due to C. difficile	8.52	Infectious Diseases	0.040	0.050	0.44	0.95												
Intestinal infection due to protozoa	8.7	Infectious Diseases	-0.100	0.180	0.51	0.95												
Lyme disease	130.1	Infectious Diseases	-0.200	0.240	0.30	0.87												
Mycoses	117	Infectious Diseases	-0.300	0.260	0.20	0.76												
Other infectious and parasitic diseases	136	Infectious Diseases	0.170	0.070	0.02	0.22												
Postoperative infection	80	Infectious Diseases	0.000	0.010	0.73	0.97	-0.093	0.119	0.43	0.92	0.128	0.112	0.25	0.92				
Septicemia	38	Infectious Diseases	0.010	0.010	0.21	0.77	-0.009	0.114	0.94	0.98	-0.089	0.103	0.38	0.94				
Spirochetal infection	130	Infectious Diseases	-0.300	0.220	0.18	0.74												
Staphylococcus infections	41.1	Infectious Diseases	0.000	0.100	0.82	0.98												
Streptococcus infection	41.2	Infectious Diseases	0.060	0.090	0.49	0.95												
Tuberculosis	10	Infectious Diseases	0.030	0.090	0.73	0.97	0.001	0.209	1.00	1.00	-0.191	0.148	0.19	0.89				
Varicella infection	79.1	Infectious Diseases	-0.100	0.160	0.51	0.95												
Viral Enteritis	8.6	Infectious Diseases	0.000	0.030	0.94	1.00	0.197	0.191	0.30	0.92	-0.123	0.160	0.44	0.94				
Viral hepatitis	70	Infectious Diseases	-0.100	0.150	0.48	0.95												
Viral hepatitis A	70.1	Infectious Diseases	0.060	0.200	0.77	0.97												
Viral hepatitis B	70.2	Infectious Diseases	0.150	0.160	0.36	0.93												
Viral hepatitis C	70.3	Infectious Diseases	0.000	0.080	0.73	0.97												
Viral infection	79	Infectious Diseases	0.000	0.020	0.72	0.97	-0.081	0.160	0.61	0.92	0.340	0.125	0.01	0.23				
Viral warts & HPV	78	Infectious Diseases	0.000	0.030	0.44	0.95	-0.171	0.245	0.49	0.92								
Adverse drug events and drug allergies	979	Injuries & Poisonings	0.000	0.060	0.73	0.97												
Adverse effects of sedatives or other central nervous sy	967	Injuries & Poisonings	0.000	0.130	0.60	0.97												
Allergic reaction to food	930	Injuries & Poisonings	0.010	0.080	0.88	1.00												
Allergies, other	949	Injuries & Poisonings	0.000	0.050	0.95	1.00												
Anaphylactic shock NOS	946	Injuries & Poisonings	0.000	0.040	0.98	1.00	0.297	0.165	0.07	0.62								
Anticoagulants causing adverse effects	964.1	Injuries & Poisonings	0.290	0.140	0.04	0.40												
Antineoplastic and immunosuppressive drugs causing	963.1	Injuries & Poisonings	0.000	0.210	0.69	0.97												
Certain early complications of trauma or procedure	958	Injuries & Poisonings	0.020	0.060	0.66	0.97												
Complication due to other implant and internal device	859	Injuries & Poisonings	0.000	0.010	0.83	0.98	-0.038	0.120	0.75	0.97	0.029	0.123	0.81	0.98				
Complication of amputation stump	874	Injuries & Poisonings	0.070	0.070	0.35	0.92												
Complication of colostomy or enterostomy	853	Injuries & Poisonings	0.000	0.040	0.54	0.96												
Complication of internal orthopedic device	858	Injuries & Poisonings	0.020	0.010	0.12	0.67	0.002	0.150	0.99	1.00	0.094	0.134	0.48	0.94				
Complications of cardiac/vascular device, implant, and	854	Injuries & Poisonings	0.110	0.020	1.58E-07	6.97E-06	-0.035	0.145	0.81	0.97	0.139	0.116	0.23	0.89				
Complications of transplants and reattached limbs	851	Injuries & Poisonings	-0.100	0.100	0.11	0.64												
Concussion	817	Injuries & Poisonings	0.000	0.070	0.66	0.97												
Contusion	916	Injuries & Poisonings	0.110	0.030	0.00	0.01												
Dislocation	830	Injuries & Poisonings	0.010	0.020	0.48	0.95	-0.229	0.220	0.30	0.92	-0.081	0.197	0.68	0.96				
Effects radiation NOS	990	Injuries & Poisonings	0.000	0.090	0.58	0.97												
Fracture of ankle and foot	801	Injuries & Poisonings	0.000	0.020	0.48	0.95					0.015	0.182	0.93	0.98				
Fracture of clavicle or scapula	803.3	Injuries & Poisonings	0.020	0.020	0.30	0.88												
Fracture of foot	801.1	Injuries & Poisonings	0.000	0.120	0.80	0.98												
Fracture of hand or wrist	804	Injuries & Poisonings	0.000	0.010	0.64	0.97	0.268	0.206	0.19	0.90	0.020	0.132	0.88	0.98				

Fracture of humerus	803.1	Injuries & Poisonings	0.000	0.020	0.81	0.98					-0.131	0.241	0.59	0.94				
Fracture of lower limb	800	Injuries & Poisonings	0.010	0.010	0.45	0.95	-0.204	0.218	0.35	0.92	-0.196	0.165	0.23	0.89				
Fracture of neck of femur	800.1	Injuries & Poisonings	0.000	0.010	0.62	0.97					0.010	0.202	0.96	0.98				
Fracture of patella	800.4	Injuries & Poisonings	0.000	0.030	0.70	0.97												
Fracture of pelvis	802	Injuries & Poisonings	0.000	0.030	0.97	1.00												
Fracture of radius and ulna	803.2	Injuries & Poisonings	0.000	0.010	0.48	0.95	0.436	0.175	0.01	0.34	-0.065	0.133	0.62	0.94				
Fracture of ribs	807	Injuries & Poisonings	0.000	0.030	0.90	1.00												
Fracture of tibia and fibula	800.3	Injuries & Poisonings	0.010	0.020	0.37	0.94	0.004	0.210	0.98	1.00	0.009	0.185	0.96	0.98				
Fracture of unspecified bones	809	Injuries & Poisonings	0.100	0.060	0.12	0.67												
Fracture of unspecified part of femur	800.2	Injuries & Poisonings	0.010	0.020	0.62	0.97												
Fracture of upper limb	803	Injuries & Poisonings	0.100	0.050	0.07	0.55												
Fracture of vertebral column without mention of spinal	805	Injuries & Poisonings	0.000	0.020	0.10	0.62					-0.081	0.200	0.69	0.96				
Hemorrhage or hematoma complicating a procedure	850	Injuries & Poisonings	0.030	0.010	0.13	0.67	0.093	0.131	0.48	0.92	-0.062	0.131	0.63	0.94				
Injuries to the nervous system	907	Injuries & Poisonings	0.000	0.030	0.93	1.00												
Internal derangement of knee	835	Injuries & Poisonings	0.000	0.000	0.73	0.97	0.079	0.060	0.19	0.90	-0.016	0.057	0.78	0.98	-0.038	0.164	0.82	0.99
Intracranial hemorrhage (injury)	818	Injuries & Poisonings	0.080	0.030	0.00	0.08												
Joint/ligament sprain	840.3	Injuries & Poisonings	0.020	0.080	0.81	0.98												
Mechanical complication of unspecified genitourinary	857	Injuries & Poisonings	0.030	0.020	0.23	0.79	-0.060	0.162	0.71	0.94	0.050	0.230	0.83	0.98				
Open wound of ear	870.2	Injuries & Poisonings	0.000	0.090	0.72	0.97												
Open wound of foot except toe(s) alone	871.3	Injuries & Poisonings	0.000	0.090	0.53	0.95												
Open wound of genital organs	870.8	Injuries & Poisonings	0.240	0.130	0.08	0.57												
Open wound of lip and mouth	870.5	Injuries & Poisonings	0.010	0.060	0.76	0.97												
Open wound of neck	870.6	Injuries & Poisonings	0.000	0.070	0.60	0.97												
Open wound of nose and sinus	870.4	Injuries & Poisonings	0.050	0.070	0.49	0.95												
Open wound of toe(s)	871.4	Injuries & Poisonings	0.340	0.100	0.00	0.04												
Open wound or laceration of eye or eyelid	870.1	Injuries & Poisonings	0.080	0.040	0.09	0.60												
Open wounds of extremities	871	Injuries & Poisonings	0.000	0.010	0.79	0.98	0.308	0.135	0.02	0.46	-0.142	0.132	0.28	0.93				
Open wounds of head; neck; and trunk	870	Injuries & Poisonings	-0.100	0.110	0.33	0.91												
Other open wound of head and face	870.3	Injuries & Poisonings	0.010	0.020	0.63	0.97	0.018	0.197	0.93	0.98	0.115	0.150	0.44	0.94				
Poisoning by agents primarily affecting the cardiovasc	972	Injuries & Poisonings	-0.100	0.100	0.22	0.79												
Poisoning by agents primarily affecting the gastrointes	973	Injuries & Poisonings	0.210	0.190	0.27	0.83												
Poisoning by analgesics, antipyretics, and antirheumat	965	Injuries & Poisonings	0.040	0.020	0.13	0.67					0.200	0.193	0.30	0.93				
Poisoning by anticonvulsants and anti-Parkinsonism d	966	Injuries & Poisonings	0.010	0.050	0.83	0.98												
Poisoning by drugs primarily affecting the autonomic r	971	Injuries & Poisonings	0.000	0.100	0.73	0.97												
Poisoning by hormones and synthetic substitutes	962	Injuries & Poisonings	0.000	0.100	0.87	0.99												
Poisoning by primarily systemic agents	963	Injuries & Poisonings	-0.100	0.180	0.37	0.94												
Poisoning by psychotropic agents	969	Injuries & Poisonings	0.000	0.020	0.97	1.00	-0.030	0.224	0.90	0.98								
Salicylates causing adverse effects in therapeutic use	965.3	Injuries & Poisonings	0.000	0.100	0.81	0.98												
Sepsis	994.2	Injuries & Poisonings	0.010	0.010	0.22	0.79	-0.071	0.119	0.55	0.92	-0.089	0.104	0.39	0.94				
Skull and face fracture and other intercranial injury	819	Injuries & Poisonings	0.000	0.020	0.39	0.94	-0.020	0.199	0.92	0.98	0.205	0.139	0.14	0.89				
Spinal cord injury without evidence of spinal bone inju	952	Injuries & Poisonings	0.000	0.110	0.58	0.97												
Sprains and strains	840	Injuries & Poisonings	0.000	0.050	0.93	1.00												
Toxic effect of (non-ethyl) alcohol and petroleum and (	981	Injuries & Poisonings	0.000	0.180	0.89	1.00												
Toxic effect of carbon monoxide	986	Injuries & Poisonings	0.180	0.120	0.16	0.72												
Toxic effect of noxious substances eaten as food	988	Injuries & Poisonings	0.180	0.180	0.33	0.91												
Toxic effect of other gases, fumes, or vapors	987	Injuries & Poisonings	0.000	0.100	0.92	1.00												
Toxic effect of other metals	985	Injuries & Poisonings	0.190	0.150	0.22	0.78												
Toxic effect of other substances, chiefly nonmedicinal	989	Injuries & Poisonings	0.150	0.140	0.28	0.86												
Toxic effect of venom	913	Injuries & Poisonings	0.110	0.080	0.18	0.75												
Traumatic amputation	872	Injuries & Poisonings	0.050	0.040	0.22	0.78												
Traumatic arthropathy	836	Injuries & Poisonings	0.020	0.040	0.57	0.97												
Adrenergics, inhalant medications		Medications	0.000	0.007	0.96	1.00	0.036	0.052	0.49	0.92	-0.076	0.046	0.10	0.86				
Agents acting on the renin-angiotensin system		Medications	0.043	0.005	1.01E-16	9.21E-15	0.048	0.034	0.16	0.86	0.037	0.030	0.21	0.89				
Anilides medications		Medications	-5.22E-05	0.005	0.99	1.00	-0.045	0.027	0.09	0.66	-0.007	0.028	0.79	0.98	0.044	0.062	0.47	0.90
Anti-inflammatory and antirheumatic products, non-steroids		Medications	-0.012	0.004	0.01	0.12	-0.042	0.031	0.17	0.86	0.030	0.034	0.38	0.94	-0.181	0.073	0.01	0.50
Antidepressant medications		Medications	0.011	0.007	0.10	0.63	-0.019	0.062	0.76	0.97	-0.092	0.055	0.09	0.84				
Antiglaucoma preparations and miotics		Medications	0.007	0.016	0.67	0.97												
Antihistamines for systemic use		Medications	-0.005	0.010	0.59	0.97	0.088	0.064	0.17	0.86	-0.013	0.059	0.83	0.98				
Antihypertensive medications		Medications	0.005	0.015	0.75	0.97												
Antimigraine preparations		Medications	-0.027	0.016	0.09	0.61												
Antithrombotic agents		Medications	0.068	0.005	2.85E-42	5.94E-40	-0.022	0.036	0.53	0.92	0.007	0.030	0.81	0.98				
Beta blocking agents		Medications	0.066	0.007	1.31E-22	1.74E-20	-0.003	0.051	0.96	0.99	0.000	0.043	1.00	1.00				
Calcium channel blockers		Medications	0.026	0.007	2.13E-04	0.01	0.041	0.032	0.20	0.90	-0.007	0.038	0.86	0.98				
Diuretic medications		Medications	0.011	0.007	0.11	0.65	0.007	0.036	0.84	0.98	0.033	0.046	0.48	0.94				
Drugs affecting bone structure and mineralization		Medications	0.001	0.014	0.93	1.00												
Drugs for peptic ulcer and gastro-esophageal reflux disease		Medications	0.007	0.005	0.23	0.79	0.062	0.041	0.13	0.79	0.040	0.035	0.26	0.92				
Drugs used in diabetes		Medications	0.037	0.010	0.00	0.00	0.003	0.042	0.94	0.99	0.014	0.033	0.67	0.96				
Glucocorticoid medications		Medications	0.003	0.009	0.75	0.97	-0.159	0.070	0.02	0.47	-0.121	0.059	0.04	0.61				
HMG CoA reductase inhibitors		Medications	0.096	0.005	2.20E-87	6.42E-85	0.117	0.035	0.00	0.08	0.065	0.028	0.02	0.40	0.053	0.079	0.50	0.90
Immunosuppressant medications		Medications	-0.013	0.019	0.48	0.95												
Opiod medications		Medications	0.016	0.008	0.05	0.44	-0.064	0.052	0.21	0.91	-0.057	0.057	0.32	0.93				
Salicylic acid and derivatives		Medications	0.065	0.005	2.43E-37	4.44E-35	-0.011	0.036	0.76	0.97	-0.006	0.031	0.85	0.98				
Thyroid preparations		Medications	0.002	0.008	0.76	0.97					0.122	0.044	0.01	0.22				
Vasodilators used in cardiac diseases		Medications	0.130	0.015	9.72E-19	1.09E-16												
Acute reaction to stress	300.8	Mental And Behavioral Dis	0.000	0.120	0.54	0.96												
Addicted to Medication		Mental And Behavioral Dis	0.020	0.034	0.55	0.96												
Addiction behavior		Mental And Behavioral Dis	0.025	0.026	0.34	0.91												
Adjustment reaction	304	Mental And Behavioral Dis	0.010	0.060	0.82	0.98												
Agorophobia, social phobia, and panic disorder	300.12	Mental And Behavioral Dis	0.080	0.070	0.28	0.86												
Alcohol addiction		Mental And Behavioral Dis	0.014	0.020	0.49	0.95												
Alcohol-related disorders	317	Mental And Behavioral Dis	0.030	0.030	0.34	0.92					-0.123	0.232	0.60	0.94				
Alcoholic liver damage	317.11	Mental And Behavioral Dis	0.150	0.040	0.00	0.01												
Alcoholism	317.1	Mental And Behavioral Dis	0.060	0.030	0.09	0.59												
Alteration of consciousness	291.8	Mental And Behavioral Dis	0.000	0.060	0.67	0.97												
Altered mental status	292.4	Mental And Behavioral Dis	0.000	0.030	0.40	0.94	0.025	0.209	0.90	0.98	0.281	0.192	0.14	0.89				
Alzheimer's disease	290.11	Mental And Behavioral Dis	-0.100	0.060	0.08	0.57												
Antisocial/borderline personality disorder	301.2	Mental And Behavioral Dis	0.070	0.100	0.46	0.95												
Anxiety		Mental And Behavioral Dis	-0.017	0.020	0.41	0.95												
Anxiety Criteria 1		Mental And Behavioral Dis	0.004	0.015	0.78	0.98												
Anxiety Criteria 2		Mental And Behavioral Dis	-0.003	0.007	0.64	0.97					0.043	0.062	0.49	0.94				
Anxiety Criteria 3		Mental And Behavioral Dis	-0.009	0.007	0.17	0.74	0.065	0.069	0.35	0.92	0.088	0.063	0.16	0.89				
Anxiety Criteria 4		Mental And Behavioral Dis	-0.007	0.006	0.22	0.78	0.059	0.067	0.38	0.92	0.060	0.059	0.31	0.93				
Anxiety Criteria 5		Mental And Behavioral Dis	-0.007	0.007	0.35	0.92					0.024	0.070	0.73	0.98				
Anxiety Criteria 6		Mental And Behavioral Dis	-0.009	0.007	0.17	0.74					0.130	0.063	0.04	0.60				
Anxiety disorder	300.1	Mental And Behavioral Dis	0.000	0.040	0.90	1.00												
Anxiety disorders	300	Mental And Behavioral Dis	0.030	0.080	0.68	0.97												
Aphasia/speech disturbance	292.1	Mental And Behavioral Dis	0.050	0.040	0.18	0.75												
Bipolar	296.1	Mental And Behavioral Dis	0.000	0.040	0.41	0.95	0.138	0.175	0.43	0.92								
Bipolar Affective Disorder Criteria 1		Mental And Behavioral Dis	-0.004	0.006	0.48	0.95	-0.066	0.062	0.29	0.92	0.021	0.054	0.70	0.96				
Bipolar Affective Disorder Criteria 3		Mental And Behavioral Dis	0.012	0.018	0.50	0.95												
Bipolar Affective Disorder Manifestation Criteria Score		Mental And Behavioral Dis	0.000	0.001	0.57	0.97	-0.002	0.005	0.64	0.93	0.003	0.005	0.54	0.94	-0.004	0.010	0.65	0.96
Current vs. ever smoking		Mental And Behavioral Dis	-0.010	0.006	0.11	0.65	-0.074	0.045	0.10	0.66	-0.134	0.052	0.01	0.33				
Current vs. never smoking		Mental And Behavioral Dis	-0.003	0.006	0.58	0.97	-0.053	0.038	0.16	0.86	-0.148	0.042	0.00	0.04				
Current vs. non-current alcohol drinking		Mental And Behavioral Dis	-0.013	0.009	0.18	0.75	-0.025	0.052	0.63	0.93	-0.017	0.052	0.74	0.98				
Delirium dementia and amnestic and other cognitive d	290	Mental And Behavioral Dis	-0.100	0.070	0.15	0.70												
Delirium due to conditions classified elsewhere	290.2	Mental And Behavioral Dis	0.070	0.040	0.07	0.55												
Dementias	290.1	Mental And Behavioral Dis	0.040	0.070	0.48	0.95												
Depression		Mental And Behavioral Dis	0.001	0.007	0.83	0.98					0.022	0.070	0.76	0.98				
Depression Criteria 1		Mental And Behavioral Dis	-0.002	0.005	0.69	0.97	-0.004	0.046	0.93	0.98	-0.026	0.044	0.55	0.94	0.043	0.074	0.56	0.92
Depression Criteria 2		Mental And Behavioral Dis	0.007	0.006	0.25	0.81	0.047	0.069	0.49	0.92	0.033	0.065	0.61	0.94				
Depression Criteria 3		Mental And Behavioral Dis	-0.002	0.005	0.76	0.97	-0.054	0.057	0.35	0.92	-0.001	0.053	0.98	1.00				
Depression score		Mental And Behavioral Dis	0.009	0.009	0.33	0.91	-0.051	0.089	0.57	0.92	0.111	0.085	0.19	0.89	0.105	0.145	0.47	0.90
Develomental delays and disorders	315	Mental And Behavioral Dis	0.000	0.190	0.98	1.00												
Dissociative disorder	303.1	Mental And Behavioral Dis	0.000	0.180	0.96	1.00												
Drug Addiction		Mental And Behavioral Dis	-0.075	0.049	0.13	0.67												
Dysthymic disorder	300.4	Mental And Behavioral Dis	0.260	0.180	0.16	0.72												
Ever Physicall Dependent on Alcohol		Mental And Behavioral Dis	-0.001	0.047	0.99	1.00												
Ever vs. never drinking alcohol		Mental And Behavioral Dis	-0.028	0.010	0.00	0.08	-0.020	0.032	0.53	0.92	-0.062	0.025	0.01	0.33	0.011	0.055	0.85	0.99
Ever vs. never smoking		Mental And Behavioral Dis	0.003	0.004	0.48	0.95	-0.003	0.027	0.92	0.98	-0.063	0.029	0.03	0.56	0.044	0.053	0.41	0.88
Generalized anxiety disorder	300.11	Mental And Behavioral Dis	0.000	0.120	0.67	0.97												
Hallucinations	292.6	Mental And Behavioral Dis	-0.100	0.170	0.49	0.95												
Major depressive disorder	296.22	Mental And Behavioral Dis	0.020	0.050	0.68	0.97												
Mean packs of cigarettes smoked/day		Mental And Behavioral Dis	0.002	0.006	0.77	0.97	-0.014	0.041	0.74	0.97	0.043	0.048	0.38	0.94				

Memory loss	292.3	Mental And Behavioral Dis	0.000	0.060	0.31	0.88												
Mild cognitive impairment	292.2	Mental And Behavioral Dis	0.070	0.130	0.56	0.97												
Mood disorders	296	Mental And Behavioral Dis	0.160	0.160	0.31	0.88												
Neurological disorders	292	Mental And Behavioral Dis	0.090	0.050	0.10	0.62												
Obsessive-compulsive disorders	300.3	Mental And Behavioral Dis	0.000	0.180	0.68	0.97												
Ongoing Addiction Behavior		Mental And Behavioral Dis	-0.079	0.053	0.14	0.68												
Ongoing Alcohol Addiction		Mental And Behavioral Dis	0.002	0.042	0.97	1.00												
Ongoing Behavioral or Misc Addiction		Mental And Behavioral Dis	0.008	0.013	0.51	0.95												
Ongoing Medication Addiction		Mental And Behavioral Dis	-0.048	0.070	0.49	0.95												
Other persistent mental disorders due to conditions cla	290.3	Mental And Behavioral Dis	0.340	0.160	0.04	0.39												
Pack-years of smoking		Mental And Behavioral Dis	0.003	0.003	0.26	0.82	-0.032	0.027	0.24	0.91	0.017	0.029	0.56	0.94	0.007	0.056	0.90	0.99
Paranoid disorders	295.2	Mental And Behavioral Dis	0.000	0.090	0.91	1.00												
Personality disorders	301	Mental And Behavioral Dis	0.140	0.100	0.14	0.68												
Psychogenic disorder	303.3	Mental And Behavioral Dis	0.100	0.050	0.06	0.52												
Psychosis	295.3	Mental And Behavioral Dis	0.000	0.100	0.46	0.95												
Schizophrenia	295.1	Mental And Behavioral Dis	0.030	0.050	0.55	0.96	-0.447	0.167	0.01	0.30	0.114	0.221	0.61	0.94				
Sexual and gender identity disorders	302	Mental And Behavioral Dis	0.050	0.080	0.48	0.95												
Somatoform disorder	303.4	Mental And Behavioral Dis	0.050	0.100	0.63	0.97												
Specific nonpsychotic mental disorders due to brain da	291.4	Mental And Behavioral Dis	0.000	0.130	0.95	1.00												
Speech and language disorder	315.2	Mental And Behavioral Dis	0.200	0.180	0.27	0.84												
Substance addiction and disorders	316	Mental And Behavioral Dis	-0.200	0.140	0.07	0.56												
Swelling, mass, or lump in head and neck [Space-occu	293.1	Mental And Behavioral Dis	0.000	0.030	0.25	0.81	0.320	0.208	0.13	0.77								
Symptoms involving head and neck	293	Mental And Behavioral Dis	0.000	0.020	0.84	0.98	-0.080	0.158	0.61	0.92	-0.076	0.156	0.62	0.94				
Tension headache	306.9	Mental And Behavioral Dis	0.070	0.050	0.15	0.69												
Transient mental disorders due to conditions classified	291.1	Mental And Behavioral Dis	0.030	0.090	0.68	0.97												
Vascular dementia	290.16	Mental And Behavioral Dis	-0.300	0.120	0.00	0.07												
Years smoked cigarettes		Mental And Behavioral Dis	-0.014	0.039	0.73	0.97	-0.554	0.289	0.05	0.62	0.021	0.341	0.95	0.98	0.043	0.619	0.94	0.99
Acquired deformities of finger	736.2	Musculoskeletal	0.000	0.070	0.50	0.95												
Acquired foot deformities	735	Musculoskeletal	0.000	0.080	0.81	0.98												
Acquired spondylolisthesis	738.4	Musculoskeletal	0.000	0.030	0.95	1.00	0.006	0.180	0.97	1.00								
Acquired toe deformities	735.2	Musculoskeletal	0.000	0.020	0.16	0.72					-0.019	0.191	0.92	0.98				
Acute osteomyelitis	710.11	Musculoskeletal	0.000	0.190	0.98	1.00												
Ankylosing spondylitis	715.2	Musculoskeletal	-0.200	0.090	0.01	0.13												
Ankylosis of joint	741.1	Musculoskeletal	0.060	0.120	0.60	0.97												
Arthropathy NOS	716.9	Musculoskeletal	0.000	0.000	0.65	0.97	0.005	0.055	0.93	0.98	0.103	0.049	0.04	0.56	-0.121	0.162	0.45	0.90
Arthropathy associated with infections	711	Musculoskeletal	0.000	0.170	0.94	1.00												
Arthropathy associated with other disorders classified	713	Musculoskeletal	0.000	0.140	0.63	0.97												
Articular cartilage disorder	742.8	Musculoskeletal	0.000	0.040	0.18	0.75												
Aseptic necrosis of bone	733.4	Musculoskeletal	0.060	0.050	0.30	0.87												
Body fat %		Musculoskeletal	-0.022	0.010	0.03	0.38	0.124	0.070	0.07	0.62	0.055	0.059	0.35	0.94	-0.109	0.123	0.37	0.88
Body mass index		Musculoskeletal	0.004	0.002	0.04	0.43	0.012	0.012	0.29	0.92	0.014	0.011	0.23	0.89	-0.028	0.020	0.17	0.73
Body surface area		Musculoskeletal	0.000	0.001	0.89	1.00	0.008	0.015	0.58	0.92	-0.011	0.009	0.21	0.89				
Bursitis	726.3	Musculoskeletal	0.000	0.030	0.93	1.00												
Bursitis disorders	727.2	Musculoskeletal	0.000	0.100	0.91	1.00												
Calcaneal spur; Exostosis NOS	726.4	Musculoskeletal	-0.200	0.160	0.17	0.72												
Chronic osteomyelitis	710.12	Musculoskeletal	0.000	0.090	0.65	0.97												
Contracture of joint	739	Musculoskeletal	-0.100	0.060	0.04	0.40												
Contracture of palmar fascia [Dupuytren's disease]	728.71	Musculoskeletal	0.010	0.010	0.44	0.95												
Contracture of tendon (sheath)	727.7	Musculoskeletal	0.040	0.090	0.67	0.97												
Cyst of bone	733.2	Musculoskeletal	0.000	0.110	0.75	0.97												
Degeneration of intervertebral disc	722.6	Musculoskeletal	0.010	0.020	0.62	0.97	-0.031	0.153	0.84	0.98	0.128	0.135	0.34	0.94				
Difficulty in walking	741.3	Musculoskeletal	0.050	0.150	0.71	0.97												
Disorders of muscle, ligament, and fascia	728	Musculoskeletal	0.080	0.090	0.38	0.94												
Displacement of intervertebral disc	722.1	Musculoskeletal	0.020	0.060	0.68	0.97												
Enthesopathy	726.1	Musculoskeletal	0.000	0.010	0.60	0.97	-0.055	0.086	0.52	0.92	-0.014	0.074	0.85	0.98	0.316	0.200	0.11	0.71
Fasciitis	728.7	Musculoskeletal	0.000	0.050	0.40	0.94												
Flat foot	735.1	Musculoskeletal	0.000	0.090	0.77	0.97												
Ganglion and cyst of synovium, tendon, and bursa	727.4	Musculoskeletal	0.020	0.010	0.15	0.69	-0.089	0.114	0.43	0.92	0.478	0.122	8.65E-05	0.03	0.078	0.236	0.74	0.99
Hallux rigidus	735.23	Musculoskeletal	0.000	0.020	0.70	0.97												
Hallux valgus (Bunion)	735.3	Musculoskeletal	0.000	0.010	0.71	0.97	-0.193	0.087	0.03	0.49	0.162	0.094	0.09	0.82	-0.146	0.207	0.48	0.90
Hammer toe (acquired)	735.21	Musculoskeletal	0.070	0.020	0.01	0.10												
Hand grip strength		Musculoskeletal	-0.003	0.013	0.81	0.98	0.191	0.101	0.06	0.62	0.048	0.086	0.57	0.94	-0.112	0.149	0.45	0.90
Hand preference		Musculoskeletal	-0.002	0.006	0.66	0.97	-0.012	0.043	0.77	0.97	-0.027	0.040	0.50	0.94				
Heel bone mineral density T-score		Musculoskeletal	0.000	0.002	0.97	1.00	0.023	0.016	0.14	0.79	-0.004	0.012	0.74	0.98	-0.006	0.025	0.82	0.99
Height		Musculoskeletal	0.002	0.011	0.82	0.98	-0.008	0.073	0.91	0.98	0.034	0.067	0.62	0.94	0.066	0.120	0.59	0.92
Hemarthrosis	741.5	Musculoskeletal	0.120	0.080	0.14	0.68												
Hip circumference		Musculoskeletal	0.001	0.002	0.50	0.95	0.008	0.011	0.48	0.92	0.005	0.011	0.66	0.96	-0.015	0.021	0.47	0.90
Impedance of whole body		Musculoskeletal	-0.461	0.113	4.22E-05	0.00	-0.372	0.821	0.65	0.93	0.473	0.810	0.56	0.94	1.432	1.394	0.30	0.84
Infective connective tissue disorders	712	Musculoskeletal	-0.200	0.150	0.17	0.72												
Intervertebral disc disorder with myelopathy	722.7	Musculoskeletal	0.000	0.050	0.61	0.97	-0.280	0.241	0.25	0.91								
Joint effusions	741.4	Musculoskeletal	0.010	0.030	0.71	0.97	-0.004	0.202	0.98	1.00	-0.025	0.198	0.90	0.98				
Juvenile osteochondrosis	732.1	Musculoskeletal	0.130	0.100	0.19	0.75												
Kyphoscoliosis and scoliosis	737.3	Musculoskeletal	0.000	0.050	0.39	0.94												
Kyphosis (acquired)	737.1	Musculoskeletal	0.080	0.130	0.55	0.96												
Laxity of ligament or hypermobility syndrome	728.2	Musculoskeletal	0.000	0.060	0.64	0.97												
Loose body in joint	742.1	Musculoskeletal	-0.100	0.080	0.11	0.65												
Malunion and nonunion of fracture	733.8	Musculoskeletal	0.000	0.020	0.77	0.97					0.209	0.215	0.33	0.94				
Osteitis deformans [Paget's disease of bone]	731.1	Musculoskeletal	-0.200	0.170	0.22	0.79												
Osteoarthritis; localized	740.1	Musculoskeletal	0.000	0.000	0.14	0.68	-0.064	0.116	0.58	0.92	0.251	0.125	0.04	0.62				
Osteoarthrosis NOS	740.9	Musculoskeletal	0.000	0.020	0.99	1.00	-0.111	0.161	0.49	0.92	0.314	0.158	0.05	0.62				
Osteoarthrosis, generalized	740.2	Musculoskeletal	0.020	0.050	0.64	0.97												
Osteoarthrosis, localized, primary	740.11	Musculoskeletal	0.000	0.010	0.79	0.98	0.095	0.096	0.32	0.92	0.073	0.090	0.42	0.94				
Osteoarthrosis, localized, secondary	740.12	Musculoskeletal	0.040	0.050	0.41	0.95												
Osteochondritis dissecans	732.7	Musculoskeletal	-0.400	0.260	0.07	0.57												
Osteochondropathies	732	Musculoskeletal	0.050	0.100	0.62	0.97												
Osteomyelitis, periostitis, and other infections involvin	710	Musculoskeletal	0.000	0.180	0.60	0.97												
Osteopenia or other disorder of bone and cartilage	743.9	Musculoskeletal	0.040	0.040	0.28	0.86												
Osteoporosis		Musculoskeletal	0.003	0.017	0.87	0.99												
Osteoporosis NOS	743.11	Musculoskeletal	0.000	0.020	0.14	0.68					-0.062	0.176	0.72	0.97				
Other acquired deformities of limbs	736	Musculoskeletal	0.000	0.040	0.53	0.95												
Other acquired musculoskeletal deformity	738	Musculoskeletal	0.000	0.060	0.45	0.95												
Other allied disorders of spine	721.8	Musculoskeletal	0.000	0.090	0.63	0.97												
Other and unspecified disc disorder	722.9	Musculoskeletal	0.000	0.020	0.10	0.62	-0.136	0.164	0.41	0.92	0.113	0.175	0.52	0.94				
Other arthropathies	716	Musculoskeletal	0.050	0.090	0.57	0.97												
Other derangement of joint	742.9	Musculoskeletal	0.000	0.020	0.45	0.95	-0.160	0.156	0.30	0.92	-0.228	0.183	0.21	0.89				
Other disorders of bone and cartilage	733	Musculoskeletal	0.000	0.030	0.83	0.98					-0.184	0.224	0.41	0.94				
Other disorders of cervical region	723	Musculoskeletal	0.000	0.050	0.59	0.97	-0.280	0.241	0.25	0.91								
Other disorders of soft tissues	729	Musculoskeletal	0.000	0.010	0.85	0.99	0.035	0.087	0.68	0.93	-0.133	0.087	0.13	0.89				
Other disorders of synovium, tendon, and bursa	727	Musculoskeletal	0.000	0.040	0.73	0.97	-0.291	0.242	0.23	0.91								
Other inflammatory spondylopathies	715	Musculoskeletal	0.000	0.060	0.92	1.00												
Other symptoms referable to back	724.8	Musculoskeletal	0.150	0.150	0.32	0.89												
Other unspecified back disorders	724.9	Musculoskeletal	0.000	0.040	0.51	0.95	-0.193	0.221	0.38	0.92								
Pain in joint	745	Musculoskeletal	0.010	0.010	0.39	0.94	0.066	0.077	0.39	0.92	0.074	0.071	0.29	0.93	-0.001	0.228	1.00	1.00
Panniculitis	729.3	Musculoskeletal	-0.100	0.160	0.52	0.95												
Pathologic fracture	743.2	Musculoskeletal	0.030	0.080	0.63	0.97												
Pathologic fracture of vertebrae	743.21	Musculoskeletal	0.140	0.070	0.04	0.38												
Pathological, developmental or recurrent dislocation	742.2	Musculoskeletal	0.090	0.140	0.52	0.95												
Peripheral enthesopathies and allied syndromes	726	Musculoskeletal	0.010	0.010	0.16	0.72	-0.034	0.070	0.62	0.92	0.059	0.061	0.33	0.94	-0.013	0.182	0.94	0.99
Polymyalgia Rheumatica	717	Musculoskeletal	-0.100	0.080	0.22	0.79												
Postlaminectomy syndrome	722.8	Musculoskeletal	0.000	0.120	0.85	0.99												
Pyogenic arthritis	711.1	Musculoskeletal	-0.100	0.050	0.01	0.13												
Reiter's disease	711.2	Musculoskeletal	-0.100	0.240	0.62	0.97												
Rheumatism, unspecified and fibrositis	729.1	Musculoskeletal	0.090	0.070	0.25	0.81												
Rheumatoid arthritis	714.1	Musculoskeletal	0.000	0.020	0.55	0.96	-0.356	0.213	0.09	0.66	0.143	0.143	0.32	0.93				
Rheumatoid arthritis and other inflammatory polyarthi	714	Musculoskeletal	-0.100	0.070	0.10	0.62												
Rupture of synovium	727.5	Musculoskeletal	0.010	0.040	0.77	0.98												
Rupture of tendon, nontraumatic	727.6	Musculoskeletal	0.040	0.050	0.45	0.95												
Sacroiliitis NEC	715.1	Musculoskeletal	-0.200	0.110	0.03	0.34												
Spinal stenosis	720	Musculoskeletal	0.010	0.010	0.58	0.97	-0.029	0.111	0.80	0.97	0.031	0.111	0.78	0.98				
Spondylosis and allied disorders	721	Musculoskeletal	0.000	0.030	0.87	0.99					-0.124	0.194	0.52	0.94				
Spondylosis with myelopathy	721.2	Musculoskeletal	0.000	0.080	0.35	0.92												
Spondylosis without myelopathy	721.1	Musculoskeletal	0.000	0.020	0.87	0.99	-0.056	0.130	0.67	0.93	0.132	0.111	0.23	0.89				
Stiffness of joint	741.2	Musculoskeletal	0.010	0.040	0.82	0.98												

Stress fracture	743.4	Musculoskeletal	0.000	0.220	0.75	0.97												
Symptoms and disorders of the joints	741	Musculoskeletal	0.030	0.030	0.38	0.94												
Synoviopathy	726.2	Musculoskeletal	0.080	0.060	0.20	0.76												
Synovitis and tenosynovitis	727.1	Musculoskeletal	0.000	0.010	0.79	0.98	-0.140	0.108	0.19	0.90	0.097	0.104	0.35	0.94	-0.251	0.244	0.30	0.84
Torticollis	723.1	Musculoskeletal	0.000	0.200	0.84	0.98												
Unspecified monoarthritis	716.2	Musculoskeletal	0.000	0.000	0.62	0.97	-0.009	0.065	0.90	0.98	0.090	0.056	0.11	0.88	-0.065	0.193	0.74	0.99
Unspecified osteomyelitis	710.19	Musculoskeletal	0.000	0.050	0.72	0.97												
Villonodular synovitis	741.6	Musculoskeletal	0.000	0.140	0.85	0.99												
Waist circumference		Musculoskeletal	0.074	0.021	0.00	0.01	0.029	0.143	0.84	0.98	0.070	0.121	0.56	0.94	-0.388	0.195	0.05	0.70
Waist/hip ratio		Musculoskeletal	0.000	0.000	0.00	0.00	0.000	0.001	0.60	0.92	0.000	0.001	0.78	0.98	-0.003	0.001	0.05	0.70
Weight		Musculoskeletal	0.003	0.002	0.03	0.32	0.010	0.011	0.40	0.92	0.014	0.010	0.16	0.89	-0.016	0.018	0.36	0.88
Whole body fat mass		Musculoskeletal	-0.003	0.013	0.81	0.98	0.145	0.093	0.12	0.74	0.064	0.075	0.39	0.94	-0.141	0.123	0.25	0.84
Whole body fat-free mass		Musculoskeletal	0.029	0.010	0.00	0.07	0.006	0.008	0.46	0.92	0.005	0.007	0.52	0.94	-0.011	0.014	0.43	0.90
Whole body water mass		Musculoskeletal	0.023	0.008	0.00	0.05	0.006	0.008	0.46	0.92	0.004	0.007	0.54	0.94	-0.010	0.014	0.47	0.90
Benign neoplasm of adrenal gland	227.1	Neoplasms	0.000	0.080	0.75	0.97												
Benign neoplasm of bone and articular cartilage	213	Neoplasms	0.000	0.060	0.24	0.80												
Benign neoplasm of brain, cranial nerves, meninges	225.1	Neoplasms	0.010	0.030	0.64	0.97					-0.116	0.238	0.63	0.94				
Benign neoplasm of colon	208	Neoplasms	0.000	0.000	0.26	0.82	-0.101	0.051	0.05	0.62	-0.010	0.047	0.84	0.98	0.215	0.090	0.02	0.53
Benign neoplasm of eye	224	Neoplasms	0.000	0.130	0.80	0.98												
Benign neoplasm of kidney and other urinary organs	223	Neoplasms	0.000	0.080	0.27	0.84												
Benign neoplasm of lip, oral cavity, and pharynx	210	Neoplasms	0.000	0.030	0.36	0.93					0.207	0.216	0.34	0.94				
Benign neoplasm of lymph nodes	229.1	Neoplasms	0.080	0.190	0.64	0.97												
Benign neoplasm of male genital organs	222	Neoplasms	0.000	0.090	0.85	0.99												
Benign neoplasm of other female genital organs	221	Neoplasms	0.020	0.080	0.76	0.97												
Benign neoplasm of other parts of digestive system	211	Neoplasms	0.000	0.010	0.39	0.94	-0.139	0.135	0.30	0.92	-0.064	0.103	0.53	0.94				
Benign neoplasm of ovary	220	Neoplasms	0.020	0.020	0.38	0.94	0.518	0.199	0.01	0.33								
Benign neoplasm of parathyroid gland	227.2	Neoplasms	0.000	0.050	0.11	0.65												
Benign neoplasm of pituitary gland and craniopharyng	227.3	Neoplasms	-0.100	0.060	0.05	0.49	-0.262	0.236	0.27	0.92								
Benign neoplasm of respiratory and intrathoracic orga	212	Neoplasms	0.040	0.050	0.38	0.94												
Benign neoplasm of skin	216	Neoplasms	0.000	0.010	0.85	0.99	0.044	0.126	0.73	0.96	-0.070	0.115	0.54	0.94	0.491	0.162	0.00	0.15
Benign neoplasm of spinal cord, meninges	225.2	Neoplasms	0.080	0.120	0.47	0.95												
Benign neoplasm of thyroid glands	226	Neoplasms	0.000	0.060	0.87	0.99												
Benign neoplasm of unspecified sites	229	Neoplasms	0.000	0.020	0.65	0.97	-0.111	0.202	0.58	0.92	0.100	0.175	0.57	0.94				
Biliary cancer		Neoplasms	-0.006	0.053	0.91	1.00												
Bladder cancer		Neoplasms	-0.010	0.028	0.73	0.97												
Bone cancer	170.1	Neoplasms	0.000	0.080	0.65	0.97												
Bone marrow or stem cell transplant	860	Neoplasms	0.210	0.130	0.12	0.66												
Brain cancer		Neoplasms	0.019	0.039	0.61	0.97												
Breast cancer		Neoplasms	-0.001	0.010	0.92	1.00												
Breast cancer [female]	174.1	Neoplasms	0.000	0.020	0.89	1.00	0.095	0.156	0.54	0.92	0.046	0.169	0.79	0.98				
Cancer of bladder	189.2	Neoplasms	0.000	0.030	0.80	0.98												
Cancer of brain	191.11	Neoplasms	0.050	0.030	0.16	0.72												
Cancer of brain and nervous system	191.1	Neoplasms	0.050	0.130	0.71	0.97												
Cancer of bronchus; lung	165.1	Neoplasms	0.000	0.010	0.95	1.00	0.301	0.159	0.06	0.62	-0.055	0.201	0.78	0.98				
Cancer of connective tissue	170.2	Neoplasms	0.000	0.050	0.47	0.95												
Cancer of esophagus	150	Neoplasms	0.020	0.030	0.44	0.95												
Cancer of eye	190	Neoplasms	0.010	0.070	0.89	1.00												
Cancer of hypopharynx	149.3	Neoplasms	0.170	0.110	0.12	0.66												
Cancer of intrathoracic organs	164	Neoplasms	-0.200	0.160	0.12	0.67												
Cancer of larynx	149.4	Neoplasms	0.000	0.050	0.98	1.00												
Cancer of larynx, pharynx, nasal cavities	149	Neoplasms	-0.100	0.150	0.32	0.90												
Cancer of lip	145.1	Neoplasms	0.040	0.120	0.75	0.97												
Cancer of liver and intrahepatic bile duct	155	Neoplasms	0.000	0.050	0.25	0.82												
Cancer of major salivary glands	145.3	Neoplasms	0.010	0.090	0.90	1.00												
Cancer of mouth	145	Neoplasms	0.000	0.070	0.96	1.00												
Cancer of nasopharynx	149.2	Neoplasms	0.060	0.150	0.68	0.97												
Cancer of of nasal cavities	149.9	Neoplasms	0.030	0.120	0.74	0.97												
Cancer of oropharynx	149.1	Neoplasms	0.030	0.050	0.51	0.95												
Cancer of other endocrine glands	194	Neoplasms	0.180	0.100	0.07	0.55												
Cancer of other female genital organs	184	Neoplasms	-0.100	0.090	0.28	0.86												
Cancer of other female genital organs (excluding uteru	184.2	Neoplasms	0.000	0.050	0.70	0.97												
Cancer of other lymphoid, histiocytic tissue	202	Neoplasms	0.000	0.090	0.64	0.97												
Cancer of other male genital organs	187	Neoplasms	0.000	0.150	0.72	0.97												
Cancer of prostate	185	Neoplasms	0.000	0.010	0.51	0.95	-0.040	0.070	0.57	0.92	-0.013	0.118	0.91	0.98				
Cancer of stomach	151	Neoplasms	0.000	0.030	0.86	0.99												
Cancer of the gums	145.4	Neoplasms	-0.200	0.180	0.21	0.77												
Cancer of the mouth floor	145.5	Neoplasms	0.020	0.140	0.86	0.99												
Cancer of tongue	145.2	Neoplasms	0.000	0.050	0.48	0.95												
Cancer of urinary organs (incl. kidney and bladder)	189	Neoplasms	-0.100	0.170	0.35	0.92												
Cancer within the respiratory system	165	Neoplasms	0.000	0.060	0.29	0.86												
Cancer, suspected or other	195	Neoplasms	0.010	0.030	0.61	0.97												
Carcinoma in situ of skin	172.3	Neoplasms	0.000	0.030	0.90	1.00												
Cervical cancer	180.1	Neoplasms	0.000	0.060	0.85	0.99												
Cervical cancer		Neoplasms	-0.026	0.039	0.51	0.95												
Cervical intraepithelial neoplasia [CIN] [Cervical dysp	180.3	Neoplasms	0.050	0.020	0.02	0.25	-0.147	0.120	0.22	0.91	-0.063	0.160	0.70	0.96				
Chemotherapy	197	Neoplasms	0.000	0.110	0.94	1.00												
Colon cancer	153.2	Neoplasms	0.000	0.010	0.18	0.74	-0.063	0.128	0.62	0.92	0.106	0.156	0.50	0.94				
Colorectal cancer	153	Neoplasms	0.000	0.160	0.97	1.00												
Colorectal cancer		Neoplasms	-0.003	0.014	0.81	0.98												
Esophageal cancer		Neoplasms	0.031	0.035	0.38	0.94												
Head cancer		Neoplasms	0.017	0.027	0.52	0.95												
Hemangioma and lymphangioma, any site	228	Neoplasms	0.000	0.020	0.25	0.82												
Hodgkin's disease	201	Neoplasms	0.000	0.070	0.97	1.00												
Kidney cancer		Neoplasms	0.034	0.027	0.20	0.77												
Large cell lymphoma	202.24	Neoplasms	0.050	0.030	0.13	0.67												
Leukemia	204	Neoplasms	-0.100	0.080	0.17	0.72												
Leukemia cancer		Neoplasms	0.015	0.029	0.59	0.97												
Lipoma	214	Neoplasms	0.000	0.040	0.20	0.76	0.021	0.204	0.92	0.98	0.020	0.204	0.92	0.98				
Lipoma of skin and subcutaneous tissue	214.1	Neoplasms	0.000	0.010	0.40	0.94	-0.020	0.071	0.78	0.97	0.028	0.083	0.74	0.98	-0.197	0.205	0.34	0.88
Lung cancer		Neoplasms	0.027	0.019	0.15	0.69												
Lymphoid leukemia	204.1	Neoplasms	-0.100	0.180	0.36	0.93												
Lymphoid leukemia, acute	204.11	Neoplasms	-0.100	0.130	0.34	0.92												
Lymphoid leukemia, chronic	204.12	Neoplasms	0.010	0.040	0.80	0.98												
Malignant neoplasm of bladder	189.21	Neoplasms	0.000	0.020	0.49	0.95					0.127	0.169	0.45	0.94				
Malignant neoplasm of female breast	174.11	Neoplasms	0.000	0.000	0.76	0.97	-0.048	0.082	0.55	0.92	-0.085	0.082	0.30	0.93	0.173	0.132	0.19	0.73
Malignant neoplasm of gallbladder and extrahepatic bi	159.3	Neoplasms	0.030	0.050	0.52	0.95												
Malignant neoplasm of head, face, and neck	195.3	Neoplasms	0.060	0.140	0.65	0.97												
Malignant neoplasm of kidney, except pelvis	189.11	Neoplasms	0.060	0.020	0.02	0.23					-0.110	0.231	0.63	0.94				
Malignant neoplasm of liver, primary	155.1	Neoplasms	0.200	0.060	1.61E-03	0.03												
Malignant neoplasm of other and ill-defined sites withi	159	Neoplasms	-0.100	0.110	0.09	0.61												
Malignant neoplasm of other urinary organs	189.4	Neoplasms	0.000	0.080	0.64	0.97												
Malignant neoplasm of ovary	184.11	Neoplasms	0.000	0.030	0.84	0.98					0.021	0.228	0.93	0.98				
Malignant neoplasm of ovary and other uterine adnexa	184.1	Neoplasms	0.000	0.090	0.89	1.00												
Malignant neoplasm of rectum, rectosigmoid junction,	153.3	Neoplasms	0.000	0.020	0.88	0.99	0.148	0.183	0.42	0.92	-0.823	0.276	0.00	0.12				
Malignant neoplasm of renal pelvis	189.12	Neoplasms	-0.100	0.110	0.21	0.78												
Malignant neoplasm of retroperitoneum and peritoneu	159.4	Neoplasms	0.010	0.060	0.87	0.99												
Malignant neoplasm of small intestine, including duod	159.2	Neoplasms	0.010	0.060	0.83	0.98												
Malignant neoplasm of testis	187.2	Neoplasms	0.000	0.060	0.52	0.95												
Malignant neoplasm of unspecified male genital organ	187.1	Neoplasms	-0.200	0.130	0.10	0.63												
Malignant neoplasm of uterus	182	Neoplasms	0.030	0.020	0.14	0.68	-0.084	0.216	0.70	0.94	0.047	0.163	0.77	0.98				
Malignant neoplasm, other	195.1	Neoplasms	0.020	0.020	0.38	0.94	0.048	0.199	0.81	0.97								
Manlignant and unknown neoplasms of brain and nerv	191	Neoplasms	-0.100	0.070	0.04	0.38												
Melanoma cancer		Neoplasms	-0.027	0.017	0.11	0.65												
Melanomas of skin	172.11	Neoplasms	0.000	0.010	0.29	0.86												
Monocytic leukemia	204.3	Neoplasms	0.00E+00	0.140	1.00	1.00												
Multiple myeloma	204.4	Neoplasms	0.060	0.030	0.05	0.49	0.069	0.167	0.68	0.93								
Myeloid leukemia	204.2	Neoplasms	0.000	0.170	0.96	1.00												
Myeloid leukemia, acute	204.21	Neoplasms	0.000	0.050	0.80	0.98												
Myeloid leukemia, chronic	204.22	Neoplasms	0.050	0.080	0.52	0.95												
Myeloma cancer		Neoplasms	0.018	0.039	0.64	0.97												
Myeloproliferative disease	200	Neoplasms	0.020	0.030	0.58	0.97												
Neoplasm of uncertain behavior	199	Neoplasms	0.000	0.020	0.78	0.98	0.177	0.160	0.27	0.92	0.340	0.199	0.09	0.82				
Neoplasm of uncertain behavior of breast	174.3	Neoplasms	0.060	0.090	0.48	0.95												

Neoplasm of uncertain behavior of male genital organs	187.8	Neoplasms	0.060	0.090	0.48	0.95												
Neoplasm of uncertain behavior of skin	173	Neoplasms	0.070	0.070	0.34	0.91												
Neoplasm of unspecified nature of digestive system	158	Neoplasms	0.000	0.030	0.30	0.88	0.017	0.175	0.92	0.98								
Neurofibromatosis	199.4	Neoplasms	-0.100	0.150	0.52	0.95												
Nevus, non-neoplastic	217.1	Neoplasms	0.000	0.050	0.37	0.94												
Nodular lymphoma	202.21	Neoplasms	0.000	0.050	0.26	0.82												
Non cancer		Neoplasms	0.027	0.022	0.21	0.78												
Non-Hodgkins lymphoma	202.2	Neoplasms	0.000	0.020	0.86	0.99	0.276	0.213	0.19	0.90	-0.094	0.218	0.67	0.96				
Other benign neoplasm of connective and other soft tis	215	Neoplasms	0.050	0.030	0.09	0.59	-0.108	0.189	0.57	0.92	-0.025	0.216	0.91	0.98				
Other benign neoplasm of uterus	218.2	Neoplasms	0.000	0.070	0.67	0.97												
Other non-epithelial cancer of skin	172.2	Neoplasms	0.000	0.000	0.29	0.86												
Ovarian cancer		Neoplasms	-0.022	0.029	0.45	0.95												
Pancreatic cancer	157	Neoplasms	0.000	0.030	0.53	0.95												
Pancreatic cancer		Neoplasms	-0.010	0.035	0.76	0.97												
Polycythemia vera	200.1	Neoplasms	0.020	0.050	0.63	0.97												
Prostate cancer		Neoplasms	0.008	0.011	0.47	0.95												
Secondary malignancy of bone	198.6	Neoplasms	0.000	0.020	0.84	0.99	-0.150	0.231	0.51	0.92								
Secondary malignancy of brain/spine	198.5	Neoplasms	0.000	0.040	0.27	0.83												
Secondary malignancy of lymph nodes	198.1	Neoplasms	0.000	0.030	0.83	0.98												
Secondary malignancy of respiratory organs	198.2	Neoplasms	0.000	0.020	0.97	1.00	0.126	0.199	0.53	0.92								
Secondary malignant neoplasm	198	Neoplasms	0.000	0.050	0.23	0.79												
Secondary malignant neoplasm of digestive systems	198.3	Neoplasms	0.040	0.030	0.17	0.72												
Secondary malignant neoplasm of liver	198.4	Neoplasms	0.000	0.020	0.92	1.00	-0.034	0.230	0.88	0.98								
Secondary malignant neoplasm of skin	198.7	Neoplasms	0.000	0.090	0.61	0.97												
Stomach cancer		Neoplasms	-0.005	0.042	0.91	1.00												
Testicular cancer		Neoplasms	-0.021	0.043	0.63	0.97												
Thyroid cancer	193	Neoplasms	0.080	0.040	0.09	0.61												
Thyroid cancer		Neoplasms	0.032	0.043	0.45	0.95												
Uterine cancer		Neoplasms	0.020	0.025	0.41	0.95												
Uterine leiomyoma	218.1	Neoplasms	0.000	0.010	0.70	0.97	-0.013	0.042	0.76	0.97	-0.204	0.081	0.01	0.33	0.038	0.121	0.76	0.99
Vascular hamartomas and non-neoplastic nevi	217	Neoplasms	0.080	0.130	0.52	0.95												
Abnormal involuntary movements	350.1	Neurological	0.000	0.050	0.73	0.97												
Abnormality of gait	350.2	Neurological	0.000	0.030	0.54	0.96												
Acute (transverse) myelitis	323.2	Neurological	0.050	0.110	0.66	0.97												
Acute pain	338.1	Neurological	0.140	0.060	0.03	0.36												
Anterior horn cell disease	334.2	Neurological	0.000	0.060	0.31	0.88												
Back pain		Neurological	0.002	0.005	0.64	0.97	0.018	0.029	0.54	0.92	-0.024	0.028	0.38	0.94	-0.097	0.062	0.12	0.71
Cerebral cysts	348.4	Neurological	0.000	0.130	0.80	0.98												
Cerebral degeneration, unspecified	331.9	Neurological	0.100	0.100	0.33	0.91												
Cerebral edema and compression of brain	348.2	Neurological	0.000	0.110	0.41	0.95												
Chronic pain	338.2	Neurological	-0.100	0.080	0.10	0.63												
Circadian rhythm sleep disorder	327.6	Neurological	0.150	0.200	0.43	0.95												
Coma	348.7	Neurological	0.020	0.100	0.84	0.99												
Convulsions	345.3	Neurological	0.010	0.020	0.55	0.96	-0.095	0.206	0.64	0.93	0.037	0.166	0.82	0.98				
Degenerative disease of the spinal cord	334	Neurological	0.000	0.040	0.63	0.97												
Disorders of other cranial nerves	352	Neurological	-0.100	0.170	0.32	0.89												
Disorders of the autonomic nervous system	337	Neurological	0.010	0.110	0.90	1.00												
Disturbances of sensation of smell and taste	350.6	Neurological	0.000	0.170	0.99	1.00												
Encephalitis	323	Neurological	0.000	0.060	0.68	0.97												
Encephalitis, non-infectious	323.8	Neurological	0.100	0.150	0.48	0.95												
Encephalopathy, not elsewhere classified	348.8	Neurological	0.090	0.120	0.44	0.95												
Epilepsy	345.1	Neurological	-0.100	0.070	0.13	0.67												
Epilepsy, recurrent seizures, convulsions	345	Neurological	0.000	0.030	0.94	1.00					0.153	0.203	0.45	0.94				
Essential tremor	333.1	Neurological	0.140	0.150	0.34	0.91												
Extrapyramidal disease and abnormal movement disor	333	Neurological	0.040	0.100	0.69	0.97												
Facial nerve disorders [CN7]	352.2	Neurological	0.000	0.040	0.46	0.95					0.078	0.211	0.71	0.97				
Facial pain		Neurological	-0.004	0.019	0.83	0.98												
Fluid intelligence		Neurological	-0.008	0.006	0.22	0.79	0.007	0.016	0.68	0.93	-0.014	0.015	0.35	0.94	-0.006	0.034	0.87	0.99
General pain		Neurological	0.010	0.016	0.50	0.95	-0.049	0.069	0.48	0.92	-0.012	0.060	0.85	0.98				
Generalized convulsive epilepsy	345.11	Neurological	-0.100	0.060	0.08	0.57												
Headache		Neurological	-0.007	0.006	0.30	0.87	0.011	0.044	0.79	0.97	-0.018	0.038	0.63	0.94				
Hemiplegia	342	Neurological	0.000	0.040	0.84	0.98												
Hereditary and idiopathic peripheral neuropathy	356	Neurological	0.050	0.120	0.65	0.97												
Hip pain		Neurological	0.005	0.006	0.44	0.95	0.018	0.042	0.67	0.93	-0.001	0.046	0.98	1.00				
Hydrocephalus	331.1	Neurological	0.000	0.060	0.90	1.00												
Infantile cerebral palsy	343	Neurological	0.000	0.230	0.75	0.97												
Inflammatory and toxic neuropathy	357	Neurological	0.000	0.030	0.99	1.00												
Jakob-Creutzfeldt disease	324.1	Neurological	0.010	0.220	0.96	1.00												
Knee pain		Neurological	0.008	0.005	0.11	0.64	-0.006	0.030	0.83	0.98	0.013	0.029	0.66	0.96	-0.060	0.072	0.40	0.88
Lack of coordination	350.3	Neurological	-0.100	0.090	0.19	0.75												
Meningitis	320	Neurological	0.000	0.050	0.92	1.00												
Migrain with aura	340.1	Neurological	0.020	0.070	0.78	0.98												
Migraine	340	Neurological	0.000	0.020	0.59	0.97	0.087	0.140	0.54	0.92	0.048	0.156	0.76	0.98				
Multiple sclerosis	335	Neurological	0.050	0.030	0.13	0.67												
Muscular dystrophies	359.1	Neurological	0.140	0.120	0.24	0.80												
Myasthenia gravis	358.1	Neurological	0.140	0.080	0.08	0.57												
Myoclonus	333.2	Neurological	0.000	0.190	0.76	0.97												
Myopathy	359.2	Neurological	0.060	0.060	0.33	0.91												
Neck and shoulder pain		Neurological	0.009	0.005	0.08	0.57	-0.060	0.033	0.06	0.62	-0.042	0.029	0.14	0.89	-0.083	0.062	0.18	0.73
Nerve plexus lesions	353.1	Neurological	0.100	0.100	0.31	0.88												
Nerve root and plexus disorders	353	Neurological	0.000	0.060	0.69	0.97												
Nerve root lesions	353.2	Neurological	0.170	0.140	0.21	0.78												
Nonspecific abnormal findings on radiological and oth	346.1	Neurological	-0.200	0.200	0.23	0.79												
Numeric memory		Neurological	0.002	0.007	0.77	0.97	0.150	0.086	0.08	0.66	0.012	0.046	0.79	0.98				
Organic or persistent insomnia	327.41	Neurological	0.040	0.110	0.71	0.97												
Other CNS infection and poliomyelitis	324	Neurological	0.000	0.100	0.57	0.97												
Other and unspecified disorders of the nervous system	349	Neurological	-0.100	0.070	0.09	0.61												
Other cerebral degenerations	331	Neurological	0.000	0.100	0.66	0.97												
Other conditions of brain	348	Neurological	-0.100	0.120	0.41	0.95												
Other conditions of brain, NOS	348.9	Neurological	0.000	0.060	0.99	1.00												
Other degenerative diseases of the basal ganglia	333.8	Neurological	0.020	0.150	0.87	0.99												
Other demyelinating diseases of central nervous systen	341	Neurological	0.000	0.070	0.89	1.00												
Other headache syndromes	339	Neurological	0.010	0.010	0.23	0.79	-0.078	0.073	0.28	0.92	0.044	0.068	0.51	0.94	-0.048	0.199	0.81	0.99
Other paralytic syndromes	344	Neurological	0.020	0.050	0.65	0.97												
Other peripheral nerve disorders	351	Neurological	0.000	0.000	0.40	0.94	-0.083	0.067	0.22	0.91	0.040	0.063	0.53	0.94	0.159	0.145	0.27	0.84
Parkinson's disease	332	Neurological	-0.100	0.040	0.04	0.40												
Partial epilepsy	345.12	Neurological	0.000	0.080	0.73	0.97												
Peripheral autonomic neuropathy	337.1	Neurological	0.000	0.140	0.72	0.97												
Phlebitis and thrombophlebitis of intracranial venous s	325	Neurological	-0.100	0.160	0.43	0.95												
Prospective memory		Neurological	-0.011	0.008	0.14	0.69	0.016	0.032	0.63	0.92	0.003	0.029	0.91	0.98	-0.011	0.068	0.87	0.99
Reaction time		Neurological	-0.001	0.002	0.58	0.97	-0.003	0.011	0.76	0.97	0.004	0.011	0.70	0.96	-0.001	0.022	0.96	0.99
Sleep apnea	327.3	Neurological	0.030	0.020	0.07	0.57	-0.034	0.111	0.76	0.97	-0.071	0.106	0.50	0.94				
Sleep disorders	327	Neurological	0.000	0.030	0.06	0.52					0.509	0.161	0.00	0.08				
Spinocerebellar disease	334.1	Neurological	0.180	0.110	0.11	0.65												
Stomach pain		Neurological	0.012	0.008	0.14	0.68	-0.043	0.047	0.36	0.92	-0.028	0.051	0.58	0.94				
Torsion dystonia	333.4	Neurological	0.010	0.070	0.86	0.99												
Trigeminal nerve disorders [CN5]	352.1	Neurological	0.000	0.050	0.95	1.00												
Visual memory		Neurological	0.001	0.002	0.62	0.97	-0.016	0.011	0.16	0.86	0.001	0.011	0.89	0.98	-0.009	0.021	0.67	0.98
Abnormality of organs and soft tissues of pelvis compl	654.1	Reproductive	0.000	0.040	0.40	0.94	-0.139	0.176	0.43	0.92	0.122	0.196	0.53	0.94				
Age at menarche		Reproductive	0.001	0.004	0.76	0.97	-0.026	0.030	0.40	0.92	-0.018	0.030	0.55	0.94	-0.050	0.047	0.29	0.84
Age at menopause		Reproductive	0.000	0.003	0.94	1.00	0.034	0.022	0.11	0.72	-0.006	0.021	0.79	0.98	-0.039	0.038	0.30	0.84
Age first birth		Reproductive	0.014	0.013	0.26	0.82	0.004	0.019	0.81	0.97	0.013	0.019	0.50	0.94	-0.023	0.036	0.53	0.92
Age last birth		Reproductive	0.020	0.014	0.14	0.68	0.138	0.111	0.21	0.91	-0.008	0.096	0.94	0.98	-0.287	0.180	0.11	0.71
Anemia during pregnancy	644	Reproductive	0.180	0.130	0.18	0.75												
Antepartum hemorrhage, abruptio placentae, and plac	635.2	Reproductive	0.030	0.040	0.37	0.94	0.004	0.193	0.98	1.00	0.033	0.211	0.87	0.98				
Bilateral oophorectomy		Reproductive	-5.76E-05	0.009	0.99	1.00	-0.107	0.060	0.07	0.62	-0.007	0.070	0.92	0.98				
Cervical incompetence	636.8	Reproductive	0.140	0.170	0.41	0.95												
Complications of labor and delivery NEC	669	Reproductive	0.010	0.020	0.52	0.95	0.084	0.108	0.43	0.92	-0.003	0.133	0.98	1.00				
Diabetes or abnormal glucose tolerance complicating p	649.1	Reproductive	0.070	0.100	0.49	0.95												
Early onset of delivery	636.2	Reproductive	0.040	0.070	0.55	0.96												
Early or threatened labor; hemorrhage in early pregnan	636	Reproductive	0.050	0.030	0.13	0.67	-0.035	0.145	0.81	0.97	0.189	0.183	0.30	0.93				
Ectopic pregnancy	634.3	Reproductive	0.060	0.050	0.26	0.82												
Ever had hysterectomy		Reproductive	0.009	0.009	0.36	0.93	-0.051	0.055	0.35	0.92	0.080	0.064	0.22	0.89				

Ever had stillbirth		Reproductive	0.008	0.005	0.13	0.67	-0.018	0.032	0.58	0.92	-0.024	0.035	0.49	0.94	0.034	0.057	0.55	0.92
Excessive vomiting in pregnancy	643	Reproductive	0.040	0.120	0.70	0.97												
Hemorrhage in early pregnancy	636.3	Reproductive	0.000	0.030	0.75	0.97	0.209	0.172	0.22	0.91	0.115	0.240	0.63	0.94				
Hyperemesis gravidarum	643.1	Reproductive	0.100	0.080	0.23	0.79												
Hypertension complicating pregnancy, childbirth, and	642	Reproductive	0.000	0.040	0.86	0.99	0.161	0.180	0.37	0.92	0.117	0.187	0.53	0.94				
Infections of genitourinary tract during pregnancy	647.1	Reproductive	0.090	0.080	0.29	0.86												
Infectious and parasitic complications affecting pregna	647	Reproductive	0.160	0.140	0.26	0.82												
Known or suspected fetal abnormality affecting manag	655	Reproductive	0.000	0.020	0.67	0.97	0.060	0.088	0.49	0.92	0.016	0.104	0.88	0.98	0.182	0.208	0.38	0.88
Major puerperal infection	647.3	Reproductive	-0.100	0.240	0.64	0.97												
Maternal smoking		Reproductive	0.005	0.004	0.19	0.75												
Miscarriage; stillbirth	634	Reproductive	0.020	0.010	0.19	0.76	-0.014	0.072	0.85	0.98	0.076	0.097	0.44	0.94	-0.250	0.163	0.13	0.71
Missed abortion/Hydatidiform mole	634.1	Reproductive	0.010	0.030	0.55	0.96	0.064	0.146	0.66	0.93	0.069	0.161	0.67	0.96				
Number of children fathered		Reproductive	0.000	0.002	0.94	1.00	0.013	0.017	0.43	0.92	0.016	0.014	0.27	0.93	-0.036	0.036	0.32	0.86
Other complications of pregnancy NEC	646	Reproductive	0.000	0.020	0.80	0.98	0.032	0.103	0.75	0.97	0.108	0.117	0.35	0.94				
Other complications of the puerperium NEC	674	Reproductive	0.000	0.090	0.91	1.00												
Other conditions or status of the mother complicating p	649	Reproductive	-0.300	0.240	0.15	0.69												
Other perinatal conditions of fetus or newborn	656	Reproductive	-0.100	0.140	0.46	0.95												
Placenta previa and abruptio placenta	635.3	Reproductive	0.030	0.050	0.53	0.95												
Preeclampsia and eclampsia	642.1	Reproductive	-0.100	0.070	0.06	0.51												
Problems associated with amniotic cavity and membra	653	Reproductive	0.000	0.030	0.68	0.97	0.031	0.170	0.85	0.98	0.014	0.157	0.93	0.98				
Rhesus isoimmunization in pregnancy	654.2	Reproductive	-0.100	0.130	0.17	0.72												
Venous/cerebrovascular complications embolism in pr	671	Reproductive	0.000	0.150	0.99	1.00												
Abnormal findings examination of lungs	514	Respiratory	0.000	0.020	0.75	0.97	-0.547	0.196	0.01	0.30	0.038	0.132	0.77	0.98				
Acute and chronic tonsillitis	474	Respiratory	0.000	0.050	0.91	1.00												
Acute bronchitis and bronchiolitis	483	Respiratory	0.090	0.060	0.12	0.66												
Acute laryngitis and tracheitis	465.4	Respiratory	0.000	0.090	0.76	0.97												
Acute pharyngitis	465.2	Respiratory	0.000	0.050	0.23	0.79												
Acute sinusitis	464	Respiratory	0.110	0.070	0.14	0.69												
Acute tonsillitis	474.1	Respiratory	0.000	0.040	0.49	0.95	-0.099	0.184	0.59	0.92	-0.253	0.249	0.31	0.93				
Acute upper respiratory infections of multiple or unspe	465	Respiratory	0.000	0.030	0.93	1.00	0.203	0.175	0.24	0.91	-0.108	0.200	0.59	0.94				
Allergic rhinitis	476	Respiratory	0.000	0.120	0.82	0.98												
Asthma	495	Respiratory	0.010	0.020	0.40	0.94	0.142	0.110	0.20	0.91	-0.077	0.100	0.44	0.94				
Asthma		Respiratory	0.008	0.010	0.44	0.95												
Asthma with exacerbation	495.2	Respiratory	0.040	0.060	0.47	0.95												
Bacterial pneumonia	480.1	Respiratory	0.000	0.040	0.62	0.97												
Bronchiectasis	496.3	Respiratory	0.000	0.030	0.47	0.95												
Bronchiectasis		Respiratory	-0.013	0.037	0.72	0.97												
Bronchitis	497	Respiratory	0.000	0.060	0.75	0.97												
Bronchopneumonia and lung abscess	480.5	Respiratory	-0.100	0.050	0.02	0.23												
Chronic airway obstruction	496	Respiratory	0.000	0.040	0.48	0.95												
Chronic bronchitis	496.2	Respiratory	0.040	0.120	0.73	0.97												
Chronic bronchitis		Respiratory	-0.016	0.033	0.63	0.97												
Chronic laryngitis	473.1	Respiratory	0.000	0.130	0.70	0.97												
Chronic obstructive pulmonary disease		Respiratory	-0.005	0.029	0.87	0.99												
Chronic pharyngitis and nasopharyngitis	472	Respiratory	0.060	0.050	0.23	0.79												
Chronic sinusitis	475	Respiratory	0.020	0.020	0.36	0.93	-0.073	0.222	0.74	0.97	-0.129	0.167	0.44	0.94				
Chronic tonsillitis and adenoiditis	474.2	Respiratory	0.000	0.030	0.87	0.99	0.127	0.180	0.48	0.92	0.060	0.165	0.72	0.97				
Cough	512.8	Respiratory	0.010	0.020	0.54	0.96	-0.282	0.177	0.11	0.72	-0.001	0.134	0.99	1.00				
Diseases of the larynx and vocal cords	473	Respiratory	0.000	0.020	0.64	0.97	-0.121	0.185	0.51	0.92	-0.221	0.192	0.25	0.91				
Disorders of diaphragm	513.8	Respiratory	0.000	0.130	0.82	0.98												
Emphysema	496.1	Respiratory	0.000	0.060	0.88	1.00												
Emphysema		Respiratory	0.000	0.062	1.00	1.00												
Empyema and pneumothorax	506	Respiratory	0.030	0.030	0.29	0.86												
Epistaxis or throat hemorrhage	477	Respiratory	0.060	0.020	0.00	0.02	0.127	0.173	0.46	0.92	-0.112	0.182	0.54	0.94				
Extrinsic allergic alveolitis	500.1	Respiratory	0.050	0.110	0.63	0.97												
Forced expiratory volume Z score		Respiratory	0.002	0.002	0.29	0.87												
Forced expiratory volume/forced vital capacity Z-score		Respiratory	-0.001	0.002	0.42	0.95												
Forced vital capacity Z-score		Respiratory	0.003	0.002	0.17	0.72												
Hemoptysis	516.1	Respiratory	0.010	0.020	0.61	0.97	-0.021	0.158	0.89	0.98	0.117	0.139	0.40	0.94				
Hyperventilation	513.4	Respiratory	0.000	0.090	0.56	0.97												
Hypoventilation	513.3	Respiratory	0.210	0.170	0.22	0.78												
Influenza	481	Respiratory	0.010	0.030	0.66	0.97					-0.332	0.208	0.11	0.88				
Lung disease due to external agents	500	Respiratory	0.010	0.130	0.93	1.00												
Nasal polyps	471	Respiratory	0.000	0.020	0.48	0.95	-0.074	0.216	0.73	0.96	-0.124	0.168	0.46	0.94				
Obstructive chronic bronchitis	496.21	Respiratory	0.000	0.010	0.95	1.00					0.014	0.171	0.94	0.98				
Other alveolar and parietoalveolar pneumonopathy	504	Respiratory	0.040	0.050	0.49	0.95												
Other diseases of lung	510	Respiratory	0.090	0.040	0.03	0.30												
Other diseases of respiratory system, NEC	519.8	Respiratory	0.040	0.010	0.00	0.01	-0.132	0.102	0.19	0.90	-0.017	0.076	0.82	0.98				
Other diseases of respiratory system, not elsewhere cla	519	Respiratory	-0.400	0.220	0.05	0.46												
Other dyspnea	512.9	Respiratory	0.000	0.040	0.95	1.00												
Other pulmonary inflamation or edema	505	Respiratory	0.000	0.110	0.73	0.97												
Other upper respiratory disease	479	Respiratory	0.000	0.020	0.00	0.03	-0.319	0.151	0.03	0.54	-0.145	0.129	0.26	0.92	-0.320	0.266	0.23	0.84
Painful respiration	512.2	Respiratory	0.030	0.060	0.64	0.97												
Paralysis/spasm of vocal cords or larynx	473.3	Respiratory	0.070	0.070	0.29	0.86												
Pleurisy; pleural effusion	507	Respiratory	0.010	0.020	0.51	0.95	-0.113	0.161	0.48	0.92	0.404	0.125	0.00	0.07				
Pneumococcal pneumonia	480.11	Respiratory	0.000	0.010	0.59	0.97	-0.019	0.093	0.84	0.98	0.106	0.084	0.20	0.89				
Pneumoconiosis	500.2	Respiratory	-0.200	0.210	0.20	0.77												
Pneumonia	480	Respiratory	0.000	0.010	0.72	0.97	0.095	0.102	0.35	0.92	-0.169	0.096	0.08	0.82				
Pneumonia due to fungus (mycoses)	480.3	Respiratory	0.010	0.100	0.92	1.00												
Pneumonitis due to inhalation of food or vomitus	501	Respiratory	0.000	0.040	0.04	0.38												
Postinflammatory pulmonary fibrosis	502	Respiratory	0.000	0.040	0.91	1.00					-0.092	0.189	0.63	0.94				
Pseudomonal pneumonia	480.12	Respiratory	0.050	0.100	0.61	0.97												
Pulmonary collapse; interstitial and compensatory emp	508	Respiratory	0.080	0.060	0.18	0.75												
Pulmonary congestion and hypostasis	503	Respiratory	0.010	0.090	0.89	1.00												
Respiratory complications	519.2	Respiratory	0.020	0.110	0.82	0.98												
Respiratory failure	509.1	Respiratory	0.000	0.040	0.50	0.95												
Respiratory insufficiency	509.2	Respiratory	0.000	0.050	0.26	0.82												
Sarcoidosis		Respiratory	-0.026	0.054	0.62	0.97												
Septal Deviations/Turbinate Hypertrophy	470	Respiratory	0.000	0.010	0.53	0.95	0.041	0.178	0.82	0.97	-0.062	0.096	0.52	0.94				
ShortBreath		Respiratory	0.026	0.010	0.01	0.13	-0.080	0.043	0.06	0.62	0.012	0.035	0.73	0.98				
Shortness of breath	512.7	Respiratory	0.030	0.010	0.04	0.38	0.013	0.101	0.90	0.98	0.123	0.088	0.16	0.89				
Symptoms involving respiratory system and other ches	519.9	Respiratory	0.000	0.060	0.88	1.00												
Throat pain	478	Respiratory	0.100	0.050	0.05	0.46												
Viral pneumonia	480.2	Respiratory	0.000	0.090	0.57	0.97												
Voice disturbance	473.4	Respiratory	0.030	0.030	0.43	0.95												
Wheezing	512.1	Respiratory	0.100	0.130	0.43	0.95												
Amblyopia		Sense Organs	1.34E-04	0.035	1.00	1.00												
Aphakia and other disorders of lens	379.3	Sense Organs	0.000	0.010	0.55	0.96	0.069	0.128	0.59	0.92	-0.100	0.108	0.35	0.94	-0.090	0.275	0.74	0.99
Astigmatism	367.2	Sense Organs	0.000	0.120	0.63	0.97												
Astigmatism		Sense Organs	-0.027	0.026	0.31	0.88												
Average mean spherical equivalent refractive error		Sense Organs	-0.001	0.004	0.80	0.98	-0.002	0.016	0.90	0.98	0.005	0.017	0.74	0.98	-0.062	0.036	0.09	0.70
Blindness and low vision	367.9	Sense Organs	0.000	0.120	0.64	0.97												
Cataract	366	Sense Organs	0.000	0.000	0.59	0.97	0.051	0.044	0.24	0.91	-0.042	0.037	0.26	0.92	-0.076	0.092	0.41	0.88
Cataract		Sense Organs	0.009	0.012	0.46	0.95	0.097	0.064	0.13	0.78	-0.012	0.062	0.85	0.98				
Cataract surgery		Sense Organs	-0.024	0.027	0.37	0.93												
Cholesteatoma	385.3	Sense Organs	0.020	0.040	0.60	0.97												
Chorioretinal inflammations, scars, and other disorders	363	Sense Organs	0.150	0.120	0.21	0.77												
Conductive hearing loss	389.2	Sense Organs	0.030	0.060	0.56	0.97												
Conjunctivitis, infectious	369.5	Sense Organs	0.000	0.160	0.99	1.00												
Conjunctivitis, noninfectious	371.2	Sense Organs	0.180	0.160	0.28	0.86												
Corneal degenerations	364.4	Sense Organs	0.000	0.120	0.90	1.00												
Corneal dystrophy	364.5	Sense Organs	0.000	0.070	0.38	0.94												
Corneal edema	364.2	Sense Organs	0.000	0.140	0.56	0.97												
Corneal hysteresis, left eye		Sense Organs	-0.005	0.004	0.22	0.78	-0.012	0.017	0.49	0.92	-0.019	0.017	0.27	0.93	-0.134	0.038	0.00	0.08
Corneal hysteresis, right eye		Sense Organs	-0.003	0.004	0.45	0.95	-0.013	0.017	0.43	0.92	0.001	0.017	0.96	0.98	-0.119	0.038	0.00	0.14
Corneal opacity	364.1	Sense Organs	0.120	0.170	0.46	0.95												
Corneal opacity and other disorders of cornea	364	Sense Organs	0.000	0.080	0.58	0.97												
Corneal ulcer	370.1	Sense Organs	0.020	0.080	0.75	0.97												
Diabetic eye disease		Sense Organs	-0.016	0.024	0.51	0.95					-0.015	0.069	0.83	0.98				
Diplopia and disorders of binocular vision	368.2	Sense Organs	0.090	0.060	0.14	0.68												
Disorders of conjunctiva	372	Sense Organs	0.000	0.040	0.18	0.75	0.007	0.116	0.95	0.99	-0.343	0.192	0.07	0.82				
Disorders of external ear	380	Sense Organs	0.000	0.040	0.81	0.98												
Disorders of iris and ciliary body	379.5	Sense Organs	0.120	0.090	0.20	0.76												

Disorders of lacrimal system	375	Sense Organs	0.020	0.020	0.28	0.86	0.065	0.172	0.71	0.94	-0.204	0.129	0.11	0.89				
Disorders of optic nerve and visual pathways	377	Sense Organs	-0.100	0.160	0.50	0.95												
Disorders of refraction and accommodation; blindness	367	Sense Organs	0.000	0.140	0.89	1.00												
Disorders of the globe	360	Sense Organs	-0.200	0.220	0.21	0.78												
Disorders of the orbit	376	Sense Organs	0.000	0.110	0.77	0.97												
Disorders of vitreous body	379.2	Sense Organs	0.010	0.030	0.75	0.97	0.122	0.222	0.58	0.92								
Dizziness and giddiness (Light-headedness and vertigo	386.9	Sense Organs	0.040	0.010	0.01	0.18	-0.053	0.114	0.64	0.93	-0.091	0.104	0.38	0.94				
Ectropion or entropion	374.1	Sense Organs	0.000	0.020	0.30	0.87												
Epiphora	375.2	Sense Organs	0.000	0.030	0.93	1.00	0.150	0.211	0.48	0.92								
Eustachian tube disorders	381.2	Sense Organs	0.010	0.070	0.80	0.98												
Eye infection, viral	369.2	Sense Organs	0.030	0.100	0.72	0.97												
Eye surgery		Sense Organs	-0.005	0.014	0.73	0.97	-0.037	0.067	0.58	0.92	-0.044	0.056	0.43	0.94				
Glaucoma	365	Sense Organs	0.000	0.020	0.62	0.97	-0.240	0.143	0.09	0.66	0.194	0.149	0.19	0.89				
Glaucoma		Sense Organs	0.001	0.016	0.96	1.00												
Hearing aid		Sense Organs	0.000	0.011	0.98	1.00												
Hearing loss	389	Sense Organs	0.000	0.040	0.03	0.38												
Hypermetropia	367.8	Sense Organs	-0.100	0.150	0.25	0.82												
Hypermetropia		Sense Organs	0.000	0.010	0.98	1.00	0.026	0.043	0.55	0.92	-0.003	0.044	0.95	0.98				
Impacted cerumen	380.4	Sense Organs	-0.100	0.080	0.19	0.76												
Infection of the eye	369	Sense Organs	0.000	0.070	0.21	0.78												
Inflammation of eyelids	371.3	Sense Organs	0.000	0.020	0.15	0.70	0.064	0.147	0.66	0.93	-0.132	0.121	0.28	0.93				
Inflammation of the eye	371	Sense Organs	0.160	0.120	0.18	0.75												
Keratitis	370	Sense Organs	0.000	0.110	0.94	1.00												
Keratoconus	364.41	Sense Organs	0.000	0.120	0.93	1.00												
Labyrinthitis	386.3	Sense Organs	0.060	0.030	0.07	0.57	0.113	0.220	0.61	0.92	0.287	0.210	0.17	0.89				
Lagophthalmos	374.2	Sense Organs	0.170	0.190	0.37	0.94												
Laser eye surgery		Sense Organs	-0.008	0.027	0.76	0.97												
Macular degeneration		Sense Organs	0.005	0.023	0.82	0.98												
Macular degeneration (senile) of retina NOS	362.29	Sense Organs	0.010	0.020	0.66	0.97	-0.120	0.142	0.40	0.92	-0.003	0.142	0.98	1.00				
Mastoiditis & related conditions	381.3	Sense Organs	0.070	0.090	0.39	0.94												
Mean intraoccular pressure, Goldmann-correlated		Sense Organs	-0.007	0.004	0.06	0.53	0.037	0.017	0.03	0.49	-0.031	0.017	0.07	0.82	-0.002	0.037	0.95	0.99
Mean intraoccular pressure, corneal-compensated		Sense Organs	-0.005	0.004	0.24	0.80	0.042	0.017	0.01	0.34	-0.026	0.017	0.12	0.89	0.092	0.037	0.01	0.50
Meniere's disease	386.1	Sense Organs	0.090	0.070	0.21	0.77												
Myopia	367.1	Sense Organs	0.080	0.110	0.44	0.95												
Myopia		Sense Organs	-0.003	0.008	0.72	0.97	-0.021	0.040	0.59	0.92	-0.058	0.038	0.13	0.89	0.094	0.087	0.28	0.84
Nonsenile Cataract	366.1	Sense Organs	0.010	0.120	0.89	1.00												
Optic neuritis/neuropathy	377.3	Sense Organs	0.080	0.090	0.39	0.94												
Otalgia	382	Sense Organs	0.020	0.070	0.72	0.97												
Other disorders of ear	388	Sense Organs	0.000	0.120	0.77	0.97												
Other disorders of eye	379	Sense Organs	0.010	0.070	0.86	0.99												
Other disorders of eyelids	374	Sense Organs	0.000	0.010	0.88	1.00	-0.013	0.158	0.93	0.98	0.016	0.123	0.89	0.98				
Other disorders of middle ear and mastoid	385	Sense Organs	0.000	0.100	0.45	0.95												
Other disorders of tympanic membrane	384	Sense Organs	0.030	0.070	0.69	0.97												
Other nondiabetic retinopathy	362.3	Sense Organs	0.100	0.160	0.50	0.95												
Other retinal disorders	362	Sense Organs	0.000	0.030	0.97	1.00	-0.367	0.241	0.13	0.77	0.099	0.147	0.50	0.94				
Otitis externa	380.1	Sense Organs	0.020	0.040	0.64	0.97												
Otitis media	381.1	Sense Organs	0.020	0.030	0.35	0.92					-0.085	0.185	0.65	0.95				
Otorrhea	381.9	Sense Organs	0.000	0.080	0.29	0.86												
Otosclerosis	383	Sense Organs	0.000	0.060	0.95	1.00												
Pain, swelling or discharge of eye	379.9	Sense Organs	0.040	0.100	0.64	0.97												
Paralytic strabismus	378.5	Sense Organs	0.030	0.070	0.64	0.97												
Perforation of tympanic membrane	384.4	Sense Organs	0.000	0.030	0.41	0.95					-0.144	0.165	0.38	0.94				
Peripheral or central vertigo	386.2	Sense Organs	0.050	0.040	0.27	0.83												
Peripheral retinal degenerations	362.6	Sense Organs	0.030	0.150	0.80	0.98												
Presbyopia		Sense Organs	0.005	0.008	0.56	0.97	0.023	0.041	0.57	0.92	0.051	0.041	0.22	0.89				
Primary angle-closure glaucoma	365.2	Sense Organs	0.000	0.030	0.93	1.00												
Primary open angle glaucoma	365.11	Sense Organs	0.000	0.030	0.93	1.00	-0.015	0.124	0.90	0.98	-0.152	0.184	0.41	0.94				
Ptosis of eyelid	374.3	Sense Organs	0.000	0.020	0.83	0.98	-0.077	0.168	0.65	0.93	0.030	0.159	0.85	0.98				
Retinal detachment with retinal defect	361.1	Sense Organs	0.000	0.020	0.05	0.49	0.187	0.206	0.36	0.92	0.086	0.171	0.61	0.94				
Retinal detachments and defects	361	Sense Organs	0.000	0.020	0.74	0.97	0.005	0.204	0.98	1.00	-0.213	0.196	0.28	0.93				
Retinal hemorrhage/ischemia	362.8	Sense Organs	0.090	0.150	0.52	0.95												
Retinal vascular changes and abnomalities	362.4	Sense Organs	0.010	0.030	0.74	0.97												
Retinoschisis and retinal cysts	361.2	Sense Organs	0.070	0.190	0.70	0.97												
Senile cataract	366.2	Sense Organs	0.010	0.000	0.11	0.65	0.053	0.058	0.36	0.92	-0.028	0.044	0.53	0.94	-0.017	0.114	0.88	0.99
Sensorineural hearing loss	389.1	Sense Organs	0.060	0.070	0.36	0.93												
Separation of retinal layers	362.31	Sense Organs	0.110	0.120	0.34	0.92												
Speech-reception-threshold estimate (left)		Sense Organs	0.001	0.003	0.79	0.98	-0.016	0.015	0.29	0.92	0.053	0.015	2.52E-04	0.04	-0.020	0.033	0.53	0.92
Speech-reception-threshold estimate (right)		Sense Organs	0.002	0.003	0.52	0.95	-0.018	0.015	0.25	0.91	0.052	0.015	3.74E-04	0.04	-0.057	0.034	0.09	0.70
Strabismus		Sense Organs	-0.031	0.044	0.49	0.95												
Strabismus (not specified as paralytic)	378.1	Sense Organs	0.060	0.030	0.08	0.57												
Strabismus and other disorders of binocular eye mover	378	Sense Organs	0.000	0.150	0.92	1.00												
Subjective visual disturbances	368.9	Sense Organs	0.190	0.070	0.01	0.12												
Suppurative and unspecified otitis media	381.11	Sense Organs	0.000	0.040	0.28	0.86					-0.101	0.230	0.66	0.96				
Tinnitus	389.4	Sense Organs	-0.200	0.130	0.04	0.39												
Tinnitus		Sense Organs	-0.004	0.014	0.75	0.97	-0.012	0.069	0.86	0.98	-0.006	0.057	0.91	0.98				
Traumatic cataract	366.3	Sense Organs	0.020	0.100	0.83	0.98												
Tympanosclerosis and middle ear disease related to oti	385.5	Sense Organs	0.000	0.100	0.71	0.97												
Uveitis, noninfectious or NOS	371.1	Sense Organs	0.040	0.080	0.59	0.97												
Vertiginous syndromes and other disorders of vestibul	386	Sense Organs	0.160	0.110	0.14	0.68												
Visual acuity, left eye		Sense Organs	-0.003	0.004	0.43	0.95	-0.001	0.016	0.95	0.99	-0.002	0.016	0.89	0.98	-0.001	0.035	0.97	0.99
Visual acuity, right eye		Sense Organs	-0.001	0.004	0.71	0.97	-0.013	0.016	0.42	0.92	-0.006	0.016	0.69	0.96	-0.002	0.035	0.95	0.99
Visual disturbances	368	Sense Organs	0.000	0.040	0.85	0.99												
Visual field defects	368.4	Sense Organs	0.060	0.100	0.51	0.95												
Abdominal pain	785	Symptoms	0.000	0.000	0.96	1.00	0.014	0.035	0.70	0.94	-0.012	0.035	0.72	0.97	-0.108	0.077	0.16	0.73
Back pain	760	Symptoms	0.000	0.010	0.26	0.83	0.005	0.060	0.93	0.98	0.007	0.061	0.91	0.98	0.430	0.196	0.03	0.66
Cervical radiculitis	765	Symptoms	0.000	0.040	0.78	0.98												
Cervicalgia	761	Symptoms	0.000	0.020	0.39	0.94	-0.344	0.176	0.05	0.62	-0.137	0.140	0.33	0.94				
Chronic fatigue syndrome	798.1	Symptoms	-0.300	0.170	0.05	0.44												
Edema	782.3	Symptoms	0.020	0.040	0.62	0.97												
Fever of unknown origin	783	Symptoms	0.050	0.020	0.02	0.30	-0.338	0.157	0.03	0.51	-0.112	0.100	0.26	0.93				
Gangrene	791	Symptoms	-0.100	0.100	0.08	0.59												
Hypothermia/Chills	780	Symptoms	0.150	0.120	0.19	0.76												
Malaise and fatigue	798	Symptoms	0.010	0.020	0.50	0.95	0.058	0.148	0.69	0.94	0.028	0.169	0.87	0.98				
Muscular wasting and disuse atrophy	772.1	Symptoms	0.280	0.160	0.09	0.61												
Musculoskeletal symptoms referable to limbs	771	Symptoms	0.000	0.040	0.35	0.92	0.229	0.204	0.26	0.92								
Myalgia and myositis unspecified	770	Symptoms	0.040	0.040	0.32	0.89	0.131	0.205	0.52	0.92								
Nausea and vomiting	789	Symptoms	0.000	0.010	0.96	1.00	-0.015	0.103	0.88	0.98	-0.019	0.109	0.86	0.98				
Neuralgia, neuritis, and radiculitis NOS	766	Symptoms	0.020	0.020	0.39	0.94	-0.016	0.157	0.92	0.98	-0.080	0.151	0.60	0.94				
Nonspecific findings on examination of blood	790	Symptoms	0.060	0.150	0.67	0.97												
Other abnormal blood chemistry	790.6	Symptoms	0.010	0.010	0.23	0.79	0.136	0.098	0.16	0.86	0.192	0.119	0.11	0.87				
Pallor and flushing	782.6	Symptoms	0.000	0.150	0.88	1.00												
Rhabdomyolysis	772.4	Symptoms	-0.300	0.260	0.25	0.81												
Sciatica	764	Symptoms	0.000	0.020	0.08	0.57	-0.080	0.144	0.58	0.92	-0.408	0.166	0.01	0.33				
Swelling of limb	771.1	Symptoms	0.000	0.010	0.66	0.97	0.057	0.091	0.53	0.92	-0.108	0.092	0.24	0.90				
Symptoms involving nervous and musculoskeletal syst	781	Symptoms	0.040	0.020	0.11	0.65	0.151	0.176	0.39	0.92	-0.218	0.168	0.20	0.89				
Symptoms of the muscles	772	Symptoms	0.000	0.050	0.39	0.94												
Syncope and collapse	788	Symptoms	0.000	0.010	0.83	0.98	0.099	0.081	0.22	0.91	0.094	0.068	0.17	0.89	-0.023	0.209	0.91	0.99

Supplemental Table 5. Lp(a) PRS-phenotye assocations study results using high Lp(a) defined based on the percentile of the PRS corresponding to the Lp(a) level ≥125nmol/L in Europeans and Africans. Cells are empty when there are fewer than 20 cases.

			European				African			
**Phenotype**	**Phecode**	**Class**	Estimate	SE	P	FDR	Estimate	SE	P	FDR
Abdominal aortic aneurysm	442.11	Circulatory System	0.273	0.085	0.001	0.037				
Abnormal function study of cardiovascular system	429.2	Circulatory System	-0.195	0.231	0.398	0.906				
Abnormal heart sounds	396	Circulatory System	-0.225	0.551	0.683	0.977				
Acute pericarditis	420.21	Circulatory System	0.256	0.173	0.140	0.657				
Acute vascular insufficiency of intestine	441.1	Circulatory System	-0.275	0.188	0.143	0.666				
Age angina diagnosed		Circulatory System	-0.658	0.235	0.005	0.101				
Age deep vein thrombosis diagnosed		Circulatory System	0.321	0.391	0.411	0.917				
Age pulmonary embolism diagnosed		Circulatory System	0.082	0.052	0.116	0.626				
Aneurysm and dissection of heart	411.41	Circulatory System	-0.038	0.154	0.807	0.979				
Aneurysm of artery of lower extremity	442.3	Circulatory System	0.307	0.180	0.089	0.594				
Aneurysm of iliac artery	442.2	Circulatory System	0.190	0.308	0.536	0.935				
Aneurysm of other specified artery	442.8	Circulatory System	-0.043	0.198	0.829	0.979				
Angina		Circulatory System	0.111	0.018	8.26E-10	5.79E-08	-0.033	0.093	0.722	0.991
Angina pectoris	411.3	Circulatory System	0.263	0.031	1.25E-17	1.41E-15	-0.143	0.282	0.611	0.980
Angina, grade 1		Circulatory System	0.097	0.024	5.512E-05	0.002	-0.030	0.139	0.829	0.991
Angina, grade 2		Circulatory System	0.129	0.026	9.91E-07	5.399E-05	-0.035	0.119	0.770	0.991
Anomalous atrioventricular excitation	426.4	Circulatory System	-0.441	0.280	0.115	0.626				
Aortic aneurysm	442.1	Circulatory System	-0.035	0.137	0.797	0.979				
Aortic valve disease	394.3	Circulatory System	0.359	0.089	5.472E-05	0.002				
Arrhythmia (cardiac) NOS	427.5	Circulatory System	-0.075	0.128	0.557	0.944				
Arterial dissection	442.4	Circulatory System	0.571	0.429	0.183	0.720				
Arterial embolism and thrombosis	444	Circulatory System	0.171	0.135	0.206	0.754				
Arterial embolism and thrombosis of lower extremity artery	444.1	Circulatory System	0.303	0.103	0.003	0.076				
Arterial stiffness index		Circulatory System	-0.011	0.008	0.176	0.718	-0.033	0.042	0.430	0.949
Arteritis NOS	446.9	Circulatory System	0.219	0.185	0.236	0.787				
Atherosclerosis	440	Circulatory System	0.377	0.208	0.070	0.545				
Atherosclerosis of aorta	440.9	Circulatory System	0.488	0.245	0.046	0.464				
Atherosclerosis of renal artery	440.1	Circulatory System	0.226	0.320	0.479	0.925				
Atherosclerosis of the extremities	440.2	Circulatory System	0.455	0.078	5.57E-09	3.72E-07				
Atrial fibrillation and flutter	427.2	Circulatory System	0.051	0.025	0.042	0.453	-0.996	0.400	0.013	0.384
Atrioventricular [AV] block	426.2	Circulatory System	-0.105	0.298	0.726	0.977				
Atrioventricular block, complete	426.24	Circulatory System	-0.175	0.106	0.097	0.605				
Bundle branch block	426.3	Circulatory System	-0.091	0.215	0.672	0.977				
Cardiac arrest	427.42	Circulatory System	0.351	0.123	0.004	0.094				
Cardiac conduction disorders	426	Circulatory System	0.631	0.298	0.034	0.393				
Cardiac index		Circulatory System	-0.012	0.015	0.430	0.919	-0.014	0.184	0.938	0.995
Cardiac output		Circulatory System	-0.012	0.014	0.404	0.910	-0.029	0.172	0.864	0.991
Cardiomegaly	416	Circulatory System	0.110	0.208	0.598	0.964				
Cerebral aneurysm	433.5	Circulatory System	-0.179	0.157	0.253	0.806				
Cerebral artery occlusion, with cerebral infarction	433.21	Circulatory System	0.171	0.047	0.000	0.008	-0.721	0.359	0.044	0.706
Cerebral atherosclerosis	433.12	Circulatory System	0.130	0.466	0.780	0.979				
Cerebral ischemia	433.3	Circulatory System	-0.097	0.244	0.692	0.977				
Cerebrovascular disease	433	Circulatory System	0.137	0.099	0.167	0.711				
Chronic pericarditis	420.22	Circulatory System	0.628	0.370	0.089	0.594				
Chronic pulmonary heart disease	415.2	Circulatory System	0.524	0.322	0.104	0.621				
Chronic vascular insufficiency of intestine	441.2	Circulatory System	0.313	0.369	0.396	0.906				
Chronic venous insufficiency [CVI]	456	Circulatory System	0.249	0.219	0.254	0.807				
Circulatory disease NEC	459.9	Circulatory System	-0.203	0.198	0.307	0.862				
Congestive heart failure (CHF) NOS	428.1	Circulatory System	0.150	0.060	0.013	0.194	0.199	0.367	0.587	0.973
Coronary atherosclerosis	411.4	Circulatory System	0.413	0.019	3.92E-108	1.44E-105	0.099	0.186	0.594	0.974
Definite claudication		Circulatory System	0.152	0.069	0.028	0.337	-0.052	0.305	0.865	0.991
Disease of capillaries	448	Circulatory System	-0.202	0.333	0.544	0.935				
Disease of tricuspid valve	394.7	Circulatory System	-0.255	0.171	0.137	0.653				
Endocarditis	420.3	Circulatory System	0.118	0.133	0.374	0.906				
Essential hypertension	401.1	Circulatory System	0.040	0.064	0.530	0.935	-0.304	0.297	0.306	0.941
First degree AV block	426.21	Circulatory System	0.253	0.149	0.088	0.594				
Frontal angle P-wave		Circulatory System	0.017	0.027	0.528	0.935				
Frontal angle QRS		Circulatory System	0.000	0.027	0.989	0.998				
Frontal angle T-Wave		Circulatory System	0.175	0.551	0.751	0.979				
Giant cell arteritis	446.5	Circulatory System	0.035	0.124	0.776	0.979				
Heart failure NOS	428.2	Circulatory System	0.250	0.059	2.144E-05	0.001	-0.130	0.398	0.745	0.991
Hemorrhage NOS	459.1	Circulatory System	-0.561	0.480	0.242	0.790				
Hemorrhoids	455	Circulatory System	0.015	0.022	0.484	0.925	0.147	0.132	0.263	0.916
Hypersensitivity angiitis	446.3	Circulatory System	0.154	0.434	0.723	0.977				
Hypertension		Circulatory System	0.043	0.010	1.329E-05	0.001	0.027	0.064	0.676	0.991
Hypertensive chronic kidney disease	401.22	Circulatory System	0.248	0.139	0.074	0.558				
Hypertensive heart disease	401.21	Circulatory System	0.752	0.302	0.013	0.193				
Hypertrophic obstructive cardiomyopathy	425.11	Circulatory System	-0.616	0.356	0.084	0.587				
Hypotension	458	Circulatory System	0.085	0.464	0.854	0.986				
Hypotension NOS	458.9	Circulatory System	0.272	0.119	0.023	0.301				
Iatrogenic hypotension	458.2	Circulatory System	0.185	0.195	0.340	0.883				
Intracerebral hemorrhage	430.2	Circulatory System	0.034	0.082	0.681	0.977	-1.089	0.747	0.145	0.796
Intracranial hemorrhage	430	Circulatory System	0.120	0.178	0.500	0.927				
Left bundle branch block	426.32	Circulatory System	0.119	0.170	0.483	0.925				
Left ventricle end diastolic volume		Circulatory System	-0.007	0.013	0.559	0.944	0.047	0.168	0.780	0.991
Left ventricle end systolic volume		Circulatory System	-0.003	0.013	0.845	0.983	0.058	0.170	0.735	0.991
Left ventricle stroke volume		Circulatory System	-0.014	0.013	0.284	0.833	0.133	0.166	0.426	0.949
Left ventricular ejection fraction		Circulatory System	-0.011	0.015	0.457	0.925	0.016	0.182	0.930	0.995
Maximum carotid IMT		Circulatory System	-0.004	0.013	0.774	0.979	0.025	0.150	0.868	0.991
Mean carotid IMT		Circulatory System	-0.013	0.013	0.324	0.874	0.039	0.150	0.797	0.991
Mean heart rate		Circulatory System	0.005	0.015	0.722	0.977	-0.111	0.185	0.548	0.967
Mitral valve disease	394.2	Circulatory System	-0.095	0.081	0.241	0.790				
Myocardial infarction	411.2	Circulatory System	0.391	0.024	1.41E-60	3.45E-58	0.417	0.228	0.067	0.706
Myocarditis	420.1	Circulatory System	0.157	0.256	0.540	0.935				
Noninfectious disorders of lymphatic channels	450	Circulatory System	-0.091	0.164	0.578	0.955				
Nonrheumatic aortic valve disorders	395.2	Circulatory System	0.432	0.238	0.069	0.545				
Nonrheumatic mitral valve disorders	395.1	Circulatory System	-0.116	0.084	0.167	0.711				
Nonspecific chest pain	418	Circulatory System	0.065	0.015	2.675E-05	0.001	-0.134	0.093	0.150	0.796
Occlusion and stenosis of precerebral arteries	433.1	Circulatory System	0.578	0.082	1.80E-12	1.47E-10				
Occlusion of cerebral arteries	433.2	Circulatory System	0.077	0.038	0.043	0.453	-0.180	0.246	0.463	0.964
Occlusion of cerebral arteries, with cerebral infarction	433.11	Circulatory System	0.161	0.167	0.335	0.879				
Orthostatic hypotension	458.1	Circulatory System	0.135	0.074	0.070	0.545	0.084	0.472	0.859	0.991
Other acute and subacute forms of ischemic heart disease	411.9	Circulatory System	0.203	0.074	0.006	0.113				
Other cardiomyopathy	425.8	Circulatory System	-0.150	0.498	0.763	0.979				
Other chronic ischemic heart disease, unspecified	411.8	Circulatory System	0.359	0.050	8.13E-13	7.47E-11	0.607	0.380	0.110	0.766
Other disorders of arteries and arterioles	447	Circulatory System	0.176	0.121	0.144	0.670				
Other disorders of circulatory system	459	Circulatory System	0.236	0.265	0.373	0.906				
Other forms of chronic heart disease	414	Circulatory System	0.096	0.131	0.465	0.925				
Other heart block	426.25	Circulatory System	0.096	0.268	0.721	0.977				
Other hypertrophic cardiomyopathy	425.12	Circulatory System	-0.381	0.302	0.207	0.754				
Other specified cardiac dysrhythmias	427.3	Circulatory System	0.101	0.072	0.158	0.701				
Other specified peripheral vascular diseases	443.8	Circulatory System	0.549	0.458	0.230	0.787				
Other venous embolism and thrombosis	452	Circulatory System	-0.025	0.154	0.872	0.987				
P-duration (from P-onset to P-offset)		Circulatory System	0.072	0.331	0.827	0.979				
PQ duration (from P-onset to QRS-onset)		Circulatory System	0.030	0.027	0.266	0.813				

Palpitations	427.9	Circulatory System	0.073	0.049	0.136	0.650	-0.003	0.305	0.991	0.996
Paroxysmal supraventricular tachycardia	427.11	Circulatory System	0.018	0.058	0.755	0.979	0.131	0.438	0.765	0.991
Paroxysmal tachycardia, unspecified	427.1	Circulatory System	-0.338	0.329	0.304	0.862				
Paroxysmal ventricular tachycardia	427.12	Circulatory System	0.120	0.096	0.211	0.761				
Pericarditis	420.2	Circulatory System	0.194	0.094	0.040	0.439	0.132	0.481	0.784	0.991
Peripheral angiopathy in diseases classified elsewhere	443.7	Circulatory System	-0.012	0.155	0.937	0.990				
Peripheral vascular disease, unspecified	443.9	Circulatory System	0.332	0.080	0.000	0.001				
Phlebitis and thrombophlebitis	451	Circulatory System	-0.047	0.169	0.780	0.979				
Phlebitis and thrombophlebitis of lower extremities	451.2	Circulatory System	-0.065	0.044	0.139	0.656	0.417	0.277	0.132	0.787
Precordial pain	418.1	Circulatory System	0.080	0.036	0.027	0.332	-0.083	0.220	0.704	0.991
Premature beats	427.6	Circulatory System	-0.018	0.150	0.904	0.987				
Primary pulmonary hypertension	415.21	Circulatory System	0.105	0.254	0.678	0.977				
Primary/intrinsic cardiomyopathies	425.1	Circulatory System	-0.028	0.101	0.779	0.979	-0.130	0.565	0.818	0.991
Pulmonary embolism and infarction, acute	415.11	Circulatory System	0.038	0.274	0.888	0.987				
Pulmonary heart disease	415	Circulatory System	0.013	0.041	0.750	0.979	-0.069	0.274	0.802	0.991
QRS duration		Circulatory System	-0.006	0.019	0.777	0.979				
QT interval (Bazett heart rate corrected)		Circulatory System	0.020	0.535	0.970	0.994				
RR interval		Circulatory System	0.057	3.390	0.987	0.997				
Raynaud's syndrome	443.1	Circulatory System	0.399	0.219	0.068	0.545				
Rheumatic disease of the heart valves	394	Circulatory System	0.227	0.112	0.043	0.453				
Right bundle branch block	426.31	Circulatory System	0.185	0.257	0.472	0.925				
Second degree AV block	426.23	Circulatory System	0.115	0.120	0.340	0.883				
Self-reported deep vein thrombosis		Circulatory System	0.014	0.031	0.641	0.968	0.019	0.219	0.931	0.995
Self-reported pulmonary embolism		Circulatory System	0.009	0.059	0.883	0.987	0.088	0.342	0.797	0.991
Self-reported venous thromboembolism		Circulatory System	0.013	0.028	0.629	0.965	0.040	0.186	0.830	0.991
Sinoatrial node dysfunction (Bradycardia)	427.8	Circulatory System	-0.189	0.122	0.122	0.646				
Stricture of artery	447.1	Circulatory System	0.557	0.081	7.31E-12	5.66E-10				
Subarachnoid hemorrhage	430.1	Circulatory System	-0.011	0.086	0.897	0.987	-0.234	0.557	0.674	0.991
Subdural hemorrhage	430.3	Circulatory System	-0.053	0.138	0.698	0.977				
Supraventricular premature beats	427.61	Circulatory System	-0.077	0.394	0.845	0.983				
Symptoms involving cardiovascular system	429.3	Circulatory System	-0.229	0.246	0.352	0.896				
Tachycardia NOS	427.7	Circulatory System	0.057	0.126	0.652	0.973				
Transient cerebral ischemia	433.31	Circulatory System	0.130	0.050	0.009	0.150	0.306	0.395	0.439	0.949
Unstable angina (intermediate coronary syndrome)	411.1	Circulatory System	0.347	0.037	4.82E-21	7.09E-19	-0.052	0.294	0.858	0.991
Varicose veins	454	Circulatory System	0.138	0.142	0.332	0.876				
Varicose veins of lower extremity	454.1	Circulatory System	-0.024	0.026	0.362	0.902	0.356	0.244	0.145	0.796
Varicose veins of lower extremity, symptomtic	454.11	Circulatory System	0.036	0.097	0.707	0.977				
Vascular insufficiency of intestine	441	Circulatory System	-0.114	0.161	0.478	0.925				
Ventricular fibrillation and flutter	427.41	Circulatory System	-0.552	0.278	0.047	0.466				
Wegener's granulomatosis	446.4	Circulatory System	0.172	0.203	0.396	0.906				
Anomalies of endocrine glands, congenital	759.1	Congenital Anomalies	0.319	0.244	0.191	0.729				
Anomalies of respiratory system, congenital	748	Congenital Anomalies	0.185	0.384	0.630	0.965				
Cardiac congenital anomalies	747.1	Congenital Anomalies	0.028	0.292	0.925	0.987				
Cardiac shunt/ heart septal defect	747.11	Congenital Anomalies	0.092	0.127	0.468	0.925				
Cleft palate	749.1	Congenital Anomalies	-0.499	0.624	0.424	0.919				
Congenital anomalies of esophagus	750.14	Congenital Anomalies	0.265	0.411	0.519	0.935				
Congenital anomalies of face and neck	749	Congenital Anomalies	-0.095	0.179	0.596	0.964				
Congenital anomalies of female genital organs	751.11	Congenital Anomalies	-0.141	0.242	0.561	0.944				
Congenital anomalies of genital organs	751.1	Congenital Anomalies	-0.499	0.624	0.423	0.919				
Congenital anomalies of great vessels	747.13	Congenital Anomalies	0.543	0.062	1.53E-18	1.88E-16				
Congenital anomalies of intestine	750.21	Congenital Anomalies	0.313	0.268	0.244	0.791				
Congenital anomalies of male genital organs	751.12	Congenital Anomalies	-0.193	0.287	0.502	0.928				
Congenital anomalies of mouth/tongue	750.13	Congenital Anomalies	-0.063	0.177	0.723	0.977				
Congenital anomalies of peripheral vascular system	747.2	Congenital Anomalies	-0.086	0.215	0.689	0.977				
Congenital anomalies of urinary system	751.2	Congenital Anomalies	-0.442	0.485	0.362	0.902				
Congenital anomaly of gallbladder, bile ducts, liver, pancreas	750.22	Congenital Anomalies	0.315	0.369	0.392	0.906				
Congenital cataract and lens anomalies	753.1	Congenital Anomalies	-0.037	0.354	0.916	0.987				
Congenital deformities of feet	755.1	Congenital Anomalies	0.015	0.213	0.944	0.990				
Congenital hip dysplasia and deformity	755.61	Congenital Anomalies	-0.158	0.369	0.669	0.977				
Congenital musculoskeletal deformities of spine	754	Congenital Anomalies	0.246	0.517	0.634	0.965				
Congenital osteodystrophies	756.5	Congenital Anomalies	0.118	0.155	0.446	0.922				
Cystic kidney disease	751.21	Congenital Anomalies	-0.105	0.270	0.697	0.977				
Esophageal atresia/tracheoesophageal fistula	750.11	Congenital Anomalies	-0.513	0.622	0.409	0.913				
Other and unspecified congenital anomalies	759	Congenital Anomalies	0.233	0.364	0.522	0.935				
Other congenital musculoskeletal anomalies	756	Congenital Anomalies	-0.070	0.559	0.901	0.987				
Other specified congenital anomalies of nervous system	752.2	Congenital Anomalies	0.141	0.363	0.698	0.977				
Spina bifida	752.11	Congenital Anomalies	0.133	0.361	0.713	0.977				
Valvular heart disease/ heart chambers	747.12	Congenital Anomalies	-0.295	0.304	0.331	0.876				
Abnormal granulation tissue	701.5	Dermatologic	0.104	0.129	0.418	0.919				
Acne	706.1	Dermatologic	0.058	0.358	0.870	0.987				
Acquired acanthosis nigricans	701.6	Dermatologic	-0.324	0.345	0.348	0.894				
Actinic keratosis	702.1	Dermatologic	0.002	0.049	0.964	0.994				
Alopecia	704.1	Dermatologic	-0.003	0.311	0.992	0.999				
Atopic/contact dermatitis due to other or unspecified	939	Dermatologic	-0.021	0.094	0.823	0.979				
Carbuncle and furuncle	686.1	Dermatologic	0.088	0.051	0.083	0.587	-0.192	0.246	0.437	0.949
Cellulitis and abscess of arm/hand	681.3	Dermatologic	0.012	0.035	0.737	0.977	0.081	0.288	0.778	0.991
Cellulitis and abscess of face/neck	681.2	Dermatologic	-0.024	0.115	0.833	0.979				
Cellulitis and abscess of fingers/toes	681.1	Dermatologic	-0.049	0.117	0.673	0.977				
Cellulitis and abscess of foot, toe	681.6	Dermatologic	0.012	0.035	0.737	0.977	0.081	0.288	0.778	0.991
Cellulitis and abscess of leg, except foot	681.5	Dermatologic	0.012	0.035	0.737	0.977	0.081	0.288	0.778	0.991
Cellulitis and abscess of trunk	681.7	Dermatologic	-0.280	0.160	0.080	0.585				
Changes in skin texture	687.3	Dermatologic	0.003	0.398	0.994	1.000				
Chronic dermatitis due to solar radiation	938.2	Dermatologic	-0.048	0.173	0.781	0.979				
Chronic ulcer of skin	707	Dermatologic	-0.089	0.104	0.392	0.906				
Circumscribed scleroderma	701.3	Dermatologic	0.135	0.430	0.753	0.979				
Congenital anomalies of skin	691	Dermatologic	-0.656	0.435	0.132	0.648				
Corns and callosities	700	Dermatologic	-0.545	0.287	0.058	0.523				
Cutaneous lupus erythematosus	695.41	Dermatologic	-0.318	0.445	0.475	0.925				
Decubitus ulcer	707.1	Dermatologic	-0.523	0.298	0.080	0.585				
Dermatitis due to solar radiation	938	Dermatologic	-0.760	0.375	0.043	0.453				
Dermatomyositis	709.5	Dermatologic	0.209	0.384	0.586	0.958				
Diseases of hair and hair follicles	704	Dermatologic	-0.041	0.037	0.268	0.813	-0.405	0.295	0.170	0.809
Diseases of nail, NOS	703	Dermatologic	-0.282	0.151	0.061	0.538				
Disorder of skin and subcutaneous tissue NOS	689	Dermatologic	0.016	0.033	0.629	0.965	-0.006	0.295	0.984	0.996
Disturbance of skin sensation	687.4	Dermatologic	-0.027	0.071	0.706	0.977	-1.054	0.610	0.084	0.732
Erythema nodosum	695.81	Dermatologic	-0.097	0.422	0.817	0.979				
Hidradenitis	705.3	Dermatologic	0.190	0.204	0.351	0.896				
Hyperhidrosis	705.8	Dermatologic	0.108	0.199	0.588	0.960				
Ingrowing nail	703.1	Dermatologic	0.041	0.091	0.652	0.973				
Keloid scar	701.4	Dermatologic	-0.004	0.185	0.984	0.997	-0.608	0.547	0.266	0.916
Keratoderma, acquired	701.1	Dermatologic	0.135	0.361	0.709	0.977				
Other dyschromia	694.2	Dermatologic	-0.021	0.091	0.817	0.979				
Other hypertrophic and atrophic conditions of skin	701	Dermatologic	-0.057	0.071	0.419	0.919	-1.877	1.024	0.067	0.706
Other local infections of skin and subcutaneous tissue	686	Dermatologic	-0.034	0.092	0.714	0.977				
Other specified diffuse diseases of connective tissue	709.6	Dermatologic	0.115	0.509	0.822	0.979				
Other specified diseases of hair and hair follicles	704.8	Dermatologic	-0.240	0.349	0.490	0.925				
Other specified erythematous conditions	695.8	Dermatologic	-0.044	0.244	0.857	0.987				
Pemphigus and pemphigoid	695.22	Dermatologic	-0.076	0.337	0.822	0.979				
Pilonidal cyst	686.3	Dermatologic	0.043	0.116	0.714	0.977				

Polymyositis	709.4	Dermatologic	-0.051	0.457	0.911	0.987				
Prurigo and Lichen	695.7	Dermatologic	0.071	0.095	0.458	0.925				
Pruritus and related conditions	698	Dermatologic	0.060	0.142	0.672	0.977				
Psoriasis	696.4	Dermatologic	0.119	0.165	0.469	0.925				
Psoriasis vulgaris	696.41	Dermatologic	0.039	0.132	0.770	0.979				
Psoriatic arthropathy	696.42	Dermatologic	0.020	0.148	0.893	0.987				
Pyoderma	686.5	Dermatologic	0.236	0.472	0.618	0.965				
Pyogenic granuloma	686.4	Dermatologic	-0.015	0.132	0.912	0.987				
Rash and other nonspecific skin eruption	687.1	Dermatologic	0.081	0.087	0.352	0.896				
Rosacea	695.3	Dermatologic	0.017	0.222	0.939	0.990				
Sarcoidosis	697	Dermatologic	0.216	0.147	0.141	0.659	0.323	0.426	0.448	0.960
Scar conditions and fibrosis of skin	701.2	Dermatologic	-0.055	0.059	0.355	0.897				
Sebaceous cyst	706.2	Dermatologic	-0.014	0.029	0.616	0.965	-0.248	0.233	0.288	0.929
Seborrheic keratosis	702.2	Dermatologic	-0.002	0.046	0.972	0.994				
Sicca syndrome	709.2	Dermatologic	-0.279	0.283	0.325	0.874				
Superficial cellulitis and abscess	681	Dermatologic	0.019	0.140	0.894	0.987				
Symptoms affecting skin	687	Dermatologic	0.080	0.293	0.784	0.979				
Systemic lupus erythematosus	695.42	Dermatologic	0.161	0.229	0.482	0.925				
Systemic sclerosis	709.3	Dermatologic	0.377	0.245	0.123	0.646				
Toxic erythema	695.1	Dermatologic	0.221	0.265	0.403	0.910				
Unspecified diffuse connective tissue disease	709.7	Dermatologic	0.023	0.049	0.638	0.968	-0.100	0.326	0.760	0.991
Unspecified erythematous condition	695.9	Dermatologic	-0.007	0.199	0.972	0.994				
Urticaria	947	Dermatologic	-0.054	0.126	0.669	0.977	0.366	0.422	0.385	0.949
Vascular disorders of skin	694.3	Dermatologic	-0.352	0.329	0.284	0.833				
Abdominal hernia	550	Digestive	0.086	0.044	0.053	0.506	-0.242	0.299	0.419	0.949
Abnormal results of function study of liver	573.7	Digestive	0.162	0.085	0.057	0.522				
Abnormal serum enzyme levels	573.9	Digestive	-0.438	0.327	0.180	0.720				
Acute appendicitis	540.11	Digestive	-0.016	0.046	0.730	0.977	0.731	0.316	0.021	0.560
Acute gastritis	535.1	Digestive	-0.008	0.089	0.924	0.987	0.239	0.528	0.650	0.991
Acute pancreatitis	577.1	Digestive	-0.033	0.057	0.565	0.944	-0.031	0.511	0.952	0.995
Acute periodontitis	523.31	Digestive	-0.513	0.278	0.065	0.545				
Anal and rectal conditions	565	Digestive	0.091	0.030	0.003	0.065	0.055	0.187	0.768	0.991
Anal and rectal polyp	565.1	Digestive	0.023	0.032	0.476	0.925	-0.027	0.262	0.919	0.995
Anomalies of jaw size/symmetry	526.3	Digestive	-0.543	0.481	0.259	0.810				
Anomalies of tooth position/malocclusion	524.3	Digestive	-0.109	0.176	0.538	0.935				
Appendiceal conditions	540	Digestive	-0.051	0.204	0.801	0.979				
Appendicitis	540.1	Digestive	0.136	0.108	0.207	0.754				
Ascites (non malignant)	572	Digestive	0.065	0.108	0.547	0.935				
Atrophic gastritis	535.2	Digestive	-8.937	0.162	1.000	1.000				
Atrophy of edentulous alveolar ridge	525.2	Digestive	0.351	0.303	0.247	0.798				
Bariatric surgery	539	Digestive	-0.433	0.627	0.489	0.925				
Blood in stool	578.2	Digestive	-0.008	0.060	0.893	0.987	-0.056	0.399	0.889	0.995
Calculus of bile duct	574.2	Digestive	-0.156	0.051	0.002	0.052				
Celiac disease	557.1	Digestive	-0.027	0.080	0.741	0.977				
Cellulitis and abscess of oral soft tissues	528.3	Digestive	-0.067	0.195	0.731	0.977				
Cholangitis	575.1	Digestive	-0.080	0.114	0.481	0.925				
Cholecystitis without cholelithiasis	574.3	Digestive	0.010	0.048	0.828	0.979	-0.246	0.419	0.557	0.967
Cholelithiasis	574.1	Digestive	-0.036	0.031	0.241	0.790	-0.468	0.304	0.124	0.774
Cholelithiasis with acute cholecystitis	574.11	Digestive	-0.152	0.060	0.011	0.170	-2.003	1.021	0.050	0.706
Cholelithiasis with other cholecystitis	574.12	Digestive	-0.045	0.033	0.172	0.713	0.199	0.323	0.538	0.967
Cholesterolosis of gallbladder	575.6	Digestive	-0.206	0.368	0.575	0.953				
Chronic pancreatitis	577.2	Digestive	-0.044	0.143	0.759	0.979				
Chronic periodontitis	523.32	Digestive	0.055	0.082	0.500	0.927	0.202	0.395	0.608	0.980
Cirrhosis of liver without mention of alcohol	571.51	Digestive	0.351	0.135	0.009	0.151				
Cyst and pseudocyst of pancreas	577.3	Digestive	-0.098	0.137	0.472	0.925				
Cyst of the salivary gland	528.41	Digestive	-0.501	0.299	0.094	0.604				
Cysts of oral soft tissues	528.4	Digestive	0.109	0.509	0.831	0.979				
Cysts of the jaws	526.1	Digestive	0.047	0.151	0.757	0.979				
Dental caries	521.1	Digestive	0.045	0.045	0.312	0.863	-0.266	0.161	0.100	0.766
Dentofacial anomalies, including malocclusion	524	Digestive	-0.227	0.492	0.645	0.968				
Diaphragmatic hernia	550.2	Digestive	-0.013	0.024	0.586	0.958	0.271	0.181	0.133	0.787
Diseases and other conditions of the tongue	529	Digestive	0.012	0.078	0.875	0.987				
Diseases of esophagus	530	Digestive	-0.087	0.134	0.519	0.935				
Diseases of lips	528.5	Digestive	0.124	0.090	0.168	0.711				
Diseases of pancreas	577	Digestive	-0.052	0.128	0.688	0.977				
Diseases of pulp and periapical tissues	522	Digestive	0.177	0.110	0.107	0.621				
Diseases of the oral soft tissues, excluding lesions specific fo	528	Digestive	0.042	0.054	0.438	0.922	0.120	0.412	0.770	0.991
Diseases of the salivary glands	527	Digestive	-0.216	0.294	0.464	0.925				
Disorders of esophageal motility	530.5	Digestive	-0.033	0.108	0.762	0.979				
Disorders of tooth development	520	Digestive	0.323	0.443	0.465	0.925				
Disturbance of salivary secretion	527.7	Digestive	-0.429	0.542	0.429	0.919				
Disturbances in tooth eruption	520.2	Digestive	-0.026	0.061	0.674	0.977	0.377	0.362	0.298	0.933
Diverticulosis	562.1	Digestive	-0.007	0.018	0.695	0.977	-0.321	0.161	0.046	0.706
Diverticulum of esophagus, acquired	530.6	Digestive	0.171	0.209	0.413	0.917				
Duodenal ulcer	531.3	Digestive	-0.027	0.069	0.689	0.977	1.365	0.442	0.002	0.128
Duodenitis	535.6	Digestive	0.061	0.051	0.235	0.787	0.351	0.357	0.325	0.946
Dysphagia	532	Digestive	-0.004	0.034	0.898	0.987	0.271	0.212	0.202	0.853
Esophageal bleeding (varices/hemorrhage)	530.2	Digestive	0.048	0.074	0.514	0.935	0.427	0.424	0.313	0.943
Esophagitis, GERD and related diseases	530.1	Digestive	0.048	0.031	0.128	0.646	0.209	0.262	0.424	0.949
Femoral hernia	550.3	Digestive	0.015	0.094	0.873	0.987				
Functional digestive disorders	564	Digestive	-0.081	0.120	0.501	0.927				
GERD	530.11	Digestive	0.021	0.030	0.497	0.927	0.174	0.201	0.386	0.949
Gastric ulcer	531.2	Digestive	0.015	0.050	0.758	0.979	-0.028	0.317	0.930	0.995
Gastritis and duodenitis	535	Digestive	0.064	0.024	0.008	0.141	0.112	0.142	0.431	0.949
Gastroesophageal laceration-hemorrhage syndrome	530.7	Digestive	-0.104	0.172	0.547	0.935				
Gastrointestinal complications	569.2	Digestive	-0.191	0.177	0.281	0.833				
Gastrojejunal ulcer	531.5	Digestive	-1.896	1.020	0.063	0.539				
Gingival and periodontal diseases	523	Digestive	0.032	0.120	0.793	0.979	-0.253	0.561	0.651	0.991
Gingivitis	523.1	Digestive	-0.064	0.310	0.836	0.979				
Glossitis	529.1	Digestive	-0.256	0.168	0.126	0.646				
Heartburn	530.9	Digestive	0.059	0.075	0.429	0.919	-0.541	0.546	0.322	0.946
Hematemesis	578.1	Digestive	-0.076	0.068	0.263	0.813	0.213	0.445	0.632	0.991
Hemorrhage from gastrointestinal ulcer	531.1	Digestive	0.086	0.101	0.396	0.906				
Hemorrhage of gastrointestinal tract	578.9	Digestive	0.018	0.034	0.600	0.964	-0.262	0.219	0.232	0.903
Hemorrhage of rectum and anus	578.8	Digestive	0.002	0.026	0.950	0.990	0.131	0.147	0.374	0.948
Hepatomegaly	573.3	Digestive	-0.340	0.546	0.533	0.935				
Hereditary disturbances in tooth structure	520.1	Digestive	-0.455	0.540	0.399	0.906				
Hypertrophy of salivary gland	527.1	Digestive	-0.463	0.408	0.257	0.808				
Ileostomy status	559	Digestive	0.159	0.080	0.046	0.464				
Impaction of intestine	560.2	Digestive	-0.173	0.196	0.378	0.906				
Inflammatory conditions of jaw	526.5	Digestive	-0.340	0.257	0.187	0.720				
Inguinal hernia	550.1	Digestive	-0.057	0.021	0.006	0.109	-0.121	0.192	0.527	0.967
Intestinal malabsorption (non-celiac)	557	Digestive	-0.107	0.232	0.644	0.968				
Irritable Bowel Syndrome	564.1	Digestive	0.002	0.069	0.974	0.994				
Jaundice (not of newborn)	573.5	Digestive	0.044	0.116	0.703	0.977				
Jaw disease NOS	526.9	Digestive	-0.701	0.238	0.003	0.076				
Leukoplakia of oral mucosa	528.6	Digestive	-0.142	0.143	0.320	0.874				
Liver abscess and sequelae of chronic liver disease	571.8	Digestive	0.196	0.119	0.099	0.607				
Noninfectious gastroenteritis	558	Digestive	0.043	0.025	0.082	0.587	-0.204	0.217	0.347	0.946

Nonspecific abnormal findings in stool contents	579.8	Digestive	-0.028	0.068	0.686	0.977	0.438	0.390	0.262	0.916
Nonspecific abnormal findings on radiological and other exa	575.9	Digestive	-0.142	0.218	0.515	0.935				
Obstruction of bile duct	575.2	Digestive	0.029	0.089	0.742	0.978				
Oral aphthae	528.12	Digestive	0.557	0.458	0.224	0.780				
Other chronic nonalcoholic liver disease	571.5	Digestive	0.032	0.129	0.803	0.979				
Other diseases of the teeth and supporting structures	525	Digestive	-0.064	0.052	0.216	0.766	0.042	0.236	0.859	0.991
Other disorders of biliary tract	575.8	Digestive	0.026	0.101	0.797	0.979				
Other disorders of gallbladder	575.7	Digestive	0.031	0.125	0.803	0.979				
Other disorders of intestine	569	Digestive	-0.043	0.053	0.423	0.919	-0.319	0.352	0.364	0.946
Other disorders of liver	573	Digestive	-0.088	0.119	0.461	0.925	0.789	0.452	0.081	0.732
Other disorders of peritoneum	568	Digestive	-0.005	0.245	0.985	0.997				
Other disorders of stomach and duodenum	537	Digestive	0.033	0.064	0.608	0.965	0.242	0.355	0.497	0.967
Other intestinal obstruction	560.4	Digestive	-0.073	0.047	0.121	0.642	-0.408	0.317	0.199	0.844
Other specified diseases of the salivary glands	527.8	Digestive	-0.191	0.253	0.449	0.923				
Other specified gastritis	535.8	Digestive	0.011	0.032	0.735	0.977	0.037	0.184	0.839	0.991
Other symptoms involving abdomen and pelvis	579	Digestive	0.015	0.092	0.869	0.987				
Paralytic ileus	560.1	Digestive	-0.110	0.223	0.620	0.965				
Peptic ulcer, site unspecified	531.4	Digestive	0.186	0.240	0.438	0.922				
Periapical abscess	522.5	Digestive	-0.069	0.081	0.393	0.906	0.065	0.406	0.873	0.992
Peritoneal adhesions (postoperative) (postinfection)	568.1	Digestive	0.112	0.111	0.311	0.863				
Peritoneal or intestinal adhesions	560.3	Digestive	-0.005	0.077	0.951	0.990	-1.510	0.737	0.040	0.706
Peritonitis and retroperitoneal infections	567	Digestive	-0.107	0.120	0.372	0.906				
Personal history of diseases of digestive system	564.9	Digestive	-0.236	0.212	0.265	0.813				
Portal hypertension	571.81	Digestive	0.209	0.246	0.396	0.906				
Primary biliary cirrhosis	571.6	Digestive	-0.112	0.232	0.628	0.965				
Pulpitis and necrosis of tooth pulp	522.1	Digestive	-0.082	0.353	0.816	0.979				
Reflux esophagitis	530.14	Digestive	0.006	0.027	0.818	0.979	0.529	0.213	0.013	0.384
Regional enteritis	555.1	Digestive	-0.049	0.071	0.485	0.925	-0.812	0.751	0.280	0.922
Sialoadenitis	527.2	Digestive	-0.136	0.135	0.311	0.863				
Sialolithiasis	528.7	Digestive	-0.067	0.154	0.661	0.977				
Splenomegaly	579.2	Digestive	-0.537	0.406	0.186	0.720				
Stomatitis and mucositis (ulcerative)	528.11	Digestive	0.001	0.180	0.997	1.000				
Stricture and stenosis of esophagus	530.3	Digestive	0.034	0.065	0.600	0.964	-0.053	0.574	0.926	0.995
Symptoms involving digestive system	561	Digestive	0.024	0.023	0.296	0.855	0.064	0.179	0.719	0.991
Temporomandibular joint disorder, unspecified	526.41	Digestive	-0.011	0.171	0.947	0.990				
Toxic gastroenteritis and colitis	569.1	Digestive	-0.058	0.158	0.714	0.977				
Ulcer of esophagus	530.12	Digestive	-0.041	0.044	0.349	0.896	0.131	0.572	0.819	0.991
Ulceration of intestine	556.1	Digestive	0.065	0.129	0.613	0.965				
Ulceration of the lower GI tract	556	Digestive	0.047	0.157	0.767	0.979				
Ulcerative colitis	555.2	Digestive	0.096	0.050	0.054	0.508	-0.097	0.459	0.832	0.991
Ulcerative colitis (chronic)	555.21	Digestive	0.146	0.098	0.138	0.653				
Umbilical hernia	550.4	Digestive	0.024	0.044	0.576	0.953	-0.168	0.259	0.517	0.967
Ventral hernia	550.5	Digestive	0.066	0.043	0.130	0.646	-0.370	0.307	0.228	0.895
Acidosis	276.41	Endocrine/Metabolic	0.026	0.341	0.939	0.990				
Acromegaly and gigantism	253.11	Endocrine/Metabolic	-0.925	0.397	0.020	0.273				
Alanine aminotransferace		Endocrine/Metabolic	0.020	0.004	1.45E-06	7.627E-05	-0.009	0.027	0.729	0.991
Albumin		Endocrine/Metabolic	-0.082	0.012	1.68E-12	1.46E-10	-0.060	0.081	0.459	0.964
Alkaline phosphatase		Endocrine/Metabolic	-0.007	0.004	0.099	0.610	-0.034	0.028	0.234	0.904
Amyloidosis	270.33	Endocrine/Metabolic	0.032	0.253	0.901	0.987				
Anorexia	260.6	Endocrine/Metabolic	-0.221	0.267	0.407	0.913				
Apolipoprotein A		Endocrine/Metabolic	-0.002	0.001	0.036	0.408	-0.012	0.007	0.109	0.766
Apolipoprotein B		Endocrine/Metabolic	0.015	0.001	9.54E-48	2.01E-45	0.036	0.007	1.41E-07	1.804E-05
Asparate aminotransferase		Endocrine/Metabolic	0.012	0.004	0.005	0.104	-0.014	0.027	0.620	0.986
Bioavailable Testosterone, Females		Endocrine/Metabolic	0.020	0.007	0.004	0.083	0.040	0.043	0.348	0.946
Bioavailable Testosterone, Males		Endocrine/Metabolic	-0.002	0.006	0.699	0.977	0.014	0.043	0.747	0.991
C-reactive protein		Endocrine/Metabolic	-0.012	0.004	0.008	0.141	-0.041	0.028	0.154	0.796
Calcium		Endocrine/Metabolic	-0.002	0.000	3.00E-07	1.768E-05	0.000	0.003	0.923	0.995
Cholesterol		Endocrine/Metabolic	0.140	0.005	3.57E-172	2.63E-169	0.200	0.036	2.00E-08	3.40E-06
Chondrocalcinosis	274.21	Endocrine/Metabolic	0.140	0.158	0.378	0.906				
Chronic lymphocytic thyroiditis	245.21	Endocrine/Metabolic	-0.121	0.351	0.730	0.977				
Creatinine		Endocrine/Metabolic	0.011	0.004	0.002	0.045	-0.011	0.022	0.622	0.986
Crystal arthropathies	274.2	Endocrine/Metabolic	0.168	0.263	0.523	0.935				
Cushing's syndrome	255.11	Endocrine/Metabolic	-0.071	0.310	0.817	0.979				
Cystatin C		Endocrine/Metabolic	-0.003	0.004	0.436	0.922	0.012	0.027	0.649	0.991
Deficiency of humoral immunity	279.11	Endocrine/Metabolic	-0.109	0.243	0.653	0.973				
Diabetes insipidus	253.3	Endocrine/Metabolic	0.041	0.506	0.936	0.990				
Diabetic retinopathy	250.7	Endocrine/Metabolic	0.164	0.133	0.216	0.766				
Diastolic blood pressure		Endocrine/Metabolic	0.114	0.049	0.021	0.281	0.155	0.350	0.658	0.991
Direct bilirubin		Endocrine/Metabolic	-0.011	0.005	0.015	0.213	-0.009	0.031	0.761	0.991
Disorders involving the immune mechanism	279	Endocrine/Metabolic	-0.551	0.620	0.374	0.906				
Disorders of adrenal glands	255	Endocrine/Metabolic	-0.071	0.279	0.800	0.979				
Disorders of calcium/phosphorus metabolism	275.5	Endocrine/Metabolic	0.160	0.105	0.127	0.646				
Disorders of magnesium metabolism	275.3	Endocrine/Metabolic	-0.089	0.186	0.634	0.965				
Disorders of phosphorus metabolism	275.53	Endocrine/Metabolic	0.128	0.361	0.724	0.977				
Disorders of the pituitary gland and its hypothalamic control	253	Endocrine/Metabolic	0.165	0.287	0.565	0.944				
Electrolyte imbalance	276.1	Endocrine/Metabolic	0.439	0.318	0.167	0.711				
Exophthalmos	242.3	Endocrine/Metabolic	0.117	0.466	0.802	0.979				
Fasting glucose		Endocrine/Metabolic	0.014	0.012	0.258	0.810	0.021	0.045	0.642	0.991
Fluid overload	276.6	Endocrine/Metabolic	0.217	0.178	0.223	0.780				
Free Testosterone, Females		Endocrine/Metabolic	0.024	0.007	0.000	0.015	0.045	0.043	0.292	0.933
Free Testosterone, Males		Endocrine/Metabolic	0.002	0.006	0.752	0.979	0.008	0.043	0.848	0.991
Gamma glutamyltransferase		Endocrine/Metabolic	0.000	0.004	0.923	0.987	-0.047	0.027	0.087	0.735
Glucocorticoid deficiency	255.21	Endocrine/Metabolic	-0.068	0.148	0.645	0.968				
Glycated hemoglobin (HbA1c)		Endocrine/Metabolic	0.087	0.016	6.75E-08	4.14E-06	-0.150	0.166	0.366	0.946
Gout	274.1	Endocrine/Metabolic	-0.081	0.119	0.497	0.927				
Gouty arthropathy	274.11	Endocrine/Metabolic	0.114	0.256	0.656	0.975				
Graves' disease	242.1	Endocrine/Metabolic	0.120	0.176	0.494	0.925				
HDL-cholesterol		Endocrine/Metabolic	-0.005	0.004	0.232	0.787	-0.040	0.028	0.151	0.796
Hyperaldosteronism	255.12	Endocrine/Metabolic	-0.980	0.524	0.061	0.538				
Hypercholesterolemia	272.11	Endocrine/Metabolic	0.065	0.463	0.888	0.987				
Hyperosmolality and/or hypernatremia	276.11	Endocrine/Metabolic	-0.025	0.459	0.957	0.994				
Hyperparathyroidism	252.1	Endocrine/Metabolic	-0.007	0.109	0.948	0.990				
Hyperpotassemia	276.13	Endocrine/Metabolic	0.106	0.108	0.324	0.874				
Hypoglycemia	251.1	Endocrine/Metabolic	0.014	0.099	0.888	0.987	-0.441	0.546	0.419	0.949
Hypopotassemia	276.14	Endocrine/Metabolic	-0.235	0.157	0.134	0.650				
Hyposmolality and/or hyponatremia	276.12	Endocrine/Metabolic	0.139	0.088	0.115	0.626				
Hypothyroidism NOS	244.4	Endocrine/Metabolic	0.100	0.189	0.599	0.964				
Hypovolemia	276.5	Endocrine/Metabolic	-0.053	0.112	0.639	0.968				
Iatrogenic endocrine disorders	258	Endocrine/Metabolic	-0.282	0.366	0.440	0.922				
Immunity deficiency	279.1	Endocrine/Metabolic	-0.219	0.348	0.529	0.935				
Impaired fasting glucose	250.41	Endocrine/Metabolic	0.514	0.310	0.097	0.605				
Insulin-like growth factor 1		Endocrine/Metabolic	-0.014	0.004	0.001	0.037	-0.002	0.028	0.953	0.995
LDL-cholesterol		Endocrine/Metabolic	0.129	0.004	0.000	0.000	0.181	0.028	1.12E-10	5.71E-08
Localized adiposity	278.3	Endocrine/Metabolic	0.281	0.324	0.386	0.906				
Macroglobulinemia	270.35	Endocrine/Metabolic	-0.390	0.327	0.234	0.787				
Mineral deficiency NEC	262	Endocrine/Metabolic	0.269	0.161	0.095	0.604				
Non-HDL cholesterol		Endocrine/Metabolic	0.115	0.004	4.42E-151	2.17E-148	0.181	0.029	4.28E-10	1.09E-07
Nonspecific abnormal results of other endocrine function stu	259.1	Endocrine/Metabolic	0.192	0.407	0.636	0.967				
Nontoxic multinodular goiter	241.2	Endocrine/Metabolic	0.062	0.103	0.547	0.935	0.926	0.353	0.009	0.384

Nontoxic uninodular goiter	241.1	Endocrine/Metabolic	0.100	0.115	0.385	0.906				
Obesity	278.1	Endocrine/Metabolic	0.155	0.101	0.126	0.646	0.788	0.314	0.012	0.384
Oestradiol		Endocrine/Metabolic	0.004	0.009	0.623	0.965	0.076	0.047	0.104	0.766
Oestradiol, Females		Endocrine/Metabolic	0.008	0.012	0.500	0.927	0.115	0.062	0.063	0.706
Oestradiol, Males		Endocrine/Metabolic	-0.004	0.022	0.870	0.987	0.081	0.116	0.484	0.967
Other abnormal glucose	250.42	Endocrine/Metabolic	0.288	0.189	0.128	0.646				
Other disorders of lipoid metabolism	277.5	Endocrine/Metabolic	0.500	0.379	0.187	0.720				
Other disorders of metabolism	277	Endocrine/Metabolic	0.040	0.210	0.851	0.984				
Other disorders of neurohypophysis	253.7	Endocrine/Metabolic	0.165	0.250	0.509	0.934				
Other disorders of thyroid	246	Endocrine/Metabolic	0.499	0.205	0.015	0.213				
Other endocrine disorders	259	Endocrine/Metabolic	-0.228	0.332	0.492	0.925				
Other immunological findings	279.7	Endocrine/Metabolic	0.320	0.190	0.092	0.604				
Ovarian dysfunction	256	Endocrine/Metabolic	-0.180	0.452	0.691	0.977				
Paraproteinemia	270.32	Endocrine/Metabolic	-0.425	0.217	0.050	0.486				
Phosphate		Endocrine/Metabolic	-0.002	0.001	0.001	0.028	0.006	0.005	0.249	0.916
Pituitary hyperfunction	253.1	Endocrine/Metabolic	-0.084	0.500	0.866	0.987				
Pituitary hypofunction	253.2	Endocrine/Metabolic	-0.215	0.217	0.321	0.874				
Polycystic ovaries	256.4	Endocrine/Metabolic	-0.416	0.544	0.444	0.922				
Polydipsia	276.8	Endocrine/Metabolic	-0.111	0.420	0.791	0.979				
Protein-calorie malnutrition	260	Endocrine/Metabolic	-1.292	0.733	0.078	0.582				
Proteinuria	269	Endocrine/Metabolic	-0.089	0.238	0.708	0.977				
Pulse pressure		Endocrine/Metabolic	0.013	0.004	0.002	0.052	0.059	0.026	0.025	0.615
Random glucose		Endocrine/Metabolic	0.000	0.005	0.987	0.997	-0.057	0.034	0.090	0.738
Rheumatoid factor		Endocrine/Metabolic	0.026	0.015	0.082	0.587	0.049	0.107	0.649	0.991
Sex hormone binding globulin		Endocrine/Metabolic	-0.027	0.004	1.36E-10	9.98E-09	-0.048	0.028	0.085	0.732
Sex hormone binding globulin (female)		Endocrine/Metabolic	-0.026	0.006	4.594E-05	0.0018264	-0.072	0.039	0.068	0.706
Sex hormone binding globulin (male)		Endocrine/Metabolic	-0.035	0.006	6.30E-08	4.03E-06	-0.015	0.045	0.735	0.991
Simple and unspecified goiter	240	Endocrine/Metabolic	0.258	0.117	0.027	0.337	-0.504	0.644	0.434	0.949
Systolic blood pressure		Endocrine/Metabolic	0.297	0.085	0.001	0.015	0.779	0.574	0.175	0.824
Testicular hypofunction	257.1	Endocrine/Metabolic	0.455	0.250	0.069	0.545				
Thyroiditis	245	Endocrine/Metabolic	0.365	0.479	0.447	0.922				
Thyrotoxicosis with or without goiter	242	Endocrine/Metabolic	-0.123	0.150	0.413	0.917				
Total bilirubin		Endocrine/Metabolic	-0.007	0.004	0.080	0.585	-0.001	0.028	0.978	0.996
Total protein		Endocrine/Metabolic	0.016	0.018	0.373	0.906	0.136	0.135	0.314	0.943
Toxic multinodular goiter	242.2	Endocrine/Metabolic	-0.353	0.362	0.329	0.874				
Triglycerides		Endocrine/Metabolic	-0.019	0.004	5.25E-06	0.000	-0.012	0.028	0.661	0.991
Triglycerides/HDL-C		Endocrine/Metabolic	-0.012	0.004	0.004	0.091	0.015	0.028	0.593	0.974
Type 1 diabetes	250.1	Endocrine/Metabolic	0.033	0.136	0.811	0.979				
Type 1 diabetes with ketoacidosis	250.11	Endocrine/Metabolic	-0.194	0.182	0.285	0.834				
Type 1 diabetes with ophthalmic manifestations	250.13	Endocrine/Metabolic	0.269	0.179	0.134	0.650				
Type 2 diabetes	250.2	Endocrine/Metabolic	0.234	0.085	0.006	0.113	-0.021	0.249	0.932	0.995
Type 2 diabetes		Endocrine/Metabolic	0.088	0.021	2.964E-05	0.001	0.113	0.095	0.237	0.908
Type 2 diabetes with ketoacidosis	250.21	Endocrine/Metabolic	0.258	0.208	0.216	0.766	0.767	0.400	0.055	0.706
Type 2 diabetes with neurological manifestations	250.24	Endocrine/Metabolic	-0.200	0.333	0.547	0.935				
Type 2 diabetes with ophthalmic manifestations	250.23	Endocrine/Metabolic	0.202	0.126	0.109	0.624	0.538	0.512	0.294	0.933
Type 2 diabetes with renal manifestations	250.22	Endocrine/Metabolic	0.129	0.466	0.783	0.979				
Urate		Endocrine/Metabolic	0.449	0.296	0.129	0.646	1.378	2.000	0.491	0.967
Urea		Endocrine/Metabolic	-0.003	0.004	0.419	0.919	0.016	0.028	0.560	0.967
Vitamin B-complex deficiencies	261.2	Endocrine/Metabolic	-0.125	0.262	0.632	0.965				
Vitamin D		Endocrine/Metabolic	-0.019	0.004	1.91E-05	0.001	-0.037	0.029	0.191	0.830
Vitamin D deficiency	261.4	Endocrine/Metabolic	-0.110	0.394	0.780	0.979				
Abnormal findings on examination of urine	598	Genitourinary	-0.044	0.051	0.396	0.906	0.099	0.290	0.733	0.991
Abnormal mammogram	611.1	Genitourinary	0.174	0.363	0.632	0.965				
Abnormal results of function study of kidney	589	Genitourinary	-0.198	0.332	0.552	0.939				
Abnormal spermatozoa	609.2	Genitourinary	-0.016	0.248	0.949	0.990				
Abscess or ulceration of vulva	614.54	Genitourinary	-0.229	0.179	0.200	0.742				
Absent or infrequent menstruation	626.11	Genitourinary	-0.644	0.404	0.111	0.624				
Acute cystitis	592.11	Genitourinary	0.079	0.211	0.708	0.977				
Acute inflammatory pelvic disease	614.31	Genitourinary	-0.139	0.498	0.780	0.979				
Acute prostatitis	601.11	Genitourinary	-0.364	0.203	0.073	0.552				
Acute renal failure	585.1	Genitourinary	0.068	0.052	0.187	0.720	0.213	0.277	0.442	0.952
Albuminuria		Genitourinary	-0.012	0.020	0.546	0.935	-0.038	0.108	0.723	0.991
Atrophy of female genital tract	624.2	Genitourinary	-0.191	0.348	0.583	0.958				
Balanoposthitis	601.4	Genitourinary	-0.349	0.187	0.063	0.539				
Benign neoplasm of breast	610.4	Genitourinary	0.011	0.072	0.883	0.987	-0.015	0.331	0.964	0.996
Bladder neck obstruction	596.1	Genitourinary	0.018	0.064	0.784	0.979	-0.142	0.514	0.782	0.991
Breast disorder NOS	613.9	Genitourinary	0.261	0.188	0.165	0.711				
Calculus of kidney	594.1	Genitourinary	0.041	0.048	0.389	0.906	-0.441	0.446	0.323	0.946
Calculus of lower urinary tract	594.2	Genitourinary	-0.092	0.095	0.335	0.879				
Calculus of ureter	594.3	Genitourinary	0.028	0.051	0.586	0.958	-0.245	0.455	0.590	0.974
Cervicitis and endocervicitis	614.51	Genitourinary	0.064	0.135	0.634	0.965				
Chronic cystitis	592.12	Genitourinary	0.030	0.100	0.766	0.979				
Chronic glomerulonephritis, NOS	580.14	Genitourinary	-0.554	0.538	0.303	0.862				
Chronic inflammatory pelvic disease	614.32	Genitourinary	0.079	0.201	0.694	0.977				
Chronic interstitial cystitis	592.13	Genitourinary	0.011	0.164	0.947	0.990				
Chronic kidney disease		Genitourinary	0.006	0.034	0.863	0.987	-0.440	0.251	0.080	0.732
Chronic prostatitis	601.12	Genitourinary	0.014	0.114	0.900	0.987				
Chronic renal failure [CKD]	585.3	Genitourinary	0.059	0.083	0.474	0.925	-1.301	0.522	0.013	0.384
Congenital anomalies of breast	612.3	Genitourinary	0.195	0.307	0.526	0.935				
Cyst of kidney, acquired	586.2	Genitourinary	-0.024	0.190	0.899	0.987				
Cyst or abscess of Bartholin's gland	614.53	Genitourinary	-0.102	0.105	0.329	0.874	-0.649	0.623	0.298	0.933
Cystic mastopathy	610.1	Genitourinary	-0.060	0.111	0.586	0.958				
Cystitis	592.1	Genitourinary	0.025	0.067	0.705	0.977	-1.165	0.747	0.119	0.774
Disorders of menstruation and other abnormal bleeding from	626	Genitourinary	-0.029	0.078	0.710	0.977	-0.486	0.452	0.283	0.922
Disorders of penis	604	Genitourinary	0.176	0.096	0.065	0.545				
Disorders of uterus, NEC	619.2	Genitourinary	0.024	0.087	0.779	0.979	-0.687	0.623	0.270	0.916
Disorders resulting from impaired renal function	588	Genitourinary	0.228	0.437	0.602	0.965				
Dysmenorrhea	626.2	Genitourinary	-0.262	0.150	0.080	0.585				
Dyspareunia	625.1	Genitourinary	-0.129	0.127	0.311	0.863				
Dysplasia of female genital organs	620	Genitourinary	-0.231	0.348	0.506	0.932				
Dystrophy of female genital tract	624.1	Genitourinary	-0.024	0.169	0.889	0.987				
Dysuria	599.3	Genitourinary	0.087	0.119	0.465	0.925				
End stage renal disease		Genitourinary	0.096	0.283	0.734	0.977				
Endometrial hyperplasia	621	Genitourinary	-0.055	0.100	0.578	0.955				
Endometriosis	615	Genitourinary	0.069	0.062	0.269	0.813	-0.222	0.432	0.607	0.980
Erectile dysfunction [ED]	605	Genitourinary	-0.025	0.140	0.859	0.987	0.037	0.543	0.946	0.995
Estimated glomerular filtration rate (creatinine and cystatin C)		Genitourinary	-0.002	0.004	0.625	0.965	-0.005	0.025	0.843	0.991
Excessive or frequent menstruation	626.12	Genitourinary	0.036	0.034	0.295	0.854	0.026	0.166	0.878	0.993
Fibroadenosis of breast	610.2	Genitourinary	0.131	0.208	0.528	0.935				
Fibrosclerosis of breast	610.3	Genitourinary	0.212	0.187	0.256	0.808				
Frequency of urination and polyuria	599.5	Genitourinary	0.067	0.056	0.232	0.787	0.079	0.351	0.823	0.991
Functional disorders of bladder	596.5	Genitourinary	0.008	0.075	0.917	0.987				
Genital prolapse	618	Genitourinary	-0.223	0.246	0.365	0.904				
Hematuria	593	Genitourinary	-0.049	0.023	0.031	0.367	-0.042	0.150	0.776	0.991
Hydrocele	603.1	Genitourinary	-0.050	0.075	0.508	0.933	-0.124	0.429	0.772	0.991
Hydronephrosis	595	Genitourinary	-0.044	0.060	0.469	0.925	-0.983	0.610	0.107	0.766
Hyperplasia of prostate	600	Genitourinary	-0.019	0.031	0.534	0.935	0.331	0.209	0.113	0.768
Hypertrophy of breast (Gynecomastia)	612.2	Genitourinary	-0.146	0.107	0.172	0.713				
Hypertrophy of female genital organs	623	Genitourinary	-0.134	0.154	0.384	0.906				

Infertility, female	626.8	Genitourinary	0.035	0.097	0.720	0.977	-0.624	0.501	0.213	0.869
Infertility, male	609.1	Genitourinary	0.104	0.406	0.799	0.979				
Inflammatory disease of breast	613.1	Genitourinary	-0.026	0.111	0.814	0.979				
Inflammatory disease of cervix, vagina, and vulva	614.5	Genitourinary	-0.461	0.288	0.110	0.624				
Inflammatory diseases of uterus, except cervix	614.4	Genitourinary	0.182	0.210	0.385	0.906				
Irregular menstrual bleeding	626.14	Genitourinary	-0.072	0.054	0.182	0.720	-0.900	0.477	0.060	0.706
Irregular menstrual cycle	626.13	Genitourinary	0.002	0.078	0.981	0.997	0.056	0.526	0.915	0.995
Irregular menstrual cycle/bleeding	626.1	Genitourinary	-0.039	0.074	0.600	0.964				
Lump or mass in breast	611.3	Genitourinary	0.003	0.069	0.960	0.994	-0.390	0.422	0.356	0.946
Mastodynia	613.5	Genitourinary	-0.432	0.442	0.328	0.874				
Menopausal and postmenopausal disorders	627	Genitourinary	0.113	0.146	0.439	0.922				
Mucous polyp of cervix	622.2	Genitourinary	-0.109	0.057	0.057	0.523	0.258	0.395	0.513	0.967
Nephritis and nephropathy with pathological lesion	580.32	Genitourinary	-0.091	0.238	0.703	0.977				
Nephrotic syndrome without mention of glomerulonephritis	580.2	Genitourinary	-0.182	0.241	0.450	0.924				
Non-proliferative glomerulonephritis	580.12	Genitourinary	0.063	0.242	0.796	0.979				
Noninflammatory disorders of cervix	619.3	Genitourinary	-0.118	0.092	0.198	0.736	-0.862	0.619	0.164	0.809
Noninflammatory disorders of ovary, fallopian tube, and broa	619.1	Genitourinary	-0.005	0.149	0.975	0.994				
Noninflammatory disorders of vagina	619.4	Genitourinary	-0.003	0.084	0.969	0.994				
Noninflammatory disorders of vulva and perineum	619.5	Genitourinary	-0.048	0.079	0.543	0.935	0.818	0.463	0.078	0.732
Noninflammatory female genital disorders	619	Genitourinary	0.246	0.215	0.253	0.807				
Other abnormality of urination	599.9	Genitourinary	0.170	0.080	0.034	0.393				
Other disorders of bladder	596	Genitourinary	0.008	0.038	0.834	0.979	-0.406	0.275	0.140	0.796
Other disorders of male genital organs	608	Genitourinary	0.072	0.054	0.184	0.720	-1.195	0.610	0.050	0.706
Other disorders of prostate	602	Genitourinary	0.059	0.087	0.495	0.925				
Other disorders of testis	603	Genitourinary	-0.025	0.085	0.769	0.979	0.347	0.488	0.477	0.964
Other disorders of the kidney and ureters	586	Genitourinary	-0.173	0.115	0.133	0.650				
Other disorders of urethra and urinary tract	597	Genitourinary	0.011	0.113	0.920	0.987				
Other inflammatory disorders of male genital organs	601.8	Genitourinary	-0.056	0.160	0.727	0.977				
Other signs and symptoms in breast	613.7	Genitourinary	0.030	0.123	0.810	0.979				
Other specified benign mammary dysplasias	610.8	Genitourinary	-0.069	0.112	0.537	0.935				
Other specified disorders of breast	613.8	Genitourinary	-0.140	0.169	0.407	0.913				
Other symptoms/disorders or the urinary system	599	Genitourinary	-0.012	0.042	0.777	0.979	0.086	0.258	0.738	0.991
Ovarian cyst	628	Genitourinary	0.002	0.052	0.964	0.994	-0.081	0.334	0.808	0.991
Pain and other symptoms associated with female genital orga	625	Genitourinary	-0.018	0.084	0.833	0.979	-0.174	0.507	0.731	0.991
Pelvic inflammatory disease (PID)	614.3	Genitourinary	0.000	0.265	0.999	1.000				
Pelvic inflammatory disease, NOS	614.33	Genitourinary	0.029	0.174	0.868	0.987				
Pelvic peritoneal adhesions, female (postoperative) (postinfec	614.1	Genitourinary	0.049	0.116	0.671	0.977	-0.233	0.462	0.614	0.982
Polyp of corpus uteri	622.1	Genitourinary	-0.045	0.034	0.184	0.720	0.000	0.201	0.999	0.999
Polyp of female genital organs	622	Genitourinary	0.014	0.209	0.948	0.990				
Postmenopausal atrophic vaginitis	627.3	Genitourinary	0.018	0.150	0.906	0.987				
Postmenopausal bleeding	627.1	Genitourinary	0.067	0.031	0.031	0.368	-0.197	0.232	0.394	0.949
Premenopausal menorrhagia	627.4	Genitourinary	0.045	0.181	0.805	0.979				
Premenstrual tension syndromes	626.4	Genitourinary	-0.541	0.439	0.218	0.768				
Prolapse of vaginal vault after hysterectomy	618.5	Genitourinary	-0.201	0.134	0.132	0.648				
Prolapse of vaginal walls	618.1	Genitourinary	0.008	0.035	0.824	0.979	-0.520	0.413	0.209	0.864
Proliferative glomerulonephritis	580.11	Genitourinary	-1.229	0.734	0.094	0.604				
Prostatitis	601.1	Genitourinary	-0.011	0.120	0.925	0.987				
Pyelonephritis	590	Genitourinary	-0.008	0.065	0.904	0.987	0.033	0.365	0.927	0.995
Redundant prepuce and phimosis/BXO	604.1	Genitourinary	-0.011	0.051	0.821	0.979	0.847	0.406	0.037	0.706
Renal colic	594.8	Genitourinary	-0.022	0.065	0.730	0.977	-0.503	0.490	0.304	0.941
Renal failure NOS	585.2	Genitourinary	-0.019	0.180	0.917	0.987				
Retention of urine	599.2	Genitourinary	0.022	0.041	0.604	0.965	-0.468	0.349	0.180	0.825
Small kidney	586.11	Genitourinary	0.081	0.428	0.851	0.984				
Stricture/obstruction of ureter	586.4	Genitourinary	-0.076	0.096	0.423	0.919				
Symptomatic menopause	627.2	Genitourinary	0.326	0.238	0.171	0.713				
Urethral discharge	599.7	Genitourinary	-0.439	0.627	0.483	0.925				
Urethral stricture (not specified as infectious)	597.1	Genitourinary	0.026	0.048	0.591	0.961	-0.132	0.339	0.697	0.991
Urethral syndrome	592.21	Genitourinary	-1.471	0.728	0.043	0.453				
Urethritis and urethral syndrome	592.2	Genitourinary	0.313	0.276	0.257	0.808				
Urinary albumin-to-creatine ratio		Genitourinary	-0.008	0.004	0.061	0.538	-0.044	0.028	0.119	0.774
Urinary calculus	594	Genitourinary	0.042	0.111	0.705	0.977				
Urinary complications NEC	597.2	Genitourinary	-0.521	0.311	0.094	0.604				
Urinary incontinence	599.4	Genitourinary	0.016	0.034	0.644	0.968	-0.191	0.327	0.559	0.967
Urinary obstruction	599.1	Genitourinary	0.205	0.231	0.375	0.906				
Urinary potassium-to-creatinine ratio		Genitourinary	-0.008	0.004	0.062	0.539	-0.029	0.027	0.282	0.922
Urinary sodium to creatinine ratio		Genitourinary	0.007	0.004	0.128	0.646	-0.010	0.029	0.728	0.991
Urinary sodium-to-potassium ratio		Genitourinary	0.012	0.004	0.005	0.097	0.015	0.029	0.609	0.980
Urinary tract infection	591	Genitourinary	-0.035	0.027	0.195	0.736	-0.043	0.194	0.826	0.991
Urine albumin		Genitourinary	-0.003	0.004	0.456	0.925	-0.021	0.026	0.421	0.949
Urine creatinine		Genitourinary	0.003	0.004	0.485	0.925	0.011	0.028	0.687	0.991
Urine potassium		Genitourinary	-0.001	0.004	0.845	0.983	-0.012	0.029	0.687	0.991
Urine sodium		Genitourinary	0.011	0.004	0.009	0.150	0.000	0.028	0.992	0.996
Uterine/Uterovaginal prolapse	618.2	Genitourinary	-0.011	0.039	0.781	0.979	-0.613	0.544	0.260	0.916
Vaginal enterocele, congenital or acquired	618.6	Genitourinary	-0.115	0.157	0.463	0.925				
Vaginitis and vulvovaginitis	614.52	Genitourinary	0.147	0.151	0.333	0.876				
Vascular disorders of kidney/hypertrophy	586.3	Genitourinary	-0.099	0.498	0.842	0.983				
stress incontinence, female	624.9	Genitourinary	-0.017	0.040	0.672	0.977	-0.106	0.467	0.821	0.991
Abnormality of red blood cells	289.9	Hematopoietic	-0.382	0.444	0.390	0.906				
Allergic purpura	287.2	Hematopoietic	0.112	0.432	0.795	0.979				
Anemia in neoplastic disease	285.22	Hematopoietic	0.176	0.167	0.294	0.853				
Anemia of chronic disease	285.2	Hematopoietic	0.078	0.154	0.613	0.965				
Aplastic anemia	284	Hematopoietic	0.200	0.143	0.161	0.703				
Autoimmune hemolytic anemias	283.1	Hematopoietic	0.130	0.261	0.619	0.965				
Basophil %		Hematopoietic	0.002	0.004	0.686	0.977	0.068	0.030	0.023	0.579
Basophil count		Hematopoietic	0.005	0.004	0.183	0.720	0.039	0.027	0.144	0.796
Congenital factor VIII disorder	286.13	Hematopoietic	-0.680	0.616	0.269	0.813				
Diseases of spleen	289.5	Hematopoietic	0.012	0.325	0.970	0.994				
Diseases of white blood cells	288	Hematopoietic	-0.091	0.197	0.646	0.968				
Disorders of iron metabolism	275.1	Hematopoietic	-0.023	0.097	0.809	0.979				
Eosinophil %		Hematopoietic	-0.003	0.004	0.545	0.935	-0.004	0.030	0.890	0.995
Eosinophil count		Hematopoietic	-0.003	0.004	0.569	0.945	-0.012	0.030	0.677	0.991
Eosinophilia	288.3	Hematopoietic	0.322	0.389	0.408	0.913				
Folate-deficiency anemia	281.13	Hematopoietic	0.237	0.236	0.315	0.870				
Hematocrit %		Hematopoietic	-0.037	0.012	0.003	0.064	-0.052	0.091	0.573	0.968
Hemoglobin concentration		Hematopoietic	-0.016	0.004	0.000	0.008	-0.009	0.032	0.774	0.991
Hemorrhagic disorder due to intrinsic circulating anticoagula	286.5	Hematopoietic	-0.245	0.492	0.619	0.965				
High light scatter reticulocyte %		Hematopoietic	0.010	0.004	0.024	0.301	0.042	0.030	0.161	0.809
High light scatter reticulocyte count		Hematopoietic	0.009	0.004	0.052	0.495	0.043	0.030	0.149	0.796
Immature reticulocyte fraction		Hematopoietic	0.000	0.000	0.472	0.925	0.006	0.002	0.002	0.122
Iron deficiency anemia secondary to blood loss (chronic)	280.2	Hematopoietic	0.213	0.198	0.282	0.833				
Iron deficiency anemias, unspecified or not due to blood loss	280.1	Hematopoietic	0.067	0.030	0.025	0.322	-0.357	0.214	0.095	0.754
Lymphadenitis	289.4	Hematopoietic	0.006	0.065	0.926	0.987	-0.341	0.370	0.356	0.946
Lymphoctye %		Hematopoietic	0.004	0.031	0.900	0.987	-0.195	0.265	0.461	0.964
Lymphoctye count		Hematopoietic	0.003	0.004	0.560	0.944	-0.027	0.030	0.360	0.946
Mean corpuscular hemoglobin		Hematopoietic	-0.007	0.004	0.106	0.621	-0.019	0.029	0.525	0.967
Mean corpuscular hemoglobin concentration		Hematopoietic	-0.007	0.004	0.116	0.626	0.023	0.029	0.435	0.949
Mean corpuscular volume		Hematopoietic	-0.004	0.004	0.326	0.874	-0.034	0.029	0.248	0.916
Mean platelet volume		Hematopoietic	0.010	0.004	0.026	0.324	0.013	0.030	0.671	0.991
Mean reticulocyte volume		Hematopoietic	-0.120	0.033	0.000	0.009	0.014	0.238	0.952	0.995

Mean sphered cell volume		Hematopoietic	-0.041	0.022	0.068	0.545	0.005	0.181	0.976	0.996
Monocyte %		Hematopoietic	0.006	0.004	0.188	0.721	0.061	0.029	0.037	0.706
Monocyte count		Hematopoietic	0.005	0.004	0.272	0.816	0.041	0.030	0.169	0.809
Neutropenia	288.11	Hematopoietic	-0.055	0.071	0.439	0.922	0.028	0.471	0.952	0.995
Neutrophil %		Hematopoietic	-0.017	0.036	0.630	0.965	0.051	0.290	0.861	0.991
Neutrophil count		Hematopoietic	0.001	0.004	0.894	0.987	-0.005	0.029	0.865	0.991
Nucleated red blood cell %		Hematopoietic	0.000	0.001	0.715	0.977	0.012	0.017	0.495	0.967
Nucleated red blood cell count		Hematopoietic	0.000	0.001	0.749	0.979	0.014	0.017	0.404	0.949
Other and unspecified coagulation defects	286.7	Hematopoietic	-0.190	0.390	0.626	0.965				
Other anemias	285	Hematopoietic	0.065	0.031	0.037	0.416	-0.129	0.176	0.465	0.964
Other diseases of blood and blood-forming organs	289	Hematopoietic	-0.048	0.139	0.729	0.977				
Other hereditary hemolytic anemias	282.9	Hematopoietic	0.111	0.464	0.811	0.979				
Other vitamin B12 deficiency anemia	281.12	Hematopoietic	-0.100	0.455	0.825	0.979				
Pernicious anemia	281.11	Hematopoietic	0.014	0.357	0.969	0.994				
Platelet count		Hematopoietic	-0.022	0.004	3.45E-07	1.953E-05	-0.071	0.028	0.012	0.384
Platelet crit		Hematopoietic	-0.019	0.004	4.64E-06	0.0002	-0.077	0.027	0.005	0.283
Platelet distribution width		Hematopoietic	-0.010	0.004	0.017	0.236	-0.001	0.029	0.959	0.996
Polycythemia, secondary	289.8	Hematopoietic	-0.267	0.174	0.125	0.646				
Primary thrombocytopenia	287.31	Hematopoietic	-0.084	0.155	0.589	0.960				
Purpura and other hemorrhagic conditions	287	Hematopoietic	-1.118	0.737	0.130	0.646				
Red blood cell count		Hematopoietic	-0.007	0.004	0.064	0.545	0.000	0.026	0.985	0.996
Red blood cell distribution width		Hematopoietic	0.005	0.004	0.228	0.785	0.011	0.029	0.699	0.991
Reticulocyte %		Hematopoietic	0.012	0.004	0.006	0.113	-0.002	0.030	0.952	0.995
Reticulocyte count		Hematopoietic	0.010	0.004	0.021	0.281	-0.001	0.030	0.978	0.996
Secondary thrombocytopenia	287.32	Hematopoietic	-0.462	0.540	0.392	0.906				
Sickle cell anemia	282.5	Hematopoietic					0.034	0.476	0.943	0.995
Spontaneous ecchymoses	287.1	Hematopoietic	-0.201	0.368	0.585	0.958				
Thrombocytopenia	287.3	Hematopoietic	0.146	0.121	0.229	0.785				
Von willebrand's disease	286.11	Hematopoietic	-0.574	0.536	0.284	0.833				
White blood cell count		Hematopoietic	0.002	0.004	0.705	0.977	-0.009	0.029	0.750	0.991
Arthropod-borne diseases	133	Infectious Diseases	-0.001	0.313	0.999	1.000	-0.023	0.226	0.920	0.995
Aspergillosis	117.4	Infectious Diseases	-0.483	0.299	0.107	0.621				
Bacterial enteritis	8.5	Infectious Diseases	-0.028	0.077	0.720	0.977	0.298	0.317	0.346	0.946
Bacterial infection NOS	41	Infectious Diseases	0.115	0.165	0.484	0.925				
Candidiasis	112	Infectious Diseases	0.020	0.108	0.850	0.984				
Chronic hepatitis	70.4	Infectious Diseases	-0.135	0.193	0.483	0.925				
Dermatophytosis of nail	110.11	Infectious Diseases	0.062	0.428	0.886	0.987				
Gram negative septicemia	38.1	Infectious Diseases	-0.025	0.082	0.759	0.979	-0.376	0.635	0.554	0.967
Gram positive septicemia	38.2	Infectious Diseases	-0.133	0.146	0.361	0.902				
HIV infection, symptomatic	71.1	Infectious Diseases	-0.837	0.435	0.054	0.508				
Hepatitis NOS	70.9	Infectious Diseases	0.022	0.143	0.880	0.987				
Herpes simplex	54	Infectious Diseases	0.144	0.363	0.691	0.977				
Herpes zoster	53	Infectious Diseases	0.123	0.174	0.480	0.925				
Herpes zoster with nervous system complications	53.1	Infectious Diseases	0.151	0.270	0.576	0.954				
Infection/inflammation of internal prosthetic device; implant;	81	Infectious Diseases	-0.019	0.074	0.794	0.979	0.282	0.449	0.530	0.967
Infectious mononucleosis	79.2	Infectious Diseases	0.659	0.342	0.054	0.508				
Intestinal helminthiases	134.1	Infectious Diseases	0.154	0.363	0.671	0.977				
Intestinal infection	8	Infectious Diseases	0.005	0.032	0.884	0.987	-0.216	0.257	0.401	0.949
Intestinal infection due to C. difficile	8.52	Infectious Diseases	0.070	0.133	0.597	0.964				
Intestinal infection due to protozoa	8.7	Infectious Diseases	-0.242	0.450	0.591	0.961				
Lyme disease	130.1	Infectious Diseases	-1.052	0.739	0.154	0.694				
Mycoses	117	Infectious Diseases	-0.570	0.620	0.357	0.900				
Other infectious and parasitic diseases	136	Infectious Diseases	0.378	0.187	0.043	0.453				
Postoperative infection	80	Infectious Diseases	-0.011	0.044	0.797	0.979	-0.182	0.300	0.544	0.967
Septicemia	38	Infectious Diseases	0.008	0.039	0.830	0.979	-0.315	0.307	0.306	0.941
Spirochetal infection	130	Infectious Diseases	-1.336	0.732	0.068	0.545				
Staphylococcus infections	41.1	Infectious Diseases	-0.170	0.277	0.539	0.935				
Streptococcus infection	41.2	Infectious Diseases	0.393	0.216	0.068	0.545				
Tuberculosis	10	Infectious Diseases	0.095	0.243	0.695	0.977	-0.032	0.514	0.951	0.995
Varicella infection	79.1	Infectious Diseases	0.086	0.359	0.810	0.979				
Viral Enteritis	8.6	Infectious Diseases	0.024	0.080	0.765	0.979	0.436	0.452	0.335	0.946
Viral hepatitis	70	Infectious Diseases	-0.482	0.408	0.237	0.787				
Viral hepatitis A	70.1	Infectious Diseases	0.342	0.479	0.475	0.925				
Viral hepatitis B	70.2	Infectious Diseases	0.222	0.437	0.612	0.965				
Viral hepatitis C	70.3	Infectious Diseases	-0.167	0.210	0.425	0.919				
Viral infection	79	Infectious Diseases	-0.053	0.060	0.382	0.906	-0.278	0.419	0.508	0.967
Viral warts & HPV	78	Infectious Diseases	-0.045	0.090	0.619	0.965	-16.567	1070.407	0.988	0.996
Adverse drug events and drug allergies	979	Injuries & Poisonings	-0.053	0.159	0.739	0.977				
Adverse effects of sedatives or other central nervous system d	967	Injuries & Poisonings	-0.124	0.322	0.701	0.977				
Allergic reaction to food	930	Injuries & Poisonings	0.115	0.198	0.562	0.944				
Allergies, other	949	Injuries & Poisonings	-0.080	0.135	0.556	0.943				
Anaphylactic shock NOS	946	Injuries & Poisonings	-0.062	0.115	0.590	0.961	0.335	0.395	0.396	0.949
Anticoagulants causing adverse effects	964.1	Injuries & Poisonings	1.021	0.353	0.004	0.086				
Antineoplastic and immunosuppressive drugs causing advers	963.1	Injuries & Poisonings	-0.632	0.618	0.306	0.862				
Certain early complications of trauma or procedure	958	Injuries & Poisonings	0.062	0.156	0.690	0.977				
Complication due to other implant and internal device	859	Injuries & Poisonings	-0.019	0.041	0.646	0.968	0.162	0.283	0.567	0.968
Complication of amputation stump	874	Injuries & Poisonings	0.167	0.191	0.384	0.906				
Complication of colostomy or enterostomy	853	Injuries & Poisonings	-0.159	0.123	0.197	0.736				
Complication of internal orthopedic device	858	Injuries & Poisonings	0.052	0.038	0.178	0.719	0.232	0.352	0.510	0.967
Complications of cardiac/vascular device, implant, and graft	854	Injuries & Poisonings	0.216	0.056	0.000	0.004	-0.506	0.412	0.220	0.875
Complications of transplants and reattached limbs	851	Injuries & Poisonings	-0.319	0.251	0.203	0.750				
Concussion	817	Injuries & Poisonings	-0.082	0.189	0.665	0.977				
Contusion	916	Injuries & Poisonings	0.263	0.085	0.002	0.051				
Dislocation	830	Injuries & Poisonings	0.021	0.067	0.754	0.979	-1.880	1.026	0.067	0.706
Effects radiation NOS	990	Injuries & Poisonings	0.024	0.217	0.913	0.987				
Fracture of ankle and foot	801	Injuries & Poisonings	-0.038	0.059	0.514	0.935				
Fracture of clavicle or scapula	803.3	Injuries & Poisonings	0.066	0.069	0.337	0.881				
Fracture of foot	801.1	Injuries & Poisonings	0.000	0.301	0.999	1.000				
Fracture of hand or wrist	804	Injuries & Poisonings	0.009	0.044	0.834	0.979	0.610	0.474	0.198	0.844
Fracture of humerus	803.1	Injuries & Poisonings	-0.014	0.057	0.801	0.979				
Fracture of lower limb	800	Injuries & Poisonings	0.024	0.046	0.608	0.965	-0.636	0.625	0.309	0.942
Fracture of neck of femur	800.1	Injuries & Poisonings	0.024	0.046	0.600	0.964				
Fracture of patella	800.4	Injuries & Poisonings	-0.013	0.096	0.895	0.987				
Fracture of pelvis	802	Injuries & Poisonings	-0.084	0.084	0.320	0.874				
Fracture of radius and ulna	803.2	Injuries & Poisonings	-0.034	0.033	0.311	0.863	0.541	0.413	0.191	0.830
Fracture of ribs	807	Injuries & Poisonings	-0.008	0.077	0.921	0.987				
Fracture of tibia and fibula	800.3	Injuries & Poisonings	0.095	0.054	0.079	0.585	0.093	0.517	0.857	0.991
Fracture of unspecified bones	809	Injuries & Poisonings	0.131	0.167	0.431	0.920				
Fracture of unspecified part of femur	800.2	Injuries & Poisonings	0.054	0.071	0.443	0.922				
Fracture of upper limb	803	Injuries & Poisonings	0.015	0.145	0.917	0.987				
Fracture of vertebral column without mention of spinal cord i	805	Injuries & Poisonings	-0.133	0.064	0.038	0.427				
Hemorrhage or hematoma complicating a procedure	850	Injuries & Poisonings	0.060	0.048	0.214	0.766	0.252	0.306	0.411	0.949
Injuries to the nervous system	907	Injuries & Poisonings	-0.002	0.099	0.981	0.997				
Internal derangement of knee	835	Injuries & Poisonings	0.013	0.022	0.540	0.935	0.269	0.139	0.054	0.706
Intracranial hemorrhage (injury)	818	Injuries & Poisonings	0.154	0.079	0.050	0.486				
Joint/ligament sprain	840.3	Injuries & Poisonings	-0.013	0.209	0.950	0.990				
Mechanical complication of unspecified genitourinary device	857	Injuries & Poisonings	0.096	0.067	0.152	0.694	-0.235	0.421	0.576	0.968
Open wound of ear	870.2	Injuries & Poisonings	-0.114	0.232	0.623	0.965				
Open wound of foot except toe(s) alone	871.3	Injuries & Poisonings	-0.278	0.245	0.257	0.808				

Open wound of genital organs	870.8	Injuries & Poisonings	0.418	0.358	0.243	0.791				
Open wound of lip and mouth	870.5	Injuries & Poisonings	0.141	0.150	0.348	0.894				
Open wound of neck	870.6	Injuries & Poisonings	-0.009	0.185	0.960	0.994				
Open wound of nose and sinus	870.4	Injuries & Poisonings	0.224	0.188	0.233	0.787				
Open wound of toe(s)	871.4	Injuries & Poisonings	0.862	0.279	0.002	0.052				
Open wound or laceration of eye or eyelid	870.1	Injuries & Poisonings	0.217	0.120	0.072	0.550				
Open wounds of extremities	871	Injuries & Poisonings	0.009	0.045	0.836	0.979	0.771	0.307	0.012	0.384
Open wounds of head; neck; and trunk	870	Injuries & Poisonings	-0.409	0.290	0.159	0.703				
Other open wound of head and face	870.3	Injuries & Poisonings	0.072	0.051	0.158	0.701	-0.318	0.550	0.563	0.967
Poisoning by agents primarily affecting the cardiovascular sy	972	Injuries & Poisonings	-0.416	0.263	0.114	0.626				
Poisoning by agents primarily affecting the gastrointestinal sy	973	Injuries & Poisonings	0.452	0.488	0.354	0.897				
Poisoning by analgesics, antipyretics, and antirheumatics	965	Injuries & Poisonings	0.091	0.067	0.175	0.718				
Poisoning by anticonvulsants and anti-Parkinsonism drugs	966	Injuries & Poisonings	0.002	0.143	0.987	0.997				
Poisoning by drugs primarily affecting the autonomic nervous	971	Injuries & Poisonings	0.052	0.253	0.837	0.979				
Poisoning by hormones and synthetic substitutes	962	Injuries & Poisonings	0.029	0.246	0.906	0.987				
Poisoning by primarily systemic agents	963	Injuries & Poisonings	-0.101	0.421	0.810	0.979				
Poisoning by psychotropic agents	969	Injuries & Poisonings	0.029	0.067	0.667	0.977	-0.129	0.570	0.822	0.991
Salicylates causing adverse effects in therapeutic use	965.3	Injuries & Poisonings	-0.165	0.261	0.527	0.935				
Sepsis	994.2	Injuries & Poisonings	0.005	0.040	0.907	0.987	-0.442	0.331	0.182	0.825
Skull and face fracture and other intercranial injury	819	Injuries & Poisonings	-0.048	0.054	0.380	0.906	0.166	0.471	0.724	0.991
Spinal cord injury without evidence of spinal bone injury	952	Injuries & Poisonings	-0.096	0.288	0.738	0.977				
Sprains and strains	840	Injuries & Poisonings	0.022	0.145	0.881	0.987				
Toxic effect of (non-ethyl) alcohol and petroleum and other so	981	Injuries & Poisonings	0.135	0.432	0.755	0.979				
Toxic effect of carbon monoxide	986	Injuries & Poisonings	0.320	0.325	0.326	0.874				
Toxic effect of noxious substances eaten as food	988	Injuries & Poisonings	0.488	0.454	0.283	0.833				
Toxic effect of other gases, fumes, or vapors	987	Injuries & Poisonings	0.070	0.248	0.778	0.979				
Toxic effect of other metals	985	Injuries & Poisonings	0.764	0.379	0.044	0.455				
Toxic effect of other substances, chiefly nonmedicinal as to so	989	Injuries & Poisonings	0.089	0.379	0.815	0.979				
Toxic effect of venom	913	Injuries & Poisonings	0.305	0.213	0.153	0.694				
Traumatic amputation	872	Injuries & Poisonings	0.066	0.115	0.566	0.944				
Traumatic arthropathy	836	Injuries & Poisonings	0.090	0.103	0.383	0.906				
Adrenergics, inhalant medications		Medications	-0.006	0.018	0.749	0.979	0.164	0.125	0.191	0.830
Agents acting on the renin-angiotensin system		Medications	0.094	0.013	2.51E-13	2.46E-11	0.075	0.086	0.379	0.949
Anilides medications		Medications	-0.010	0.011	0.371	0.906	-0.103	0.068	0.130	0.787
Anti-inflammatory and antirheumatic products, non-steroids		Medications	-0.023	0.011	0.035	0.402	-0.105	0.077	0.177	0.825
Antidepressant medications		Medications	0.043	0.016	0.009	0.151	0.009	0.155	0.955	0.995
Antiglaucoma preparations and miotics		Medications	0.004	0.041	0.928	0.988	-0.098	0.225	0.663	0.991
Antihistamines for systemic use		Medications	0.009	0.025	0.728	0.977	0.133	0.157	0.397	0.949
Antihypertensive medications		Medications	-0.010	0.038	0.787	0.979	-0.162	0.177	0.360	0.946
Antimigraine preparations		Medications	-0.068	0.040	0.090	0.594	-1.971	1.018	0.053	0.706
Antithrombotic agents		Medications	0.152	0.012	1.50E-34	2.75E-32	-0.055	0.090	0.538	0.967
Beta blocking agents		Medications	0.149	0.017	7.36E-19	9.84E-17	-0.022	0.128	0.867	0.991
Calcium channel blockers		Medications	0.061	0.017	0.000	0.014	0.048	0.079	0.542	0.967
Diuretic medications		Medications	0.022	0.017	0.178	0.719	0.079	0.088	0.368	0.946
Drugs affecting bone structure and mineralization		Medications	-0.011	0.034	0.757	0.979	0.030	0.376	0.936	0.995
Drugs for peptic ulcer and gastro-esophageal reflux disease		Medications	0.028	0.014	0.044	0.457	0.119	0.102	0.243	0.916
Drugs used in diabetes		Medications	0.091	0.024	0.000	0.006	0.081	0.103	0.431	0.949
Glucocorticoid medications		Medications	0.014	0.022	0.531	0.935	0.046	0.168	0.782	0.991
HMG CoA reductase inhibitors		Medications	0.228	0.012	1.13E-79	3.33E-77	0.236	0.086	0.006	0.307
Immunosuppressant medications		Medications	0.005	0.046	0.914	0.987	-0.300	0.387	0.439	0.949
Opiod medications		Medications	0.035	0.020	0.072	0.550	-0.281	0.138	0.042	0.706
Salicylic acid and derivatives		Medications	0.145	0.013	1.09E-29	1.78E-27	-0.065	0.092	0.478	0.964
Thyroid preparations		Medications	0.010	0.019	0.598	0.964	0.119	0.181	0.513	0.967
Vasodilators used in cardiac diseases		Medications	0.302	0.037	2.05E-16	2.16E-14	0.227	0.348	0.513	0.967
Acute reaction to stress	300.8	Mental And Behavioral Disord	-0.419	0.327	0.200	0.741				
Addicted to Medication		Mental And Behavioral Disord	0.091	0.083	0.277	0.827				
Addiction behavior		Mental And Behavioral Disord	0.092	0.064	0.154	0.694				
Adjustment reaction	304	Mental And Behavioral Disord	-0.009	0.174	0.960	0.994				
Agorophobia, social phobia, and panic disorder	300.12	Mental And Behavioral Disord	0.122	0.193	0.526	0.935				
Alcohol addiction		Mental And Behavioral Disord	0.026	0.051	0.603	0.965				
Alcohol-related disorders	317	Mental And Behavioral Disord	0.065	0.084	0.439	0.922				
Alcoholic liver damage	317.11	Mental And Behavioral Disord	0.376	0.110	0.001	0.019				
Alcoholism	317.1	Mental And Behavioral Disord	0.149	0.097	0.126	0.646				
Alteration of consciousness	291.8	Mental And Behavioral Disord	0.062	0.156	0.689	0.977				
Altered mental status	292.4	Mental And Behavioral Disord	-0.109	0.076	0.153	0.694	-0.256	0.560	0.647	0.991
Alzheimer's disease	290.11	Mental And Behavioral Disord	-0.192	0.161	0.234	0.787				
Antisocial/borderline personality disorder	301.2	Mental And Behavioral Disord	0.167	0.264	0.526	0.935				
Anxiety		Mental And Behavioral Disord	-0.025	0.051	0.620	0.965				
Anxiety Criteria 1		Mental And Behavioral Disord	0.023	0.038	0.542	0.935	0.079	0.468	0.865	0.991
Anxiety Criteria 2		Mental And Behavioral Disord	-0.024	0.017	0.141	0.659	0.077	0.179	0.668	0.991
Anxiety Criteria 3		Mental And Behavioral Disord	-0.031	0.017	0.065	0.545	0.067	0.171	0.694	0.991
Anxiety Criteria 4		Mental And Behavioral Disord	-0.026	0.015	0.085	0.587	0.016	0.169	0.925	0.995
Anxiety Criteria 5		Mental And Behavioral Disord	-0.022	0.018	0.214	0.766	-0.189	0.220	0.390	0.949
Anxiety Criteria 6		Mental And Behavioral Disord	-0.029	0.016	0.071	0.549	-0.105	0.190	0.579	0.968
Anxiety disorder	300.1	Mental And Behavioral Disord	0.030	0.105	0.774	0.979				
Anxiety disorders	300	Mental And Behavioral Disord	0.086	0.204	0.675	0.977				
Aphasia/speech disturbance	292.1	Mental And Behavioral Disord	0.021	0.106	0.845	0.983				
Bipolar	296.1	Mental And Behavioral Disord	-0.148	0.111	0.183	0.720	-0.306	0.493	0.535	0.967
Bipolar Affective Disorder Criteria 1		Mental And Behavioral Disord	-0.004	0.016	0.775	0.979	-0.248	0.163	0.128	0.785
Bipolar Affective Disorder Criteria 3		Mental And Behavioral Disord	0.016	0.046	0.720	0.977	-0.458	0.546	0.402	0.949
Bipolar Affective Disorder Manifestation Criteria Score		Mental And Behavioral Disord	-0.001	0.002	0.680	0.977	-0.012	0.012	0.296	0.933
Current vs. ever smoking		Mental And Behavioral Disord	-0.015	0.016	0.327	0.874	-0.125	0.113	0.269	0.916
Current vs. never smoking		Mental And Behavioral Disord	-0.002	0.015	0.874	0.987	-0.143	0.096	0.135	0.787
Current vs. non-current alcohol drinking		Mental And Behavioral Disord	-0.041	0.024	0.087	0.589	-0.048	0.128	0.707	0.991
Delirium dementia and amnestic and other cognitive disorder	290	Mental And Behavioral Disord	-0.207	0.192	0.281	0.833				
Delirium due to conditions classified elsewhere	290.2	Mental And Behavioral Disord	0.121	0.100	0.228	0.785				
Dementias	290.1	Mental And Behavioral Disord	0.022	0.178	0.904	0.987				
Depression		Mental And Behavioral Disord	-0.016	0.017	0.326	0.874	-0.137	0.192	0.475	0.964
Depression Criteria 1		Mental And Behavioral Disord	-0.015	0.011	0.184	0.720	-0.054	0.115	0.637	0.991
Depression Criteria 2		Mental And Behavioral Disord	-0.001	0.015	0.930	0.989	-0.033	0.173	0.848	0.991
Depression Criteria 3		Mental And Behavioral Disord	-0.017	0.014	0.207	0.754	-0.228	0.148	0.124	0.774
Depression score		Mental And Behavioral Disord	-0.001	0.022	0.952	0.991	-0.157	0.218	0.471	0.964
Develomental delays and disorders	315	Mental And Behavioral Disord	-0.166	0.496	0.738	0.977				
Dissociative disorder	303.1	Mental And Behavioral Disord	-0.088	0.455	0.847	0.984				
Drug Addiction		Mental And Behavioral Disord	-0.253	0.126	0.045	0.460				
Dysthymic disorder	300.4	Mental And Behavioral Disord	0.637	0.463	0.169	0.713				
Ever Physicall Dependent on Alcohol		Mental And Behavioral Disord	0.016	0.117	0.889	0.987				
Ever vs. never drinking alcohol		Mental And Behavioral Disord	-0.072	0.025	0.004	0.091	-0.097	0.078	0.210	0.864
Ever vs. never smoking		Mental And Behavioral Disord	0.005	0.009	0.564	0.944	-0.050	0.067	0.451	0.960
Generalized anxiety disorder	300.11	Mental And Behavioral Disord	-0.215	0.318	0.498	0.927				
Hallucinations	292.6	Mental And Behavioral Disord	0.158	0.382	0.680	0.977				
Major depressive disorder	296.22	Mental And Behavioral Disord	0.045	0.132	0.736	0.977				
Mean packs of cigarettes smoked/day		Mental And Behavioral Disord	0.016	0.016	0.327	0.874	-0.143	0.113	0.204	0.853
Memory loss	292.3	Mental And Behavioral Disord	-0.157	0.165	0.342	0.885				
Mild cognitive impairment	292.2	Mental And Behavioral Disord	0.116	0.329	0.724	0.977				
Mood disorders	296	Mental And Behavioral Disord	0.503	0.402	0.211	0.761				
Neurological disorders	292	Mental And Behavioral Disord	0.169	0.144	0.242	0.790				
Obsessive-compulsive disorders	300.3	Mental And Behavioral Disord	-0.402	0.487	0.409	0.913				
Ongoing Addiction Behavior		Mental And Behavioral Disord	-0.228	0.132	0.084	0.587				

Ongoing Alcohol Addiction		Mental And Behavioral Disord	0.029	0.103	0.780	0.979				
Ongoing Behavioral or Misc Addiction		Mental And Behavioral Disord	0.010	0.033	0.754	0.979	-0.222	0.406	0.584	0.971
Ongoing Drug Addiction		Mental And Behavioral Disord	-0.721	0.433	0.095	0.605				
Ongoing Medication Addiction		Mental And Behavioral Disord	-0.164	0.175	0.350	0.896				
Other persistent mental disorders due to conditions classified	290.3	Mental And Behavioral Disord	1.038	0.422	0.014	0.203				
Pack-years of smoking		Mental And Behavioral Disord	0.012	0.008	0.130	0.646	-0.156	0.072	0.029	0.619
Paranoid disorders	295.2	Mental And Behavioral Disord	0.182	0.210	0.385	0.906				
Personality disorders	301	Mental And Behavioral Disord	0.233	0.259	0.369	0.906				
Psychogenic disorder	303.3	Mental And Behavioral Disord	0.223	0.140	0.111	0.624				
Psychosis	295.3	Mental And Behavioral Disord	-0.106	0.249	0.670	0.977				
Schizophrenia	295.1	Mental And Behavioral Disord	-0.003	0.146	0.984	0.997	-0.580	0.445	0.192	0.830
Sexual and gender identity disorders	302	Mental And Behavioral Disord	0.196	0.205	0.338	0.881				
Somatoform disorder	303.4	Mental And Behavioral Disord	-0.026	0.272	0.924	0.987				
Specific nonpsychotic mental disorders due to brain damage	291.4	Mental And Behavioral Disord	-0.105	0.336	0.755	0.979				
Speech and language disorder	315.2	Mental And Behavioral Disord	0.586	0.459	0.202	0.746				
Substance addiction and disorders	316	Mental And Behavioral Disord	-0.740	0.376	0.049	0.482				
Swelling, mass, or lump in head and neck [Space-occupying	293.1	Mental And Behavioral Disord	-0.014	0.095	0.886	0.987	0.872	0.461	0.059	0.706
Symptoms involving head and neck	293	Mental And Behavioral Disord	-0.032	0.068	0.639	0.968	-0.306	0.419	0.465	0.964
Tension headache	306.9	Mental And Behavioral Disord	0.216	0.133	0.106	0.621				
Transient mental disorders due to conditions classified elsewl	291.1	Mental And Behavioral Disord	-0.036	0.251	0.887	0.987				
Vascular dementia	290.16	Mental And Behavioral Disord	-0.697	0.307	0.023	0.301				
Years smoked cigarettes		Mental And Behavioral Disord	0.014	0.098	0.889	0.987	-1.179	0.766	0.124	0.774
Acquired deformities of finger	736.2	Musculoskeletal	-0.154	0.180	0.392	0.906				
Acquired foot deformities	735	Musculoskeletal	0.163	0.188	0.385	0.906				
Acquired spondylolisthesis	738.4	Musculoskeletal	0.066	0.079	0.400	0.906	-0.201	0.460	0.661	0.991
Acquired toe deformities	735.2	Musculoskeletal	-0.095	0.068	0.163	0.707				
Acute osteomyelitis	710.11	Musculoskeletal	0.323	0.443	0.466	0.925				
Ankylosing spondylitis	715.2	Musculoskeletal	-0.308	0.219	0.160	0.703				
Ankylosis of joint	741.1	Musculoskeletal	0.228	0.309	0.460	0.925				
Arthropathy NOS	716.9	Musculoskeletal	-0.004	0.018	0.825	0.979	0.022	0.135	0.870	0.991
Arthropathy associated with infections	711	Musculoskeletal	-0.382	0.486	0.431	0.920				
Arthropathy associated with other disorders classified elsewh	713	Musculoskeletal	0.002	0.339	0.996	1.000				
Articular cartilage disorder	742.8	Musculoskeletal	-0.102	0.118	0.390	0.906				
Aseptic necrosis of bone	733.4	Musculoskeletal	0.038	0.148	0.799	0.979				
Body fat %		Musculoskeletal	-0.043	0.026	0.103	0.621	0.252	0.173	0.146	0.796
Body mass index		Musculoskeletal	0.007	0.004	0.111	0.624	0.040	0.029	0.160	0.809
Body surface area		Musculoskeletal	0.000	0.002	0.943	0.990	0.005	0.034	0.884	0.995
Bursitis	726.3	Musculoskeletal	0.006	0.086	0.949	0.990				
Bursitis disorders	727.2	Musculoskeletal	-0.353	0.273	0.195	0.736				
Calcaneal spur; Exostosis NOS	726.4	Musculoskeletal	-0.570	0.406	0.161	0.703				
Chronic osteomyelitis	710.12	Musculoskeletal	-0.075	0.224	0.736	0.977				
Contracture of joint	739	Musculoskeletal	-0.255	0.157	0.104	0.621				
Contracture of palmar fascia [Dupuytren's disease]	728.71	Musculoskeletal	0.004	0.040	0.919	0.987				
Contracture of tendon (sheath)	727.7	Musculoskeletal	0.023	0.241	0.924	0.987				
Cyst of bone	733.2	Musculoskeletal	-0.257	0.304	0.398	0.906				
Degeneration of intervertebral disc	722.6	Musculoskeletal	0.084	0.059	0.155	0.694	-0.175	0.397	0.659	0.991
Difficulty in walking	741.3	Musculoskeletal	0.041	0.400	0.919	0.987				
Disorders of muscle, ligament, and fascia	728	Musculoskeletal	0.153	0.234	0.512	0.935				
Displacement of intervertebral disc	722.1	Musculoskeletal	-0.052	0.175	0.767	0.979				
Enthesopathy	726.1	Musculoskeletal	-0.009	0.030	0.776	0.979	0.095	0.205	0.643	0.991
Fasciitis	728.7	Musculoskeletal	-0.124	0.144	0.388	0.906				
Flat foot	735.1	Musculoskeletal	-0.137	0.231	0.553	0.939				
Ganglion and cyst of synovium, tendon, and bursa	727.4	Musculoskeletal	0.020	0.047	0.671	0.977	-0.013	0.271	0.961	0.996
Hallux rigidus	735.23	Musculoskeletal	-0.009	0.067	0.889	0.987				
Hallux valgus (Bunion)	735.3	Musculoskeletal	-0.025	0.032	0.440	0.922	-0.443	0.236	0.061	0.706
Hammer toe (acquired)	735.21	Musculoskeletal	0.189	0.071	0.008	0.140				
Hand grip strength		Musculoskeletal	-0.030	0.033	0.357	0.900	0.308	0.250	0.218	0.875
Hand preference		Musculoskeletal	-0.005	0.014	0.716	0.977	-0.149	0.104	0.153	0.796
Heel bone mineral density T-score		Musculoskeletal	-0.004	0.005	0.363	0.902	0.054	0.039	0.169	0.809
Height		Musculoskeletal	-0.028	0.028	0.306	0.862	0.196	0.183	0.284	0.922
Hemarthrosis	741.5	Musculoskeletal	0.397	0.212	0.061	0.538				
Hip circumference		Musculoskeletal	-0.001	0.004	0.891	0.987	0.028	0.028	0.317	0.946
Impedance of whole body		Musculoskeletal	-0.748	0.280	0.008	0.138	-2.262	2.041	0.268	0.916
Infective connective tissue disorders	712	Musculoskeletal	-0.755	0.433	0.081	0.587				
Intervertebral disc disorder with myelopathy	722.7	Musculoskeletal	-0.012	0.125	0.923	0.987	-0.019	0.567	0.973	0.996
Joint effusions	741.4	Musculoskeletal	0.009	0.084	0.915	0.987	0.178	0.474	0.707	0.991
Juvenile osteochondrosis	732.1	Musculoskeletal	0.396	0.247	0.108	0.624				
Kyphoscoliosis and scoliosis	737.3	Musculoskeletal	-0.010	0.138	0.944	0.990				
Kyphosis (acquired)	737.1	Musculoskeletal	0.142	0.344	0.680	0.977				
Laxity of ligament or hypermobility syndrome	728.2	Musculoskeletal	-0.023	0.153	0.878	0.987				
Loose body in joint	742.1	Musculoskeletal	-0.344	0.218	0.115	0.626				
Malunion and nonunion of fracture	733.8	Musculoskeletal	0.035	0.071	0.628	0.965				
Osteitis deformans [Paget's disease of bone]	731.1	Musculoskeletal	-0.748	0.475	0.115	0.626				
Osteoarthritis; localized	740.1	Musculoskeletal	-0.026	0.023	0.265	0.813	-0.057	0.285	0.840	0.991
Osteoarthrosis NOS	740.9	Musculoskeletal	-0.028	0.051	0.586	0.958	0.153	0.369	0.678	0.991
Osteoarthrosis, generalized	740.2	Musculoskeletal	0.132	0.127	0.300	0.861				
Osteoarthrosis, localized, primary	740.11	Musculoskeletal	0.001	0.029	0.980	0.997	0.064	0.237	0.786	0.991
Osteoarthrosis, localized, secondary	740.12	Musculoskeletal	-0.014	0.145	0.922	0.987				
Osteochondritis dissecans	732.7	Musculoskeletal	-1.265	0.734	0.085	0.587				
Osteochondropathies	732	Musculoskeletal	0.279	0.237	0.239	0.790				
Osteomyelitis, periostitis, and other infections involving bone	710	Musculoskeletal	-0.228	0.450	0.613	0.965				
Osteopenia or other disorder of bone and cartilage	743.9	Musculoskeletal	0.110	0.099	0.264	0.813				
Osteoporosis		Musculoskeletal	0.011	0.042	0.795	0.979	-0.369	0.304	0.225	0.890
Osteoporosis NOS	743.11	Musculoskeletal	-0.081	0.061	0.189	0.721				
Other acquired deformities of limbs	736	Musculoskeletal	-0.125	0.108	0.245	0.795				
Other acquired musculoskeletal deformity	738	Musculoskeletal	-0.065	0.160	0.684	0.977				
Other allied disorders of spine	721.8	Musculoskeletal	-0.194	0.241	0.422	0.919				
Other and unspecified disc disorder	722.9	Musculoskeletal	-0.074	0.072	0.305	0.862	0.108	0.385	0.779	0.991
Other arthropathies	716	Musculoskeletal	0.062	0.237	0.793	0.979				
Other derangement of joint	742.9	Musculoskeletal	-0.001	0.061	0.990	0.998	-0.403	0.420	0.337	0.946
Other disorders of bone and cartilage	733	Musculoskeletal	-0.066	0.095	0.490	0.925				
Other disorders of cervical region	723	Musculoskeletal	-0.018	0.123	0.885	0.987	-0.019	0.567	0.973	0.996
Other disorders of soft tissues	729	Musculoskeletal	-0.015	0.037	0.696	0.977	0.048	0.214	0.824	0.991
Other disorders of synovium, tendon, and bursa	727	Musculoskeletal	-0.035	0.107	0.746	0.979	-0.858	0.780	0.271	0.916
Other inflammatory spondylopathies	715	Musculoskeletal	-0.034	0.164	0.834	0.979				
Other symptoms referable to back	724.8	Musculoskeletal	0.188	0.407	0.644	0.968				
Other unspecified back disorders	724.9	Musculoskeletal	-0.036	0.118	0.757	0.979	0.065	0.513	0.899	0.995
Pain in joint	745	Musculoskeletal	0.018	0.030	0.539	0.935	0.361	0.176	0.040	0.706
Panniculitis	729.3	Musculoskeletal	-0.507	0.440	0.249	0.801				
Pathologic fracture	743.2	Musculoskeletal	0.139	0.196	0.478	0.925				
Pathologic fracture of vertebrae	743.21	Musculoskeletal	0.440	0.169	0.009	0.151				
Pathological, developmental or recurrent dislocation	742.2	Musculoskeletal	0.252	0.367	0.492	0.925				
Peripheral enthesopathies and allied syndromes	726	Musculoskeletal	0.014	0.027	0.599	0.964	0.109	0.167	0.514	0.967
Polymyalgia Rheumatica	717	Musculoskeletal	-0.457	0.225	0.042	0.453				
Postlaminectomy syndrome	722.8	Musculoskeletal	-0.106	0.309	0.731	0.977				
Pyogenic arthritis	711.1	Musculoskeletal	-0.404	0.148	0.006	0.115				
Reiter's disease	711.2	Musculoskeletal	-0.428	0.627	0.495	0.925				
Rheumatism, unspecified and fibrositis	729.1	Musculoskeletal	0.124	0.202	0.538	0.935				
Rheumatoid arthritis	714.1	Musculoskeletal	-0.021	0.058	0.723	0.977	-0.418	0.563	0.457	0.964

Rheumatoid arthritis and other inflammatory polyarthropathie	714	Musculoskeletal	-0.266	0.178	0.135	0.650				
Rupture of synovium	727.5	Musculoskeletal	0.008	0.109	0.939	0.990				
Rupture of tendon, nontraumatic	727.6	Musculoskeletal	0.113	0.133	0.397	0.906				
Sacroiliitis NEC	715.1	Musculoskeletal	-0.774	0.306	0.011	0.176				
Spinal stenosis	720	Musculoskeletal	0.030	0.045	0.503	0.928	-0.056	0.276	0.838	0.991
Spondylosis and allied disorders	721	Musculoskeletal	0.027	0.081	0.740	0.977				
Spondylosis with myelopathy	721.2	Musculoskeletal	0.012	0.188	0.950	0.990				
Spondylosis without myelopathy	721.1	Musculoskeletal	-0.002	0.051	0.964	0.994	0.247	0.302	0.414	0.949
Stiffness of joint	741.2	Musculoskeletal	0.044	0.115	0.702	0.977				
Stress fracture	743.4	Musculoskeletal	-0.217	0.551	0.694	0.977				
Symptoms and disorders of the joints	741	Musculoskeletal	-0.007	0.094	0.942	0.990				
Synoviopathy	726.2	Musculoskeletal	0.213	0.153	0.164	0.707				
Synovitis and tenosynovitis	727.1	Musculoskeletal	0.024	0.041	0.560	0.944	-0.171	0.272	0.530	0.967
Torticollis	723.1	Musculoskeletal	-0.042	0.503	0.933	0.990				
Unspecified monoarthritis	716.2	Musculoskeletal	0.003	0.022	0.895	0.987	-0.018	0.161	0.911	0.995
Unspecified osteomyelitis	710.19	Musculoskeletal	-0.085	0.137	0.534	0.935				
Villonodular synovitis	741.6	Musculoskeletal	-0.115	0.371	0.757	0.979				
Waist circumference		Musculoskeletal	0.117	0.051	0.023	0.301	0.283	0.355	0.426	0.949
Waist/hip ratio		Musculoskeletal	0.001	0.000	0.001	0.037	0.000	0.002	0.893	0.995
Weight		Musculoskeletal	0.005	0.004	0.217	0.767	0.048	0.029	0.096	0.754
Whole body fat mass		Musculoskeletal	-0.014	0.033	0.682	0.977	0.371	0.231	0.108	0.766
Whole body fat-free mass		Musculoskeletal	0.003	0.003	0.234	0.787	0.036	0.020	0.067	0.706
Whole body water mass		Musculoskeletal	0.004	0.003	0.196	0.736	0.034	0.020	0.082	0.732
Benign neoplasm of adrenal gland	227.1	Neoplasms	-0.169	0.221	0.445	0.922				
Benign neoplasm of bone and articular cartilage	213	Neoplasms	-0.313	0.171	0.067	0.545				
Benign neoplasm of brain, cranial nerves, meninges	225.1	Neoplasms	0.014	0.092	0.875	0.987				
Benign neoplasm of colon	208	Neoplasms	-0.013	0.016	0.428	0.919	-0.471	0.141	0.001	0.086
Benign neoplasm of eye	224	Neoplasms	0.027	0.326	0.934	0.990				
Benign neoplasm of kidney and other urinary organs	223	Neoplasms	-0.323	0.206	0.117	0.626				
Benign neoplasm of lip, oral cavity, and pharynx	210	Neoplasms	-0.057	0.082	0.488	0.925				
Benign neoplasm of lymph nodes	229.1	Neoplasms	-0.276	0.548	0.615	0.965				
Benign neoplasm of male genital organs	222	Neoplasms	-0.152	0.232	0.513	0.935				
Benign neoplasm of other female genital organs	221	Neoplasms	0.306	0.184	0.097	0.605				
Benign neoplasm of other parts of digestive system	211	Neoplasms	-0.020	0.040	0.613	0.965	0.246	0.304	0.417	0.949
Benign neoplasm of ovary	220	Neoplasms	0.077	0.069	0.265	0.813	0.990	0.453	0.029	0.619
Benign neoplasm of parathyroid gland	227.2	Neoplasms	-0.212	0.127	0.095	0.604				
Benign neoplasm of pituitary gland and craniopharyngeal duc	227.3	Neoplasms	-0.232	0.156	0.136	0.650	-0.455	0.635	0.473	0.964
Benign neoplasm of respiratory and intrathoracic organs	212	Neoplasms	0.039	0.130	0.765	0.979				
Benign neoplasm of skin	216	Neoplasms	0.031	0.028	0.272	0.816	0.187	0.303	0.537	0.967
Benign neoplasm of spinal cord, meninges	225.2	Neoplasms	0.137	0.305	0.653	0.973				
Benign neoplasm of thyroid glands	226	Neoplasms	-0.108	0.162	0.506	0.932				
Benign neoplasm of unspecified sites	229	Neoplasms	0.022	0.056	0.693	0.977	-0.319	0.549	0.562	0.967
Biliary cancer		Neoplasms	-0.104	0.135	0.444	0.922				
Bladder cancer		Neoplasms	-0.013	0.069	0.855	0.987				
Bone cancer	170.1	Neoplasms	-0.128	0.210	0.544	0.935				
Bone marrow or stem cell transplant	860	Neoplasms	0.256	0.367	0.486	0.925				
Brain cancer		Neoplasms	0.001	0.098	0.994	1.000				
Breast cancer		Neoplasms	-0.013	0.024	0.583	0.958	-0.094	0.216	0.664	0.991
Breast cancer [female]	174.1	Neoplasms	-0.080	0.060	0.185	0.720	0.096	0.384	0.803	0.991
Cancer of bladder	189.2	Neoplasms	0.062	0.087	0.477	0.925				
Cancer of brain	191.11	Neoplasms	0.066	0.095	0.484	0.925				
Cancer of brain and nervous system	191.1	Neoplasms	0.217	0.334	0.515	0.935				
Cancer of bronchus; lung	165.1	Neoplasms	-0.026	0.046	0.567	0.944	0.491	0.366	0.180	0.825
Cancer of connective tissue	170.2	Neoplasms	-0.195	0.137	0.155	0.694				
Cancer of esophagus	150	Neoplasms	0.069	0.076	0.363	0.902				
Cancer of eye	190	Neoplasms	0.112	0.178	0.530	0.935				
Cancer of hypopharynx	149.3	Neoplasms	0.230	0.289	0.427	0.919				
Cancer of intrathoracic organs	164	Neoplasms	-0.749	0.433	0.084	0.587				
Cancer of larynx	149.4	Neoplasms	-0.006	0.143	0.967	0.994				
Cancer of larynx, pharynx, nasal cavities	149	Neoplasms	-0.509	0.407	0.211	0.761				
Cancer of lip	145.1	Neoplasms	0.222	0.298	0.456	0.925				
Cancer of liver and intrahepatic bile duct	155	Neoplasms	-0.263	0.152	0.084	0.587				
Cancer of major salivary glands	145.3	Neoplasms	0.101	0.219	0.644	0.968				
Cancer of mouth	145	Neoplasms	0.010	0.185	0.956	0.994				
Cancer of nasopharynx	149.2	Neoplasms	0.232	0.366	0.526	0.935				
Cancer of of nasal cavities	149.9	Neoplasms	0.031	0.302	0.917	0.987				
Cancer of oropharynx	149.1	Neoplasms	0.020	0.138	0.884	0.987				
Cancer of other endocrine glands	194	Neoplasms	0.322	0.261	0.217	0.767				
Cancer of other female genital organs	184	Neoplasms	-0.341	0.250	0.172	0.713				
Cancer of other female genital organs (excluding uterus and c	184.2	Neoplasms	-0.100	0.145	0.492	0.925				
Cancer of other lymphoid, histiocytic tissue	202	Neoplasms	-0.271	0.234	0.247	0.798				
Cancer of other male genital organs	187	Neoplasms	-0.187	0.391	0.633	0.965				
Cancer of prostate	185	Neoplasms	0.034	0.029	0.251	0.805	-0.198	0.178	0.268	0.916
Cancer of stomach	151	Neoplasms	0.036	0.089	0.685	0.977				
Cancer of the gums	145.4	Neoplasms	-0.292	0.414	0.481	0.925				
Cancer of the mouth floor	145.5	Neoplasms	-0.014	0.356	0.969	0.994				
Cancer of tongue	145.2	Neoplasms	-0.069	0.134	0.607	0.965				
Cancer of urinary organs (incl. kidney and bladder)	189	Neoplasms	-0.230	0.416	0.581	0.958				
Cancer within the respiratory system	165	Neoplasms	-0.153	0.150	0.306	0.862				
Cancer, suspected or other	195	Neoplasms	-0.022	0.097	0.820	0.979				
Carcinoma in situ of skin	172.3	Neoplasms	-0.009	0.084	0.916	0.987				
Cervical cancer	180.1	Neoplasms	-0.130	0.173	0.452	0.925				
Cervical cancer		Neoplasms	-0.100	0.098	0.311	0.863				
Cervical intraepithelial neoplasia [CIN] [Cervical dysplasia]	180.3	Neoplasms	0.106	0.062	0.090	0.594	-0.530	0.332	0.110	0.766
Chemotherapy	197	Neoplasms	0.109	0.276	0.692	0.977				
Colon cancer	153.2	Neoplasms	-0.053	0.041	0.197	0.736	-0.350	0.348	0.314	0.943
Colorectal cancer	153	Neoplasms	-0.103	0.422	0.807	0.979				
Colorectal cancer		Neoplasms	-0.008	0.035	0.818	0.979	0.066	0.277	0.811	0.991
Esophageal cancer		Neoplasms	0.119	0.086	0.165	0.711				
Head cancer		Neoplasms	0.003	0.068	0.962	0.994				
Hemangioma and lymphangioma, any site	228	Neoplasms	-0.044	0.072	0.541	0.935				
Hodgkin's disease	201	Neoplasms	-0.009	0.178	0.960	0.994				
Kidney cancer		Neoplasms	0.133	0.067	0.046	0.463				
Large cell lymphoma	202.24	Neoplasms	0.123	0.087	0.154	0.694				
Leukemia	204	Neoplasms	-0.177	0.196	0.366	0.905				
Leukemia cancer		Neoplasms	0.013	0.072	0.854	0.986				
Lipoma	214	Neoplasms	-0.174	0.105	0.098	0.607	-0.084	0.510	0.869	0.991
Lipoma of skin and subcutaneous tissue	214.1	Neoplasms	-0.013	0.037	0.719	0.977	0.064	0.171	0.711	0.991
Liver cancer		Neoplasms	0.592	0.164	0.000	0.010				
Lung cancer		Neoplasms	0.052	0.046	0.267	0.813	0.435	0.395	0.271	0.916
Lymphoid leukemia	204.1	Neoplasms	-0.494	0.482	0.305	0.862				
Lymphoid leukemia, acute	204.11	Neoplasms	-0.493	0.359	0.170	0.713				
Lymphoid leukemia, chronic	204.12	Neoplasms	-0.052	0.125	0.679	0.977				
Malignant neoplasm of bladder	189.21	Neoplasms	-0.024	0.049	0.628	0.965				
Malignant neoplasm of female breast	174.11	Neoplasms	-0.001	0.025	0.973	0.994	-0.047	0.202	0.817	0.991
Malignant neoplasm of gallbladder and extrahepatic bile duc	159.3	Neoplasms	0.102	0.146	0.483	0.925				
Malignant neoplasm of head, face, and neck	195.3	Neoplasms	0.326	0.353	0.355	0.897				
Malignant neoplasm of kidney, except pelvis	189.11	Neoplasms	0.190	0.068	0.005	0.101				
Malignant neoplasm of liver, primary	155.1	Neoplasms	0.491	0.158	0.002	0.049				

Malignant neoplasm of other and ill-defined sites within the (	159	Neoplasms	-0.289	0.266	0.278	0.830				
Malignant neoplasm of other urinary organs	189.4	Neoplasms	-0.198	0.209	0.343	0.885				
Malignant neoplasm of ovary	184.11	Neoplasms	-0.010	0.077	0.898	0.987				
Malignant neoplasm of ovary and other uterine adnexa	184.1	Neoplasms	-0.047	0.223	0.834	0.979				
Malignant neoplasm of rectum, rectosigmoid junction, and ar	153.3	Neoplasms	0.011	0.049	0.823	0.979	-0.020	0.464	0.966	0.996
Malignant neoplasm of renal pelvis	189.12	Neoplasms	-0.185	0.268	0.492	0.925				
Malignant neoplasm of retroperitoneum and peritoneum	159.4	Neoplasms	-0.083	0.168	0.623	0.965				
Malignant neoplasm of small intestine, including duodenum	159.2	Neoplasms	-0.019	0.164	0.907	0.987				
Malignant neoplasm of testis	187.2	Neoplasms	-0.123	0.165	0.454	0.925				
Malignant neoplasm of unspecified male genital organ	187.1	Neoplasms	-0.653	0.357	0.067	0.545				
Malignant neoplasm of uterus	182	Neoplasms	0.107	0.062	0.085	0.587	-0.187	0.559	0.738	0.991
Malignant neoplasm, other	195.1	Neoplasms	-0.007	0.059	0.910	0.987	0.258	0.450	0.567	0.968
Manlignant and unknown neoplasms of brain and nervous sy	191	Neoplasms	-0.343	0.184	0.063	0.539				
Melanoma cancer		Neoplasms	-0.094	0.043	0.031	0.366				
Melanomas of skin	172.11	Neoplasms	-0.083	0.044	0.058	0.525				
Monocytic leukemia	204.3	Neoplasms	0.310	0.324	0.338	0.881				
Multiple myeloma	204.4	Neoplasms	0.063	0.085	0.455	0.925	0.623	0.360	0.084	0.732
Myeloid leukemia	204.2	Neoplasms	-0.422	0.485	0.384	0.906				
Myeloid leukemia, acute	204.21	Neoplasms	0.007	0.124	0.957	0.994				
Myeloid leukemia, chronic	204.22	Neoplasms	0.047	0.210	0.822	0.979				
Myeloma cancer		Neoplasms	-0.033	0.100	0.739	0.977				
Myeloproliferative disease	200	Neoplasms	0.065	0.096	0.495	0.925				
Neoplasm of uncertain behavior	199	Neoplasms	0.050	0.072	0.482	0.925	0.535	0.359	0.136	0.787
Neoplasm of uncertain behavior of breast	174.3	Neoplasms	0.064	0.247	0.794	0.979				
Neoplasm of uncertain behavior of male genital organs	187.8	Neoplasms	0.138	0.239	0.564	0.944				
Neoplasm of uncertain behavior of skin	173	Neoplasms	0.330	0.180	0.067	0.545				
Neoplasm of unspecified nature of digestive system	158	Neoplasms	-0.047	0.075	0.529	0.935	0.162	0.412	0.693	0.991
Neurofibromatosis	199.4	Neoplasms	-0.232	0.390	0.551	0.939				
Nevus, non-neoplastic	217.1	Neoplasms	-0.173	0.147	0.238	0.788				
Nodular lymphoma	202.21	Neoplasms	-0.123	0.129	0.342	0.885				
Non cancer		Neoplasms	0.067	0.054	0.214	0.766	-0.001	0.505	0.998	0.999
Non-Hodgkins lymphoma	202.2	Neoplasms	-0.021	0.064	0.745	0.979	-0.476	0.633	0.452	0.960
Other benign neoplasm of connective and other soft tissue	215	Neoplasms	0.115	0.075	0.123	0.646	-0.047	0.464	0.919	0.995
Other benign neoplasm of uterus	218.2	Neoplasms	0.020	0.178	0.912	0.987				
Other non-epithelial cancer of skin	172.2	Neoplasms	-0.029	0.021	0.162	0.706				
Ovarian cancer		Neoplasms	-0.076	0.074	0.304	0.862				
Pancreatic cancer	157	Neoplasms	0.028	0.082	0.730	0.977				
Pancreatic cancer		Neoplasms	0.051	0.085	0.549	0.937				
Polycythemia vera	200.1	Neoplasms	0.090	0.139	0.516	0.935				
Prostate cancer		Neoplasms	0.044	0.027	0.103	0.621	-0.032	0.173	0.854	0.991
Secondary malignancy of bone	198.6	Neoplasms	0.044	0.068	0.521	0.935	-0.045	0.571	0.938	0.995
Secondary malignancy of brain/spine	198.5	Neoplasms	-0.113	0.099	0.252	0.806				
Secondary malignancy of lymph nodes	198.1	Neoplasms	-0.023	0.074	0.755	0.979				
Secondary malignancy of respiratory organs	198.2	Neoplasms	-0.005	0.073	0.940	0.990	-0.079	0.515	0.878	0.993
Secondary malignant neoplasm	198	Neoplasms	-0.259	0.143	0.070	0.545				
Secondary malignant neoplasm of digestive systems	198.3	Neoplasms	0.042	0.083	0.609	0.965				
Secondary malignant neoplasm of liver	198.4	Neoplasms	-0.045	0.072	0.536	0.935	0.321	0.527	0.542	0.967
Secondary malignant neoplasm of skin	198.7	Neoplasms	-0.059	0.219	0.789	0.979				
Stomach cancer		Neoplasms	0.010	0.105	0.927	0.988				
Testicular cancer		Neoplasms	-0.078	0.109	0.472	0.925				
Thyroid cancer	193	Neoplasms	0.227	0.118	0.056	0.514				
Thyroid cancer		Neoplasms	0.067	0.107	0.529	0.935				
Uterine cancer		Neoplasms	0.087	0.061	0.149	0.691	0.098	0.514	0.848	0.991
Uterine leiomyoma	218.1	Neoplasms	-0.005	0.033	0.874	0.987	0.019	0.102	0.849	0.991
Vascular hamartomas and non-neoplastic nevi	217	Neoplasms	0.264	0.335	0.430	0.919				
Abnormal involuntary movements	350.1	Neurological	-0.023	0.130	0.860	0.987				
Abnormality of gait	350.2	Neurological	-0.066	0.098	0.502	0.928				
Acute (transverse) myelitis	323.2	Neurological	0.266	0.274	0.333	0.876				
Acute pain	338.1	Neurological	0.225	0.167	0.177	0.719				
Anterior horn cell disease	334.2	Neurological	-0.031	0.159	0.845	0.983				
Back pain		Neurological	0.007	0.012	0.553	0.939	0.058	0.072	0.418	0.949
Cerebral cysts	348.4	Neurological	-0.026	0.324	0.937	0.990				
Cerebral degeneration, unspecified	331.9	Neurological	0.410	0.247	0.097	0.605				
Cerebral edema and compression of brain	348.2	Neurological	-0.103	0.271	0.702	0.977				
Chronic pain	338.2	Neurological	-0.312	0.219	0.153	0.694				
Circadian rhythm sleep disorder	327.6	Neurological	0.221	0.517	0.669	0.977				
Coma	348.7	Neurological	0.283	0.238	0.233	0.787				
Convulsions	345.3	Neurological	0.012	0.059	0.832	0.979	0.155	0.474	0.743	0.991
Degenerative disease of the spinal cord	334	Neurological	-0.036	0.113	0.751	0.979				
Disorders of other cranial nerves	352	Neurological	-0.269	0.415	0.518	0.935				
Disorders of the autonomic nervous system	337	Neurological	-0.112	0.288	0.698	0.977				
Disturbances of sensation of smell and taste	350.6	Neurological	0.268	0.411	0.514	0.935				
Encephalitis	323	Neurological	-0.186	0.169	0.270	0.813				
Encephalitis, non-infectious	323.8	Neurological	0.393	0.375	0.295	0.854				
Encephalopathy, not elsewhere classified	348.8	Neurological	0.162	0.307	0.598	0.964				
Epilepsy	345.1	Neurological	-0.283	0.180	0.116	0.626				
Epilepsy, recurrent seizures, convulsions	345	Neurological	-0.063	0.090	0.484	0.925				
Essential tremor	333.1	Neurological	0.072	0.401	0.858	0.987				
Extrapyramidal disease and abnormal movement disorders	333	Neurological	0.186	0.246	0.448	0.923				
Facial nerve disorders [CN7]	352.2	Neurological	-0.026	0.109	0.811	0.979				
Facial pain		Neurological	-0.024	0.047	0.620	0.965	0.316	0.294	0.281	0.922
Fluid intelligence		Neurological	-0.010	0.008	0.182	0.720	0.040	0.041	0.329	0.946
General pain		Neurological	-0.013	0.039	0.732	0.977	-0.066	0.174	0.703	0.991
Generalized convulsive epilepsy	345.11	Neurological	-0.242	0.154	0.116	0.626				
Headache		Neurological	-0.030	0.016	0.055	0.508	0.012	0.108	0.915	0.995
Hemiplegia	342	Neurological	0.027	0.118	0.816	0.979				
Hereditary and idiopathic peripheral neuropathy	356	Neurological	-0.119	0.321	0.711	0.977				
Hip pain		Neurological	0.021	0.016	0.177	0.719	-0.053	0.108	0.621	0.986
Hydrocephalus	331.1	Neurological	-0.250	0.183	0.173	0.713				
Infantile cerebral palsy	343	Neurological	-0.872	0.746	0.242	0.790				
Inflammatory and toxic neuropathy	357	Neurological	-0.041	0.094	0.659	0.977				
Jakob-Creutzfeldt disease	324.1	Neurological	0.201	0.513	0.696	0.977				
Knee pain		Neurological	0.026	0.012	0.029	0.355	-0.128	0.076	0.095	0.754
Lack of coordination	350.3	Neurological	-0.394	0.243	0.104	0.621				
Meningitis	320	Neurological	0.016	0.133	0.906	0.987				
Migrain with aura	340.1	Neurological	0.138	0.172	0.425	0.919				
Migraine	340	Neurological	-0.057	0.064	0.368	0.906	0.013	0.347	0.969	0.996
Multiple sclerosis	335	Neurological	0.086	0.095	0.364	0.902				
Muscular dystrophies	359.1	Neurological	0.237	0.310	0.444	0.922				
Myasthenia gravis	358.1	Neurological	0.282	0.206	0.170	0.713				
Myoclonus	333.2	Neurological	0.378	0.416	0.363	0.902				
Myopathy	359.2	Neurological	0.241	0.151	0.110	0.624				
Neck and shoulder pain		Neurological	0.019	0.012	0.117	0.626	-0.270	0.086	0.002	0.122
Nerve plexus lesions	353.1	Neurological	0.150	0.262	0.568	0.945				
Nerve root and plexus disorders	353	Neurological	-0.005	0.161	0.976	0.994				
Nerve root lesions	353.2	Neurological	0.446	0.346	0.197	0.736				
Nonspecific abnormal findings on radiological and other exa	346.1	Neurological	-0.712	0.532	0.180	0.720				
Numeric memory		Neurological	0.030	0.018	0.091	0.599	0.209	0.218	0.337	0.946
Organic or persistent insomnia	327.41	Neurological	-0.021	0.300	0.944	0.990				

Other CNS infection and poliomyelitis	324	Neurological	-0.182	0.261	0.485	0.925				
Other and unspecified disorders of the nervous system	349	Neurological	-0.127	0.185	0.494	0.925				
Other cerebral degenerations	331	Neurological	0.052	0.236	0.827	0.979				
Other conditions of brain	348	Neurological	-0.006	0.282	0.983	0.997				
Other conditions of brain, NOS	348.9	Neurological	-0.127	0.168	0.449	0.923				
Other degenerative diseases of the basal ganglia	333.8	Neurological	-0.118	0.393	0.763	0.979				
Other demyelinating diseases of central nervous system	341	Neurological	-0.157	0.193	0.417	0.919				
Other headache syndromes	339	Neurological	0.055	0.033	0.090	0.594	-0.259	0.188	0.167	0.809
Other paralytic syndromes	344	Neurological	0.051	0.143	0.720	0.977				
Other peripheral nerve disorders	351	Neurological	-0.028	0.023	0.222	0.779	-0.034	0.163	0.837	0.991
Parkinson's disease	332	Neurological	-0.099	0.115	0.387	0.906				
Partial epilepsy	345.12	Neurological	-0.084	0.201	0.676	0.977				
Peripheral autonomic neuropathy	337.1	Neurological	-0.035	0.355	0.921	0.987				
Phlebitis and thrombophlebitis of intracranial venous sinuses	325	Neurological	-0.299	0.414	0.469	0.925				
Prospective memory		Neurological	-0.029	0.019	0.134	0.650	0.029	0.080	0.722	0.991
Reaction time		Neurological	-0.003	0.004	0.508	0.933	-0.032	0.028	0.253	0.916
Sleep apnea	327.3	Neurological	0.101	0.049	0.040	0.439	-0.252	0.289	0.383	0.949
Sleep disorders	327	Neurological	-0.088	0.085	0.299	0.861				
Spinocerebellar disease	334.1	Neurological	0.378	0.287	0.188	0.721				
Stomach pain		Neurological	0.016	0.021	0.437	0.922	-0.093	0.119	0.437	0.949
Torsion dystonia	333.4	Neurological	-0.062	0.201	0.757	0.979				
Trigeminal nerve disorders [CN5]	352.1	Neurological	-0.050	0.143	0.729	0.977				
Visual memory		Neurological	0.003	0.004	0.420	0.919	-0.048	0.028	0.088	0.735
Abnormality of organs and soft tissues of pelvis complicating	654.1	Reproductive	-0.098	0.100	0.324	0.874	-0.772	0.542	0.154	0.796
Age at menarche		Reproductive	-0.010	0.010	0.319	0.874	0.039	0.074	0.602	0.977
Age at menopause		Reproductive	-0.007	0.008	0.362	0.902	0.095	0.054	0.082	0.732
Age first birth		Reproductive	0.007	0.007	0.297	0.858	0.005	0.046	0.921	0.995
Age last birth		Reproductive	0.030	0.034	0.383	0.906	0.246	0.272	0.365	0.946
Anemia during pregnancy	644	Reproductive	0.733	0.323	0.023	0.301				
Antepartum hemorrhage, abruptio placentae, and placenta pr	635.2	Reproductive	0.179	0.100	0.073	0.552	0.262	0.442	0.554	0.967
Bilateral oophorectomy		Reproductive	0.006	0.022	0.790	0.979	-0.278	0.158	0.078	0.732
Cervical incompetence	636.8	Reproductive	0.113	0.467	0.809	0.979				
Complications of labor and delivery NEC	669	Reproductive	0.023	0.065	0.720	0.977	0.229	0.248	0.357	0.946
Diabetes or abnormal glucose tolerance complicating pregnai	649.1	Reproductive	-0.162	0.287	0.574	0.952				
Early onset of delivery	636.2	Reproductive	0.014	0.187	0.942	0.990				
Early or threatened labor; hemorrhage in early pregnancy	636	Reproductive	0.088	0.085	0.302	0.862	0.190	0.337	0.573	0.968
Ectopic pregnancy	634.3	Reproductive	0.162	0.144	0.261	0.813				
Ever had hysterectomy		Reproductive	0.037	0.024	0.115	0.626	-0.045	0.137	0.743	0.991
Ever had stillbirth		Reproductive	0.020	0.013	0.128	0.646	-0.008	0.079	0.920	0.995
Excessive vomiting in pregnancy	643	Reproductive	-0.020	0.313	0.949	0.990				
Hemorrhage in early pregnancy	636.3	Reproductive	-0.069	0.093	0.458	0.925	0.597	0.375	0.111	0.766
Hyperemesis gravidarum	643.1	Reproductive	0.081	0.229	0.723	0.977				
Hypertension complicating pregnancy, childbirth, and the puc	642	Reproductive	0.095	0.099	0.336	0.879	0.404	0.417	0.332	0.946
Infections of genitourinary tract during pregnancy	647.1	Reproductive	0.181	0.223	0.417	0.919				
Infectious and parasitic complications affecting pregnancy	647	Reproductive	0.568	0.339	0.093	0.604				
Known or suspected fetal abnormality affecting management	655	Reproductive	0.035	0.049	0.476	0.925	0.200	0.205	0.331	0.946
Major puerperal infection	647.3	Reproductive	-0.946	0.749	0.207	0.754				
Maternal smoking		Reproductive	0.014	0.010	0.172	0.713	0.012	0.158	0.937	0.995
Miscarriage; stillbirth	634	Reproductive	0.074	0.046	0.108	0.624	-0.016	0.174	0.925	0.995
Missed abortion/Hydatidiform mole	634.1	Reproductive	0.060	0.078	0.441	0.922	0.203	0.340	0.551	0.967
Number of children fathered		Reproductive	0.000	0.006	0.964	0.994	-0.001	0.042	0.981	0.996
Other complications of pregnancy NEC	646	Reproductive	-0.009	0.063	0.889	0.987	0.063	0.248	0.798	0.991
Other complications of the puerperium NEC	674	Reproductive	0.126	0.217	0.562	0.944				
Other conditions or status of the mother complicating pregnai	649	Reproductive	-0.490	0.544	0.368	0.906				
Other perinatal conditions of fetus or newborn	656	Reproductive	-0.300	0.367	0.414	0.917				
Placenta previa and abruptio placenta	635.3	Reproductive	-0.053	0.146	0.714	0.977				
Preeclampsia and eclampsia	642.1	Reproductive	-0.299	0.194	0.124	0.646				
Problems associated with amniotic cavity and membranes	653	Reproductive	-0.020	0.087	0.815	0.979	0.225	0.381	0.555	0.967
Rhesus isoimmunization in pregnancy	654.2	Reproductive	-0.563	0.360	0.118	0.626				
Venous/cerebrovascular complications embolism in pregnanc	671	Reproductive	0.175	0.365	0.631	0.965				
Abnormal findings examination of lungs	514	Respiratory	0.053	0.057	0.354	0.897	-0.382	0.492	0.438	0.949
Acute and chronic tonsillitis	474	Respiratory	-0.216	0.133	0.105	0.621				
Acute bronchitis and bronchiolitis	483	Respiratory	0.185	0.154	0.228	0.785				
Acute laryngitis and tracheitis	465.4	Respiratory	-0.113	0.227	0.619	0.965				
Acute pharyngitis	465.2	Respiratory	-0.295	0.130	0.023	0.301				
Acute sinusitis	464	Respiratory	0.235	0.198	0.236	0.787				
Acute tonsillitis	474.1	Respiratory	-0.088	0.113	0.434	0.922	-0.158	0.460	0.732	0.991
Acute upper respiratory infections of multiple or unspecified s	465	Respiratory	0.068	0.089	0.445	0.922	0.274	0.402	0.495	0.967
Allergic rhinitis	476	Respiratory	-0.238	0.318	0.455	0.925				
Alpha-1 antritrypsin		Respiratory	0.400	0.391	0.307	0.862				
Alveolitis		Respiratory	0.328	0.388	0.397	0.906				
Asbestosis		Respiratory	0.028	0.312	0.928	0.988				
Asthma	495	Respiratory	0.066	0.050	0.181	0.720	0.284	0.259	0.271	0.916
Asthma		Respiratory	-0.001	0.026	0.962	0.994	-0.022	0.298	0.942	0.995
Asthma with exacerbation	495.2	Respiratory	0.081	0.162	0.618	0.965				
Bacterial pneumonia	480.1	Respiratory	-0.028	0.109	0.794	0.979				
Bronchiectasis	496.3	Respiratory	-0.042	0.091	0.643	0.968				
Bronchiectasis		Respiratory	0.030	0.090	0.738	0.977				
Bronchitis	497	Respiratory	-0.039	0.156	0.804	0.979				
Bronchopneumonia and lung abscess	480.5	Respiratory	-0.329	0.138	0.017	0.236				
Chronic airway obstruction	496	Respiratory	-0.122	0.115	0.290	0.845				
Chronic bronchitis	496.2	Respiratory	0.137	0.306	0.654	0.974				
Chronic bronchitis		Respiratory	-0.021	0.083	0.804	0.979				
Chronic laryngitis	473.1	Respiratory	-0.151	0.334	0.652	0.973				
Chronic obstructive pulmonary disease		Respiratory	0.000	0.072	0.997	1.000				
Chronic pharyngitis and nasopharyngitis	472	Respiratory	0.163	0.125	0.194	0.736				
Chronic sinusitis	475	Respiratory	0.007	0.066	0.918	0.987	-0.585	0.629	0.353	0.946
Chronic tonsillitis and adenoiditis	474.2	Respiratory	-0.043	0.089	0.628	0.965	0.100	0.437	0.819	0.991
Cough	512.8	Respiratory	0.014	0.065	0.826	0.979	-0.271	0.451	0.548	0.967
Diseases of the larynx and vocal cords	473	Respiratory	-0.046	0.066	0.481	0.925	-0.982	0.612	0.109	0.766
Disorders of diaphragm	513.8	Respiratory	0.018	0.326	0.956	0.994				
Emphysema	496.1	Respiratory	-0.011	0.150	0.940	0.990				
Emphysema		Respiratory	0.162	0.149	0.276	0.827				
Empyema and pneumothorax	506	Respiratory	0.139	0.089	0.116	0.626				
Epistaxis or throat hemorrhage	477	Respiratory	0.134	0.051	0.008	0.140	0.179	0.413	0.665	0.991
Extrinsic allergic alveolitis	500.1	Respiratory	0.006	0.301	0.985	0.997				
Forced expiratory volume Z score		Respiratory	0.005	0.005	0.311	0.863				
Forced expiratory volume/forced vital capacity Z-score		Respiratory	-0.003	0.004	0.420	0.919				
Forced vital capacity Z-score		Respiratory	0.005	0.005	0.293	0.853				
Hemoptysis	516.1	Respiratory	0.027	0.061	0.659	0.977	0.306	0.356	0.391	0.949
Hyperventilation	513.4	Respiratory	-0.186	0.236	0.430	0.919				
Hypoventilation	513.3	Respiratory	0.445	0.450	0.323	0.874				
Idiopathic pulmonary fibrosis		Respiratory	0.278	0.259	0.282	0.833				
Influenza	481	Respiratory	0.000	0.088	0.996	1.000				
Lung disease due to external agents	500	Respiratory	-0.229	0.368	0.533	0.935				
Nasal polyps	471	Respiratory	0.008	0.050	0.866	0.987	0.544	0.466	0.243	0.916
Obstructive chronic bronchitis	496.21	Respiratory	-0.016	0.046	0.733	0.977				
Other alveolar and parietoalveolar pneumonopathy	504	Respiratory	0.159	0.143	0.267	0.813				
Other diseases of lung	510	Respiratory	0.168	0.105	0.110	0.624				

Other diseases of respiratory system, NEC	519.8	Respiratory	0.090	0.032	0.005	0.097	-0.141	0.253	0.577	0.968
Other diseases of respiratory system, not elsewhere classified	519	Respiratory	-1.130	0.602	0.061	0.538				
Other dyspnea	512.9	Respiratory	0.047	0.102	0.647	0.970				
Other pulmonary inflamation or edema	505	Respiratory	-0.526	0.325	0.105	0.621				
Other upper respiratory disease	479	Respiratory	-0.136	0.051	0.008	0.140	-0.939	0.474	0.048	0.706
Painful respiration	512.2	Respiratory	-0.022	0.166	0.896	0.987				
Paralysis/spasm of vocal cords or larynx	473.3	Respiratory	0.292	0.175	0.096	0.605				
Pleurisy; pleural effusion	507	Respiratory	0.056	0.050	0.266	0.813	-0.285	0.420	0.496	0.967
Pneumococcal pneumonia	480.11	Respiratory	0.007	0.030	0.824	0.979	0.038	0.226	0.866	0.991
Pneumoconiosis	500.2	Respiratory	-0.611	0.536	0.254	0.807				
Pneumonia	480	Respiratory	-0.045	0.037	0.224	0.780	0.435	0.233	0.061	0.706
Pneumonia due to fungus (mycoses)	480.3	Respiratory	-0.269	0.275	0.328	0.874				
Pneumonitis due to inhalation of food or vomitus	501	Respiratory	-0.217	0.102	0.034	0.393				
Postinflammatory pulmonary fibrosis	502	Respiratory	-0.015	0.108	0.889	0.987				
Pseudomonal pneumonia	480.12	Respiratory	0.214	0.246	0.382	0.906				
Pulmonary collapse; interstitial and compensatory emphysem	508	Respiratory	0.142	0.150	0.345	0.890				
Pulmonary congestion and hypostasis	503	Respiratory	-0.046	0.234	0.845	0.983				
Respiratory complications	519.2	Respiratory	-0.189	0.295	0.522	0.935				
Respiratory failure	509.1	Respiratory	-0.114	0.115	0.320	0.874				
Respiratory insufficiency	509.2	Respiratory	-0.219	0.135	0.104	0.621				
Sarcoidosis		Respiratory	-0.019	0.133	0.887	0.987				
Septal Deviations/Turbinate Hypertrophy	470	Respiratory	-0.012	0.043	0.774	0.979	-0.149	0.463	0.748	0.991
ShortBreath		Respiratory	0.070	0.025	0.005	0.101	-0.207	0.111	0.063	0.706
Shortness of breath	512.7	Respiratory	0.095	0.039	0.014	0.203	0.172	0.239	0.472	0.964
Symptoms involving respiratory system and other chest symp	519.9	Respiratory	-0.021	0.171	0.901	0.987				
Throat pain	478	Respiratory	0.301	0.137	0.028	0.337				
Viral pneumonia	480.2	Respiratory	-0.305	0.251	0.225	0.780				
Voice disturbance	473.4	Respiratory	0.074	0.096	0.440	0.922				
Wheezing	512.1	Respiratory	0.394	0.328	0.229	0.785				
Amblyopia		Sense Organs	-0.056	0.088	0.526	0.935				
Aphakia and other disorders of lens	379.3	Sense Organs	0.026	0.040	0.517	0.935	0.110	0.311	0.723	0.991
Astigmatism	367.2	Sense Organs	-0.177	0.307	0.564	0.944				
Astigmatism		Sense Organs	0.000	0.065	0.995	1.000	-0.081	0.404	0.841	0.991
Average mean spherical equivalent refractive error		Sense Organs	-0.008	0.010	0.414	0.917	0.002	0.041	0.956	0.995
Blindness and low vision	367.9	Sense Organs	-0.047	0.289	0.870	0.987				
Cataract	366	Sense Organs	-0.023	0.019	0.229	0.785	0.022	0.108	0.840	0.991
Cataract		Sense Organs	0.035	0.030	0.242	0.790	0.341	0.156	0.029	0.619
Cataract surgery		Sense Organs	-0.048	0.067	0.471	0.925	0.198	0.253	0.433	0.949
Cholesteatoma	385.3	Sense Organs	0.162	0.107	0.131	0.648				
Chorioretinal inflammations, scars, and other disorders of ch	363	Sense Organs	0.272	0.311	0.381	0.906				
Cochlear implant		Sense Organs	-0.169	0.267	0.528	0.935				
Conductive hearing loss	389.2	Sense Organs	0.241	0.162	0.137	0.653				
Conjunctivitis, infectious	369.5	Sense Organs	0.232	0.385	0.546	0.935				
Conjunctivitis, noninfectious	371.2	Sense Organs	0.014	0.461	0.976	0.994				
Corneal degenerations	364.4	Sense Organs	-0.371	0.328	0.259	0.810				
Corneal dystrophy	364.5	Sense Organs	0.012	0.183	0.949	0.990				
Corneal edema	364.2	Sense Organs	-0.156	0.351	0.657	0.976				
Corneal graft surgery		Sense Organs	0.183	0.205	0.371	0.906				
Corneal hysteresis, left eye		Sense Organs	-0.012	0.010	0.215	0.766	-0.039	0.044	0.371	0.946
Corneal hysteresis, right eye		Sense Organs	-0.002	0.010	0.860	0.987	-0.030	0.043	0.489	0.967
Corneal opacity	364.1	Sense Organs	0.013	0.459	0.978	0.996				
Corneal opacity and other disorders of cornea	364	Sense Organs	-0.148	0.210	0.479	0.925				
Corneal ulcer	370.1	Sense Organs	0.205	0.210	0.330	0.874				
Deaf		Sense Organs	-0.611	0.355	0.085	0.587				
Diabetic eye disease		Sense Organs	-0.020	0.058	0.734	0.977	0.033	0.198	0.866	0.991
Diplopia and disorders of binocular vision	368.2	Sense Organs	0.273	0.150	0.069	0.545				
Disorders of conjunctiva	372	Sense Organs	-0.105	0.107	0.328	0.874	0.106	0.274	0.698	0.991
Disorders of external ear	380	Sense Organs	-0.019	0.099	0.845	0.983				
Disorders of iris and ciliary body	379.5	Sense Organs	0.224	0.246	0.363	0.902				
Disorders of lacrimal system	375	Sense Organs	0.084	0.064	0.192	0.729	0.649	0.374	0.083	0.732
Disorders of optic nerve and visual pathways	377	Sense Organs	-0.316	0.413	0.445	0.922				
Disorders of refraction and accommodation; blindness and lo	367	Sense Organs	-0.346	0.386	0.370	0.906				
Disorders of the globe	360	Sense Organs	-0.829	0.610	0.174	0.717				
Disorders of the orbit	376	Sense Organs	-0.009	0.265	0.974	0.994				
Disorders of vitreous body	379.2	Sense Organs	0.012	0.094	0.894	0.987	0.663	0.478	0.165	0.809
Dizziness and giddiness (Light-headedness and vertigo)	386.9	Sense Organs	0.134	0.047	0.004	0.094	-0.273	0.298	0.359	0.946
Ectropion or entropion	374.1	Sense Organs	-0.070	0.068	0.303	0.862				
Epiphora	375.2	Sense Organs	0.018	0.089	0.835	0.979	0.576	0.464	0.215	0.870
Eustachian tube disorders	381.2	Sense Organs	0.153	0.181	0.398	0.906				
Eye infection, viral	369.2	Sense Organs	0.204	0.257	0.428	0.919				
Eye surgery		Sense Organs	-0.039	0.035	0.268	0.813	-0.016	0.169	0.925	0.995
Glaucoma	365	Sense Organs	-0.035	0.068	0.609	0.965	-0.419	0.386	0.279	0.922
Glaucoma		Sense Organs	0.003	0.041	0.934	0.990	-0.147	0.203	0.470	0.964
Glaucoma eye surgery		Sense Organs	0.051	0.265	0.847	0.984				
Glaucoma laser treatment		Sense Organs	0.024	0.184	0.896	0.987				
Hearing aid		Sense Organs	0.006	0.026	0.817	0.979	0.007	0.305	0.982	0.996
Hearing loss	389	Sense Organs	-0.288	0.115	0.012	0.188				
Hypermetropia	367.8	Sense Organs	-0.440	0.383	0.250	0.804				
Hypermetropia		Sense Organs	-0.001	0.024	0.965	0.994	0.138	0.106	0.192	0.830
Impacted cerumen	380.4	Sense Organs	-0.170	0.199	0.393	0.906				
Infection of the eye	369	Sense Organs	-0.105	0.183	0.566	0.944				
Inflammation of eyelids	371.3	Sense Organs	-0.117	0.060	0.049	0.482	0.146	0.350	0.678	0.991
Inflammation of the eye	371	Sense Organs	0.385	0.315	0.222	0.779				
Keratitis	370	Sense Organs	0.144	0.278	0.604	0.965				
Keratoconus	364.41	Sense Organs	-0.157	0.320	0.623	0.965				
Labyrinthitis	386.3	Sense Organs	0.223	0.086	0.010	0.154	-0.426	0.630	0.499	0.967
Lagophthalmos	374.2	Sense Organs	0.640	0.463	0.167	0.711				
Laser eye surgery		Sense Organs	-0.055	0.068	0.414	0.917	-0.217	0.395	0.583	0.971
Macular degeneration		Sense Organs	0.018	0.058	0.761	0.979	0.553	0.353	0.117	0.774
Macular degeneration (senile) of retina NOS	362.29	Sense Organs	-0.029	0.058	0.620	0.965	-0.245	0.370	0.508	0.967
Mastoiditis & related conditions	381.3	Sense Organs	0.244	0.219	0.265	0.813				
Mean intraoccular pressure, Goldmann-correlated		Sense Organs	-0.006	0.010	0.563	0.944	0.033	0.042	0.427	0.949
Mean intraoccular pressure, corneal-compensated		Sense Organs	-0.001	0.010	0.891	0.987	0.039	0.042	0.348	0.946
Meniere's disease	386.1	Sense Organs	0.095	0.184	0.608	0.965				
Myopia	367.1	Sense Organs	0.339	0.269	0.208	0.754				
Myopia		Sense Organs	-0.002	0.020	0.903	0.987	-0.156	0.101	0.123	0.774
Nonsenile Cataract	366.1	Sense Organs	0.067	0.314	0.830	0.979				
Optic neuritis/neuropathy	377.3	Sense Organs	0.330	0.229	0.151	0.694				
Otalgia	382	Sense Organs	0.068	0.189	0.718	0.977				
Other disorders of ear	388	Sense Organs	-0.249	0.317	0.433	0.922				
Other disorders of eye	379	Sense Organs	0.118	0.170	0.488	0.925				
Other disorders of eyelids	374	Sense Organs	0.013	0.046	0.781	0.979	0.149	0.368	0.686	0.991
Other disorders of middle ear and mastoid	385	Sense Organs	-0.211	0.253	0.403	0.910				
Other disorders of tympanic membrane	384	Sense Organs	0.030	0.191	0.876	0.987				
Other nondiabetic retinopathy	362.3	Sense Organs	0.323	0.389	0.407	0.913				
Other retinal disorders	362	Sense Organs	-0.049	0.096	0.611	0.965	-0.859	0.753	0.254	0.916
Otitis externa	380.1	Sense Organs	0.081	0.117	0.489	0.925				
Otitis media	381.1	Sense Organs	0.040	0.079	0.610	0.965				
Otorrhea	381.9	Sense Organs	-0.124	0.203	0.543	0.935				

Otosclerosis	383	Sense Organs	-0.031	0.160	0.849	0.984				
Pain, swelling or discharge of eye	379.9	Sense Organs	0.172	0.245	0.482	0.925				
Paralytic strabismus	378.5	Sense Organs	0.262	0.173	0.130	0.646				
Perforation of tympanic membrane	384.4	Sense Organs	-0.082	0.092	0.376	0.906				
Peripheral or central vertigo	386.2	Sense Organs	0.177	0.109	0.106	0.621				
Peripheral retinal degenerations	362.6	Sense Organs	0.064	0.377	0.866	0.987				
Presbyopia		Sense Organs	0.002	0.021	0.915	0.987	0.025	0.103	0.811	0.991
Primary angle-closure glaucoma	365.2	Sense Organs	-0.036	0.098	0.712	0.977				
Primary open angle glaucoma	365.11	Sense Organs	0.049	0.080	0.543	0.935	0.003	0.297	0.992	0.996
Ptosis of eyelid	374.3	Sense Organs	0.017	0.057	0.774	0.979	0.050	0.404	0.901	0.995
Retinal detachment with retinal defect	361.1	Sense Organs	-0.162	0.064	0.011	0.170	0.628	0.471	0.183	0.825
Retinal detachments and defects	361	Sense Organs	0.046	0.055	0.403	0.910	0.442	0.455	0.331	0.946
Retinal hemorrhage/ischemia	362.8	Sense Organs	0.507	0.362	0.162	0.706				
Retinal vascular changes and abnomalities	362.4	Sense Organs	0.072	0.094	0.447	0.922				
Retinoschisis and retinal cysts	361.2	Sense Organs	-0.221	0.550	0.688	0.977				
Senile cataract	366.2	Sense Organs	0.023	0.020	0.260	0.812	0.053	0.143	0.711	0.991
Sensorineural hearing loss	389.1	Sense Organs	0.236	0.174	0.175	0.718				
Separation of retinal layers	362.31	Sense Organs	0.123	0.316	0.697	0.977				
Speech-reception-threshold estimate (left)		Sense Organs	-0.002	0.007	0.832	0.979	-0.034	0.038	0.370	0.946
Speech-reception-threshold estimate (right)		Sense Organs	0.006	0.007	0.385	0.906	-0.032	0.038	0.400	0.949
Strabismus		Sense Organs	-0.004	0.109	0.971	0.994				
Strabismus (not specified as paralytic)	378.1	Sense Organs	0.151	0.085	0.076	0.568				
Strabismus and other disorders of binocular eye movements	378	Sense Organs	-0.090	0.394	0.819	0.979				
Subjective visual disturbances	368.9	Sense Organs	0.459	0.184	0.013	0.193				
Suppurative and unspecified otitis media	381.11	Sense Organs	-0.114	0.105	0.279	0.830				
Tinnitus	389.4	Sense Organs	-0.555	0.324	0.086	0.589				
Tinnitus		Sense Organs	0.001	0.034	0.985	0.997	0.097	0.167	0.559	0.967
Traumatic cataract	366.3	Sense Organs	0.048	0.253	0.849	0.984				
Tympanosclerosis and middle ear disease related to otitis me	385.5	Sense Organs	-0.114	0.262	0.664	0.977				
Uveitis, noninfectious or NOS	371.1	Sense Organs	0.175	0.200	0.380	0.906				
Vertiginous syndromes and other disorders of vestibular syste	386	Sense Organs	0.120	0.294	0.684	0.977				
Visual acuity, left eye		Sense Organs	-0.014	0.009	0.124	0.646	-0.015	0.039	0.698	0.991
Visual acuity, right eye		Sense Organs	-0.002	0.009	0.860	0.987	-0.046	0.039	0.247	0.916
Visual disturbances	368	Sense Organs	-0.030	0.122	0.803	0.979				
Visual field defects	368.4	Sense Organs	-0.101	0.278	0.716	0.977				
Abdominal pain	785	Symptoms	-0.004	0.014	0.805	0.979	0.050	0.084	0.551	0.967
Back pain	760	Symptoms	-0.017	0.027	0.536	0.935	0.050	0.145	0.730	0.991
Cervical radiculitis	765	Symptoms	0.004	0.114	0.970	0.994				
Cervicalgia	761	Symptoms	-0.104	0.069	0.130	0.646	-2.387	1.017	0.019	0.537
Chronic fatigue syndrome	798.1	Symptoms	-0.733	0.434	0.091	0.599				
Edema	782.3	Symptoms	0.043	0.109	0.693	0.977				
Fever of unknown origin	783	Symptoms	0.083	0.056	0.138	0.653	-0.358	0.393	0.362	0.946
Gangrene	791	Symptoms	-0.240	0.240	0.319	0.874				
Hypothermia/Chills	780	Symptoms	0.051	0.327	0.877	0.987				
Malaise and fatigue	798	Symptoms	0.011	0.069	0.877	0.987	0.213	0.352	0.545	0.967
Muscular wasting and disuse atrophy	772.1	Symptoms	0.619	0.434	0.153	0.694				
Musculoskeletal symptoms referable to limbs	771	Symptoms	-0.143	0.101	0.157	0.700	0.258	0.488	0.597	0.976
Myalgia and myositis unspecified	770	Symptoms	0.053	0.105	0.610	0.965	-0.108	0.513	0.833	0.991
Nausea and vomiting	789	Symptoms	0.010	0.043	0.822	0.979	-0.184	0.266	0.489	0.967
Neuralgia, neuritis, and radiculitis NOS	766	Symptoms	0.082	0.069	0.235	0.787	0.339	0.357	0.343	0.946
Nonspecific findings on examination of blood	790	Symptoms	0.156	0.380	0.682	0.977				
Other abnormal blood chemistry	790.6	Symptoms	0.100	0.040	0.013	0.197	0.132	0.237	0.576	0.968
Pallor and flushing	782.6	Symptoms	-0.061	0.373	0.869	0.987				
Rhabdomyolysis	772.4	Symptoms	-0.979	0.742	0.187	0.720				
Sciatica	764	Symptoms	-0.068	0.067	0.309	0.863	-0.258	0.373	0.489	0.967
Swelling of limb	771.1	Symptoms	-0.032	0.039	0.418	0.919	0.128	0.220	0.561	0.967
Symptoms involving nervous and musculoskeletal systems	781	Symptoms	0.094	0.070	0.179	0.720	-0.258	0.494	0.601	0.977
Symptoms of the muscles	772	Symptoms	-0.042	0.128	0.741	0.977				
Syncope and collapse	788	Symptoms	-0.007	0.029	0.797	0.979	0.355	0.188	0.059	0.706
